# Supplementary material for: Methodological concerns underlying a lack of evidence for cultural heterogeneity in the replication of psychological effects
Source: Commun Psychol. 2024 Oct 8;2:93. doi: 10.1038/s44271-024-00135-z (PMC11461273; doi:10.1038/s44271-024-00135-z)
Supplement: Supplementary file 2 — Supplementary Information [file 44271_2024_135_MOESM2_ESM.pdf]

Supplementary Information

Methodological Concerns Underlying a Lack of Evidence for Cultural Heterogeneity in the Replication of Psychological Effects

Robin Schimmelpfennig   Rachel Spicer   Cindel White   Will Gervais*
Ara Norenzayan   Steven Heine   Joseph Henrich   Michael Muthukrishna

Contents

1 Supplementary Methods **2**
1.1 Simulation . . . 2
1.1.1 Simulation Setup and Functions . . . 2
1.1.2 Simulation Software . . . 13
1.2 ML2 - Reanalysis: Methods . . . 14
1.2.1 Cultural Distance . . . 14
1.2.2 Analysis Plan . . . 15
1.2.3 Reanalysis Software . . . 20

2 Supplementary Results **21**
2.1 Additional Simulation Results . . . 21
2.2 Description of ML-2 Sample . . . 23
2.2.1 Overview of Distribution of Birth Countries . . . 23
2.2.2 Movement From Birth Countries to Sample Countries . . . 29
2.2.3 Migrant populations in ML2 . . . 37
2.2.4 CF_{ST} Scores for Slate 2 . . . 39
2.3 ML2 - Reanalysis: Full Results . . . 40
2.3.1 Analysis A Results . . . 40
2.3.2 Analysis B Results . . . 45
2.3.3 Analysis C Results . . . 87
2.3.4 Analysis D Results . . . 119
2.3.5 Analysis E Results . . . 123

Supplementary References **128**

*Wrote the simulation software.

Supplementary Table 1. Overview of the functions in the simulation. The simulation code can be accessed at: https://osf.io/b3dxr/

sim_many_ml2(...)	Run repeated simulated Many Labs 2 projects, splitting out overall summary statistics about the proportion of effects showing significant heterogeneity, etc.
sim_multi_ml2(...)	Summarize a given Many Labs 2 project; how many effects show cultural moderation or heterogeneity?
sim_ml2(...)	Simulate a given Many Labs 2 project producing summarized output for each study.
sim_many_metaanalyses(...)	Gets meta-analytic summaries for all the different samples' runs on a given effect.
sim_metaanalysis(...)	Runs a meta-analysis (and stores relevant output) for a single effect, run across a bunch of samples.
sim_manyLab(...)	For one given effect, select a series of countries for individual samples, simulating the data for a Many Lab project on an effect.
sim_study(...)	Figures out the effect size in a given country, then simulates a study from that effect, and stores the observed effect size information.

1 Supplementary Methods

1.1 Simulation

1.1.1 Simulation Setup and Functions

To begin with, we created a simulation environment in which multiple studies in different settings or countries are run on a given effect. Countries in the simulation were randomly drawn, in proportion to their representation in ML2. We re-created the ML2-WEIRDness scores, performed their same mean split into WEIRDER and less WEIRD countries, and meta-analytically quantified heterogeneity exactly as they did, using Q, tau, and I^2 indices (Borenstein et al., 2009).

Simulations always include assumptions, and we strove to model all assumptions both transparently and generously. Representation of countries and effect sizes directly maps onto ML2's design. To manipulate the levels of heterogeneity in the study, we model both cultural differences (captured by CF_{ST}; Muthukrishna et al. (2020)) between countries, and the moderating role of these cultural differences of the effects captured by the variable cultural influence (see explanation in the main paper).

The simulations involve several nested functions. The simulation layout is overall set up so that lower list functions are called hierarchically by higher ones. The functions of the simulation are presented below in Table S1.

1.1.1.1 sim.study(...) Firstly, `sim.study(...)` is a function that picks a country from the Many Labs 2 roster (weighted by their actual frequency), applies moderation by culture according to strength of simulated cultural influence and simulates data, determining the effect size of the country.

This function builds a roster of Many Labs 2 countries, cultural distance (per Muthukrishna et al. (2020)), and also includes the ML2-WEIRDness composite score from ML2. As an input, it asks how strong you want `cultural.influence` to be. `cultural.influence` is implemented so that the input value reflects how much lower you want the effect size to be in a country that's most dissimilar from the USA (within ML-2's sampling frame), relative to the USA. Essentially, the `cultural.influence` input tells the simulations how different you want the Czech Republic samples to be from the USA samples, with all other samples falling in line linearly, according to cultural distance (Muthukrishna et al., 2020). Details can be found in the main paper. In our opinion, this is a generous assumption about how culture works, and is thus a highly idealized world we're simulating. There's no theoretical reason to assume that all psychological effects vary linearly with culture, per the index that Muthukrishna et al. (2020) generated, much less for the ML2 binary ML2-WEIRDness variable, so interpret output with appropriate restraint. If the influence of culture is more complex and iterative than this, which it almost certainly is, this will probably drive down the power for everything else. These simulations thus represent a sort of best case scenario for detecting culture in a Many Labs type environment.

A `cultural.influence` setting of 1 means that the effect size will be 100% smaller in Czech Republic (the country most distant from the USA in Many Labs 2) than the USA: the effect disappears. A `cultural.influence` setting of 0.5 halves the effect size in Czech Republic relative to USA.

The below code shows effect sizes in some sample countries, under different simulated culture regimes.

```
# Simulate study. This function:
# 1 - selects a country for the study
# 2 - adjusts the true effect size per cultural.influence and culture parameters
# 3 - samples data for the study
# 4 - calculates and stores observed effect size info for meta-analysis
# @param real.d: true value of Cohen's d
# @param cultural.influence: level of cultural moderation
# @param culture: cultural influence adjustment or not. Setting `culture=F` will keep all
↪  samples from the same country the same in terms of true effect size.
sim_study <- function(real.d, cultural.influence, culture) {

  # Build a frame with ML2 countries (Country), frequency (samples), the non-construct ML-2
  ↪  dubbed "WEIRDness" (WEIRDness), and cultural distance from USA per Muthukrishna et al
  ↪  (CFst)
Country <- c("Australia","Austria","Belgium","Brazil","Canada","Chile","China","Colombia","Costa
↪  Rica","Czech Republic","France","Germany","Hong
↪  Kong","Hungary","India","Italy","Japan","Malaysia","Mexico","Netherlands","New
↪  Zealand","Nigeria","Poland","Portugal","Serbia","South
↪  Africa","Spain","Sweden","Switzerland","Taiwan","Tanzania","Turkey","United Arab
↪  Emirates","United Kingdom","United States","Uruguay")
  samples <- c(3,7,2,1,5,3,6,1,3,3,2,5,4,2,5,1,1,1,1,10,2,1,6,1,3,3,3,1,1,2,2,3,2,4,43,1)
  WEIRDness <-
↪  c(0.82,0.86,0.82,0.48,0.82,0.7,0.29,0.45,0.49,0.8,0.8,0.89,0.32,0.74,0.21,0.8,0.67,0.27,0.47,
0.85,0.82,0.37,0.75,0.74,0.47,0.47,0.78,0.88,0.94,0.29,0.39,0.27,0.29,0.81,0.84,0.51)
  cfst <- c(0.033, NA, NA, 0.071, 0.030, 0.080, 0.166, 0.105, NA, 0.191, 0.077, 0.069, 0.073,
↪  0.126, 0.136, 0.071, 0.115, 0.118, 0.084, 0.076, 0.054, 0.169, 0.100, NA, 0.084, 0.078,
↪  0.078, 0.115, 0.066, 0.100, 0.158, 0.118, NA, 0.044, 0, 0.096)
  df <- data.frame(Country, samples, WEIRDness, cfst)
  # Translate the samples to probability of sample getting picked
  df <- df %>%
    mutate(prSample = samples/sum(df$samples)) %>%
    filter(!is.na(cfst))

  real.d <- real.d

  # Per-cell sample sizes, form 50-200
  n <- round(runif(1, min = 50, max = 200), 0)

  # Pick a site
  site <- df %>% slice_sample(n = 1, weight_by = prSample, replace = T)

  # Turn CFst into an adjustment multiplier for culture
  cult <- 1 - (site$cfst * cultural.influence / max(df$cfst))

  # Make additional cultural influence adjustments if wanted
  if(culture == T) {mod <- rnorm(1, mean = 1, sd = .1)}
  if(culture == F) {mod <- 1}

  # Calculate site's effect size, per cultural distance and cultural influence
  site.g <- real.d * cult * mod

  # Simulate a study based on effect size expected, hg
```

```r
  cond <- n/2
  control.c <- rnorm(cond, mean = 0, sd = 1)
  exp.c <- rnorm(cond, mean = site.g, sd = 1)

  # Calculate effect size
  g_m <- escalc(measure = "SMD",
                m1i = mean(exp.c),
                sd1i = sd(exp.c),
                n1i = cond,
                m2i = mean(control.c),
                sd2i = sd(control.c),
                n2i = cond)

  # Grab effect size info for eventual meta-analysis
  g.obs <- g_m$yi
  var.g.obs <- g_m$vi

  # Store output
  fr <- data.frame(country = site$Country, WEIRDness = site$WEIRDness, cfst = site$cfst,
↪   culture, real.d, n, site.g, g.obs, var.g.obs)
  return(fr)
}
```

These are the results for cultural influence set to 1, for a true effect size of *d* = 0.5 in the USA.

```r
sim_study(real.d = 0.5, cultural.influence = 1, culture = F)
```

```
##    country WEIRDness  cfst culture real.d   n     site.g      g.obs  var.g.obs
## 1 Tanzania      0.39 0.158   FALSE    0.5 176 0.08638743 0.04789793 0.02273379
```

Note that the cultural influence value sets how dissimilar the *most dissimilar* countries are. Intermediate cultures fall in according to their relative positions on cultural distance (Muthukrishna et al., 2020), based on their relation to the most dissimilar country. `cultural.influence` is therefore a dial that sets how much *overall* spread the countries are, per the *relative* degree they differ in Muthukrishna et al. (2020).

1.1.1.2 sim_manyLab(...)

`sim_study(...)` simulates the results of a single study. `sim_manyLab(...)` repeats the process for each of `k` sites, simulating a single study in a Many Labs style research project. `pr.rep` and `culture` are very optional inputs for if you're trying to specify specific replication rates or build in within-country heterogeneity, which can safely be ignored for other simulation purposes.

```r
# Simulate Many Labs result. This function:
# 1 - simulates a Many Labs approach for a single effect
# 2 - for each of k participating sites
# 3 - per parameters for effect size, replicability, etc
# 4 - simulates a study for each site using repeats of sim_study()
# @param k: number of sites to include
# @param original.d: true effect size of USA
# @param cultural.influence: level of cultural moderation
# @param pr.rep: pr.rep = 1 means that every site starts with a true effect (d = .35, before
↪   cultural.influence)
# @param culture: cultural influence adjustment or not. Setting `culture=F` will keep all
↪   samples from the same country the same in terms of true effect size.
sim_manyLab <- function(k, original.d, cultural.influence, pr.rep, culture) {
  k <- k
  culture <- culture

  # Make it replicable, or not, per assigned probability
```

```r
  replicates <- sample(c(1,0), 1, replace = T, prob = c(pr.rep, 1 - pr.rep))
  original.d <- original.d

  # Set up bins to store output
  country <- numeric(k)
  WEIRDness <- numeric(k)
  cfst <- numeric(k)
  w.culture <- logical(k)
  target.d <- numeric(k)
  n <- numeric(k)
  site.g <- numeric(k)
  g.obs <- numeric(k)
  var.g.obs <- numeric(k)
  replicable <- rep(replicates, k)
  original <- rep(original.d, k)

  # Simulate the studies and store results
  for (i in 1:k) {

    real.d <- original.d * replicates

    dv <- sim_study(real.d = real.d, cultural.influence = cultural.influence, culture = culture)
    country[i] <- dv$country
    WEIRDness[i] <- dv$WEIRDness
    cfst[i] <- dv$cfst
    w.culture[i] <- dv$culture
    n[i] <- dv$n
    site.g[i] <- dv$site.g
    g.obs[i] <- dv$g.obs
    var.g.obs[i] <- dv$var.g.obs
  }

  # Combine results into a frame
  k.studies <- data.frame(country, WEIRDness, cfst, original, replicable, w.culture, n, site.g,
↪   g.obs, var.g.obs)
  return(k.studies)
}
```

Running `sim_study(...)` for 20 sites, we will produce an output similar to this:

```r
sim_manyLab(k = 20, original.d = 0.5, cultural.influence = 2, pr.rep = 1, culture = F)
```

```
##           country WEIRDness  cfst original replicable w.culture   n      site.g
## 1   United States      0.84 0.000      0.5          1     FALSE 133  0.50000000
## 2           China      0.29 0.166      0.5          1     FALSE 140 -0.36910995
## 3           China      0.29 0.166      0.5          1     FALSE  50 -0.36910995
## 4  Czech Republic      0.80 0.191      0.5          1     FALSE 169 -0.50000000
## 5   United States      0.84 0.000      0.5          1     FALSE 182  0.50000000
## 6          Brazil      0.48 0.071      0.5          1     FALSE 193  0.12827225
## 7          Canada      0.82 0.030      0.5          1     FALSE 122  0.34293194
## 8         Hungary      0.74 0.126      0.5          1     FALSE  57 -0.15968586
## 9          Taiwan      0.29 0.100      0.5          1     FALSE  64 -0.02356021
## 10         Sweden      0.88 0.115      0.5          1     FALSE 197 -0.10209424
## 11         Canada      0.82 0.030      0.5          1     FALSE 161  0.34293194
## 12  United States      0.84 0.000      0.5          1     FALSE 121  0.50000000
## 13         Canada      0.82 0.030      0.5          1     FALSE 152  0.34293194
## 14  United States      0.84 0.000      0.5          1     FALSE  71  0.50000000
```

```
## 15          India      0.21 0.136      0.5           1     FALSE 147 -0.21204188
## 16 United Kingdom      0.81 0.044      0.5           1     FALSE  52  0.26963351
## 17  United States      0.84 0.000      0.5           1     FALSE 109  0.50000000
## 18         Taiwan      0.29 0.100      0.5           1     FALSE  86 -0.02356021
## 19         Turkey      0.27 0.118      0.5           1     FALSE  89 -0.11780105
## 20 United Kingdom      0.81 0.044      0.5           1     FALSE 187  0.26963351
##          g.obs  var.g.obs
## 1   0.48739061 0.03096823
## 2  -0.73452751 0.03049832
## 3  -0.06810971 0.08004639
## 4  -0.49295408 0.02438758
## 5   0.69187383 0.02329310
## 6   0.01755055 0.02072619
## 7   0.32884759 0.03323008
## 8  -0.17991752 0.07045939
## 9   0.35968930 0.06351075
## 10 -0.05840279 0.02031323
## 11  0.57360349 0.02586652
## 12  0.38362307 0.03366598
## 13  0.43784466 0.02694641
## 14  0.89455435 0.06197343
## 15 -0.38852224 0.02772432
## 16 -0.01626655 0.07692562
## 17  0.29064422 0.03708474
## 18 -0.07497649 0.04654431
## 19  0.14939471 0.04506921
## 20  0.26141301 0.02157309
```

The `original.d` reflects the USA effect size – the original effect size you've either specified or had selected from the ML-2 roster. The output variable `site.g` is the effect size in each site, after running through the culture process and `g.obs` is the observed effect size.

1.1.1.3 sim_metaanalysis(...)

`sim_metaanalysis(...)` is a function to run a meta-analysis on a batch of Many-Lab style results on a single effect, given `k` sites. The function stores the types of outputs that make up the results summarized in ML-2 e.g., the moderation tests use ML-2's ML2-WEIRDness strategy. `ml2.es = T` selects an effect size at random from Many Labs 2. `d.lo` and `d.hi` are parameters for if you want to specify your own effect sizes, selected from a uniform distribution with these bounds.

```
# Perform meta-analysis.
# This function runs a meta-analysis for a single cross many-labs study simulation resulting
↪  from sim_manyLab()
# (It simulates a single ML-2 study)
# 1 - you can if select the effect sizes from actual ML-2 results (ml2.es = T)
# 2 - or you can select effect sizes at random, from a uniform bounded by d.lo = XX and d.hi =
↪  YY
# 3 - if ml2.es = T, d.lo and d.hi must = NULL
# @param k: number of sites to include
# @param ml2.es: use original ML-2 effect sizes or not. Setting `ml2.es=T` will use the original
↪  ml2 effect sizes.
# @param d.lo: minimum value of Cohen's d
# @param d.hi: maximum value of Cohen's d
# @param cultural.influence: level of cultural moderation
# @param pr.rep: pr.rep = 1 means that every site starts with a true effect (d = .35, before
↪  cultural.influence)
# @param culture: cultural influence adjustment or not. Setting `culture=F` will keep all
↪  samples from the same country the same in terms of true effect size.
sim_metaanalysis <- function(k, ml2.es, d.lo = NULL, d.hi = NULL, cultural.influence, pr.rep,
↪  culture) {
```

```r
# Select effect size settings
# If ml2.es = T select the effect sizes from the original ML-2 results
if(ml2.es == T) {
  original.d <- sample(x = c(.05, -.07, 1.82, 1.75, 1.35, 1.18, .95, .95, .86, .78, .4, .4,
↪ .29, .18, .25, .12, .04, -.03, 0, .03, .01, -.04, -.03, -.02, -.03, -.13, -.08, -.18), 1,
↪ replace = T)
}
# If ml2.es = F select effect sizes at random, from a uniform bounded by d.lo and d.hi
if(ml2.es == F) {
  original.d <- round(runif(1, d.lo, d.hi), 2)
}

# Run and store the Many Labs simulation
ml <- sim_manyLab(k, original.d, cultural.influence, pr.rep, culture)

# Calculate WEIRDness, per simulated ML-2 studies slate
mWEIRD <- mean(ml$WEIRDness)

# Split countries in WEIRD and less WEIRD, using a mean split (as performed in the original
↪  ML-2 analysis)
ml$WEIRD <- ifelse(ml$WEIRDness > mWEIRD, 1, 0)

# Run a basic meta-analysis
ma.ml <- rma(yi = ml$g.obs, vi = ml$var.g.obs)
ml.conf <- confint(ma.ml)

# Run a second model with the WEIRDness moderator
ma.ml.mod <- rma(yi = ml$g.obs, vi = ml$var.g.obs, mods = ml$WEIRD)
ml.conf.mod <- confint(ma.ml.mod)

# Store meta analysis model results
original.d <- original.d
k <- k
cultural.influence <- cultural.influence
pr.rep <- pr.rep
culture <- culture
I2 <- ma.ml$I2
I2.lo <- ml.conf$random[7]
I2.hi <- ml.conf$random[11]
I2.nonZero <- ifelse(I2.lo > 0, 1, 0)
I2.over.50 <- ifelse(I2 > .5, 1, 0)
tau2 <- ma.ml$tau2
tau <- sqrt(tau2)
QE <- ma.ml$QE
QEp <- ma.ml$QEp
Q.sig.001 <- ifelse(QEp < .001, 1, 0) # Using ml-2's threshold adjustments
I2.mod <- ma.ml.mod$I2
I2.lo.mod <- ml.conf.mod$random[7]
I2.hi.mod <- ml.conf.mod$random[11]
I2.nonZero.mod <- ifelse(I2.lo.mod > 0, 1, 0)
tau2.mod <- ma.ml.mod$tau2
tau.mod <- sqrt(tau2.mod)
QE.mod <- ma.ml.mod$QE
QEp.mod <- ma.ml.mod$QEp
QM <- ma.ml.mod$QM
```

```
  QMp <- ma.ml.mod$QMp
  mod.sig <- ifelse(QMp < .004, 1, 0) # Using ML-2's correct p-val

  # Store output
  manymeta <- data.frame(original.d, k, cultural.influence, pr.rep, culture, I2, I2.lo, I2.hi,
↪   I2.nonZero, I2.over.50, tau2, tau, QE, QEp, Q.sig.001, mod.sig)
  return(manymeta)
}
```

Run it once to get results simulating the output for a single "effect" in a Many Labs project:

```
sim_metaanalysis(k = 20, ml2.es = T, d.lo = NULL, d.hi = NULL, cultural.influence = 2, pr.rep =
↪  1 , culture = F)
```

```
##   original.d  k cultural.influence pr.rep culture       I2    I2.lo    I2.hi
## 1        0.4 20                  2      1   FALSE 63.04271 34.39953 81.29886
##   I2.nonZero I2.over.50       tau2       tau       QE          QEp Q.sig.001
## 1          1          1 0.05427619 0.2329725 52.98727 4.701665e-05         1
##   mod.sig
## 1       0
```

1.1.1.4 sim_many_metaanalyses(...)

`sim_metaanalysis(...)` runs a meta-analysis on a Many Labs project's single effect.

`sim_many_metaanalyses(...)` runs that function several times and stores the associated results. This function is thus useful for simulating several single-effect Many Lab outcomes to gauge test performance over repeated iterations.

```
# Simulate many meta-analyses
# This function runs sim_metaanalysis() nMeta times, facilitating simulations
# It can be used for investigating the power of heterogeneity tests and such, given different
↪  effect sizes
# @param nMeta: number of meta-analysis runs to include
# @param k: number of sites to include
# @param ml2.es: use original ML-2 effect sizes or not. Setting `ml2.es=T` will use the original
↪  ml2 effect sizes.
# @param d.lo: minimum value of Cohen's d
# @param d.hi: maximum value of Cohen's d
# @param cultural.influence: level of cultural moderation
# @param pr.rep: pr.rep = 1 means that every site starts with a true effect (d = .35, before
↪  cultural.influence)
# @param culture: cultural influence adjustment or not. Setting `culture=F` will keep all
↪  samples from the same country the same in terms of true effect size.
# @param silent: whether to display notifications for the running of each simulation or not
sim_many_metaanalyses <- function(nMeta, k, ml2.es, d.lo, d.hi, cultural.influence, pr.rep,
↪  culture, silent) {

  bin <- data.frame() #
  for (j in 1:nMeta) {
    if(silent == F) {
      print(paste0("Running Meta ", j, "/", nMeta))
    }
    bin <- rbind(bin, sim_metaanalysis(k = k, ml2.es = ml2.es, d.lo = d.lo, d.hi = d.hi,
↪   cultural.influence = cultural.influence, pr.rep = pr.rep, culture = culture ))
  }

  return(bin)
```

```
}
```

Output for running the functions over three iterations:

```
sim_many_metaanalyses(nMeta = 3, k = 20, ml2.es = T, d.lo = NULL, d.hi = NULL, pr.rep = 1,
↪  culture = F, silent = F, cultural.influence = 2)
```

```
## [1] "Running Meta 1/3"
## [1] "Running Meta 2/3"
## [1] "Running Meta 3/3"
```

```
##   original.d  k cultural.influence pr.rep culture       I2    I2.lo    I2.hi
## 1       0.00 20                  2      1   FALSE 20.30889  0.00000 62.78372
## 2       0.25 20                  2      1   FALSE 67.99642 46.11738 87.29518
## 3       0.03 20                  2      1   FALSE 19.22463  0.00000 57.99057
##   I2.nonZero I2.over.50        tau2        tau       QE          QEp Q.sig.001
## 1          0          1 0.008470942 0.09203772 23.60812 2.116162e-01         0
## 2          1          1 0.065201975 0.25534677 58.27401 7.232568e-06         1
## 3          0          1 0.008539422 0.09240899 21.94864 2.868196e-01         0
##   mod.sig
## 1       0
## 2       0
## 3       0
```

From this it is possible to specify more specific outputs. For example, we could ask what the average power to detect moderation by ML2-WEIRDness is using `mean(d1$mod.sig)`, or what the average power to detect heterogeneity with a *Q* stat is, using `mean(d1$Q.sig.001)`.

1.1.1.5 sim_ml2(...)

`sim_ml2(...)` is a function that repeats the function `sim_many_metaanalyses(...)`, but for several different effect sizes. Essentially `sim_ml2(...)` is running a Many Labs - style study, with a full roster of different original effects (which can differ in magnitude). The parameter `ml2.es = T` simulates the original effect sizes for each effect from those actually observed in ML2, drawn with replacement. Across a lot of simulations, the function approximates the ML2 pattern of effect sizes, with many near zero, a few middling sized effects, and a few fairly substantial ones.

```
# Simulate a ML2 style study, with different effects.
# It is good for looking at results at the aggregate level of all effects in a ML project.
# This function:
# 1 - grabs a set number of `effects`
# 2 - will grab effects based on either ml-2 effect sizes, or by custom bounds
# 3 - calculates overall results of ML: what proportion of effects show evidence of
↪  moderation/heterogeneity
# @param effects: number of different effects to include
# @param k: number of sites to include
# @param ml2.es: use original ML-2 effect sizes or not. Setting `ml2.es=T` will use the original
↪  ml2 effect sizes.
# @param d.lo: minimum value of Cohen's d
# @param d.hi: maximum value of Cohen's d
# @param cultural.influence: level of cultural moderation
# @param pr.rep: pr.rep = 1 means that every site starts with a true effect (d = .35, before
↪  cultural.influence)
# @param culture: cultural influence adjustment or not. Setting `culture=F` will keep all
↪  samples from the same country the same in terms of true effect size.
sim_ml2 <- function(effects, k, ml2.es, d.lo, d.hi, cultural.influence, pr.rep, culture) {

  # Run sim_many_metaanalysis() once for each effect (nMeta = effects)
  mmm <- sim_many_metaanalyses(nMeta = effects, k = k, ml2.es = ml2.es, d.lo = d.lo, d.hi =
↪  d.hi, cultural.influence = cultural.influence, pr.rep = pr.rep, culture = culture, silent =
↪  TRUE)
```

```r
  # Store the results
  original.d <- mmm$original.d
  k <- mmm$k
  cultural.influence <- mmm$cultural.influence
  pr.rep <- mmm$pr.rep
  culture <- mmm$culture
  I2 <- mmm$I2
  I2.nonZero <- mmm$I2.nonZero
  I2.over.50 <- mmm$I2.over.50
  tau <- mmm$tau
  tau.10.plus <- ifelse(mmm$tau >= .10, 1, 0)
  QE <- mmm$QE
  Q.sig.001 <- mmm$Q.sig.001
  mod.sig.004 <- mmm$mod.sig

  # Build a data.frame
  ml2.frame <- data.frame(original.d, k, cultural.influence, pr.rep, culture, I2, I2.nonZero,
↪   I2.over.50, tau, tau.10.plus, QE, Q.sig.001, mod.sig.004)
  return(ml2.frame)
}
```

This is what the output looks like for 5 effects and 10 countries, based on ML2's observed effect sizes.

```r
set.seed(29)

sim_ml2(effects = 5, k = 10, ml2.es = T, d.lo = NULL, d.hi = NULL, cultural.influence = 2,
↪   pr.rep = 1, culture = F)
```

```
##   original.d  k cultural.influence pr.rep culture       I2 I2.nonZero
## 1       1.35 10                  2      1   FALSE 90.98194          1
## 2       0.40 10                  2      1   FALSE 72.28363          1
## 3       0.29 10                  2      1   FALSE 46.68911          0
## 4       0.18 10                  2      1   FALSE 22.54471          0
## 5       0.95 10                  2      1   FALSE 86.41750          1
##   I2.over.50       tau tau.10.plus       QE Q.sig.001 mod.sig.004
## 1          1 0.5910213           1 88.92172         1           0
## 2          1 0.3025221           1 30.37723         1           0
## 3          1 0.1697015           1 16.50100         0           0
## 4          1 0.1034123           1 10.82999         0           0
## 5          1 0.4968902           1 74.00012         1           1
```

1.1.1.6 sim_multi_ml2(...)

`sim_multi_ml2(...)` is a function to simulate and summarize the full ML-2 project. The output is the *proportion* of significant heterogeneity tests within a full docket of ML-2 effects. This is the high-level executive summary result that Many Labs 2 reports in the Results section: what proportion of effects showed significant heterogeneity?

```r
# Simulate multiple ML2
# Performs repeated Many Labs simulations
# It runs the overall simulation, with results at the per-ManyLabs project level
# @param effects: number of different effects to include
# @param k: number of sites to include
# @param ml2.es: use original ML-2 effect sizes or not. Setting `ml2.es=T` will use the original
↪   ml2 effect sizes.
# @param d.lo: minimum value of Cohen's d
# @param d.hi: maximum value of Cohen's d
# @param cultural.influence: level of cultural moderation
```

```r
# @param pr.rep: pr.rep = 1 means that every site starts with a true effect (d = .35, before
↪  cultural.influence)
# @param culture: cultural influence adjustment or not. Setting `culture=F` will keep all
↪  samples from the same country the same in terms of true effect size.
sim_multi_ml2 <- function(effects, k, ml2.es, d.lo, d.hi, cultural.influence, pr.rep, culture) {

  # simulate a bunch of ml-2s
  ml2 <- sim_ml2(effects = effects, k = k, ml2.es = ml2.es, d.lo = d.lo, d.hi = d.hi,
↪  cultural.influence = cultural.influence, pr.rep = pr.rep, culture = culture)

  # store some results
  lowest.d <- min(ml2$original.d)
  highest.d <- max(ml2$original.d)
  mean.d <- mean(ml2$original.d)
  effects <- nrow(ml2)
  k <- ml2$k[1]
  cultural.influence <- ml2$cultural.influence[1]
  culture <- ml2$culture[1]
  pr.I2.nonZero <- mean(ml2$I2.nonZero)
  pr.I2.over.50 <- mean(ml2$I2.over.50)
  tau.median <- median(ml2$tau)
  pr.tau.over.10 <- mean(ml2$tau.10.plus)
  pr.Q.sig.001 <- mean(ml2$Q.sig.001)
  pr.mod.sig.004 <- mean(ml2$mod.sig.004)

  ml2.perf <- data.frame(lowest.d, highest.d, mean.d, effects, k, cultural.influence, culture,
↪  tau.median, pr.tau.over.10, pr.I2.nonZero, pr.I2.over.50, pr.Q.sig.001, pr.mod.sig.004)
  return(ml2.perf)
}
```

Running the same parameters as for `sim_ml2(...)` simply produces a summary of the results:

```r
set.seed(29)

sim_multi_ml2(effects = 5, k = 10 , ml2.es = T, d.lo = NULL, d.hi = NULL, pr.rep = 1, culture =
↪  F, cultural.influence = 2)
```

```
##   lowest.d highest.d mean.d effects  k cultural.influence culture tau.median
## 1     0.18      1.35  0.634       5 10                  2   FALSE  0.3025221
##   pr.tau.over.10 pr.I2.nonZero pr.I2.over.50 pr.Q.sig.001 pr.mod.sig.004
## 1              1           0.6             1          0.6            0.2
```

1.1.1.7 sim_many_ml2(...) This function runs repeated simulations of the whole process and stores the output for each full ML-2 in a new row. This allows us to simulate the whole setup to see what overall patterns emerge with different, induced culture regimes. This is where you can see, for a given culture setting, how unusual results like those observed in Many Labs 2 are. `nML2` sets the number of simulations to run, however this can be time consuming and computationally intensive.

```r
# Perform overall simulation to enable production of aggregate results
# It tallies the proportion of I2 which is non-zero, significant het and significant
# weird moderation at the per ManyLabs project level.
# It effectively finds the typical ML-2 results, given the parameters
# @param nML2: number of ManyLabs projects to simulate
# @param effects: number of different effects to include
# @param k: number of sites to include
# @param ml2.es: use original ML-2 effect sizes or not. Setting `ml2.es=T` will use the original
↪  ml2 effect sizes.
```

```r
# @param d.lo: minimum value of Cohen's d
# @param d.hi: maximum value of Cohen's d
# @param cultural.influence: level of cultural moderation
# @param pr.rep: pr.rep = 1 means that every site starts with a true effect (d = .35, before
↪  cultural.influence)
# @param culture: cultural influence adjustment or not. Setting `culture=F` will keep all
↪  samples from the same country the same in terms of true effect size.
# @param silent: whether to display notifications for the running of each simulation or not
sim_many_ml2 <- function(nML2, effects, k, ml2.es, d.lo, d.hi, cultural.influence, pr.rep,
↪  culture, silent) {

  # Run nML2 multiple ManyLabs2 simulations and store results
  bin <- data.frame() #
  for (j in 1:nML2) {
    if(silent == F) {
      print(paste0("Running ML-2 ", j, "/", nML2))
    }
    bin <- rbind(bin, sim_multi_ml2(effects = effects, k = k, ml2.es = ml2.es, d.lo = d.lo, d.hi
↪  = d.hi, cultural.influence = cultural.influence, pr.rep = pr.rep, culture = culture))
  }

  return(bin)

}
```

Here is the output setting `nML2 = 5`, keeping the other parameters the same as for the previous functions.

```r
ml2 <- sim_many_ml2(nML2 = 5, effects = 5, k = 10, ml2.es = T, d.lo = NULL, d.hi = NULL,
↪  cultural.influence = 2, pr.rep = 1, culture = T, silent = F)
```

```
## [1] "Running ML-2 1/5"
## [1] "Running ML-2 2/5"
## [1] "Running ML-2 3/5"
## [1] "Running ML-2 4/5"
## [1] "Running ML-2 5/5"
```

```r
ml2
```

```
##   lowest.d highest.d mean.d effects  k cultural.influence culture  tau.median
## 1    -0.07      1.75  0.716       5 10                  2    TRUE 0.406302130
## 2    -0.03      1.35  0.304       5 10                  2    TRUE 0.095665804
## 3    -0.03      0.86  0.156       5 10                  2    TRUE 0.001753034
## 4    -0.13      0.95  0.276       5 10                  2    TRUE 0.120538379
## 5    -0.03      1.18  0.480       5 10                  2    TRUE 0.163276651
##   pr.tau.over.10 pr.I2.nonZero pr.I2.over.50 pr.Q.sig.001 pr.mod.sig.004
## 1            0.6           0.6           1.0          0.6            0.0
## 2            0.4           0.2           0.6          0.2            0.2
## 3            0.4           0.2           0.4          0.2            0.0
## 4            0.6           0.2           0.8          0.2            0.0
## 5            0.6           0.4           0.8          0.4            0.4
```

It can also be used to produce overall summaries:

```r
summary(ml2)
```

```
##     lowest.d        highest.d         mean.d          effects         k
##  Min.   :-0.130   Min.   :0.860   Min.   :0.1560   Min.   :5   Min.   :10
##  1st Qu.:-0.070   1st Qu.:0.950   1st Qu.:0.2760   1st Qu.:5   1st Qu.:10
```

```
##  Median :-0.030   Median :1.180   Median :0.3040   Median :5   Median :10
##  Mean   :-0.058   Mean   :1.218   Mean   :0.3864   Mean   :5   Mean   :10
##  3rd Qu.:-0.030   3rd Qu.:1.350   3rd Qu.:0.4800   3rd Qu.:5   3rd Qu.:10
##  Max.   :-0.030   Max.   :1.750   Max.   :0.7160   Max.   :5   Max.   :10
##  cultural.influence culture          tau.median       pr.tau.over.10
##  Min.   :2          Mode:logical   Min.   :0.001753   Min.   :0.40
##  1st Qu.:2          TRUE:5         1st Qu.:0.095666   1st Qu.:0.40
##  Median :2                         Median :0.120538   Median :0.60
##  Mean   :2                         Mean   :0.157507   Mean   :0.52
##  3rd Qu.:2                         3rd Qu.:0.163277   3rd Qu.:0.60
##  Max.   :2                         Max.   :0.406302   Max.   :0.60
##  pr.I2.nonZero  pr.I2.over.50   pr.Q.sig.001  pr.mod.sig.004
##  Min.   :0.20   Min.   :0.40   Min.   :0.20   Min.   :0.00
##  1st Qu.:0.20   1st Qu.:0.60   1st Qu.:0.20   1st Qu.:0.00
##  Median :0.20   Median :0.80   Median :0.20   Median :0.00
##  Mean   :0.32   Mean   :0.72   Mean   :0.32   Mean   :0.12
##  3rd Qu.:0.40   3rd Qu.:0.80   3rd Qu.:0.40   3rd Qu.:0.20
##  Max.   :0.60   Max.   :1.00   Max.   :0.60   Max.   :0.40
```

We can also ask for specific outputs, as before. Here is the mean power to detect moderation, median power of Q, etc:

```
mean(ml2$pr.mod.sig.004)
```

```
## [1] 0.12
```

```
median(ml2$pr.Q.sig.001)
```

```
## [1] 0.2
```

```
mean(ml2$pr.tau.over.10)
```

```
## [1] 0.52
```

1.1.2 Simulation Software

The full simulation code can be found in the folder “simulation” and the files “sim_functions.R” and “run_sim.R”. The simulation was written in R 4.2.1 (R Core Team, 2022) with the packages tidyverse 1.3.2 (Wickham et al., 2019), metafor 3.8-1 (Viechtbauer, 2010), ggpubr 0.4.0 (Kassambara, 2020) and patchwork 1.1.2 (Pedersen, 2022).

1.2 ML2 - Reanalysis: Methods

1.2.1 Cultural Distance

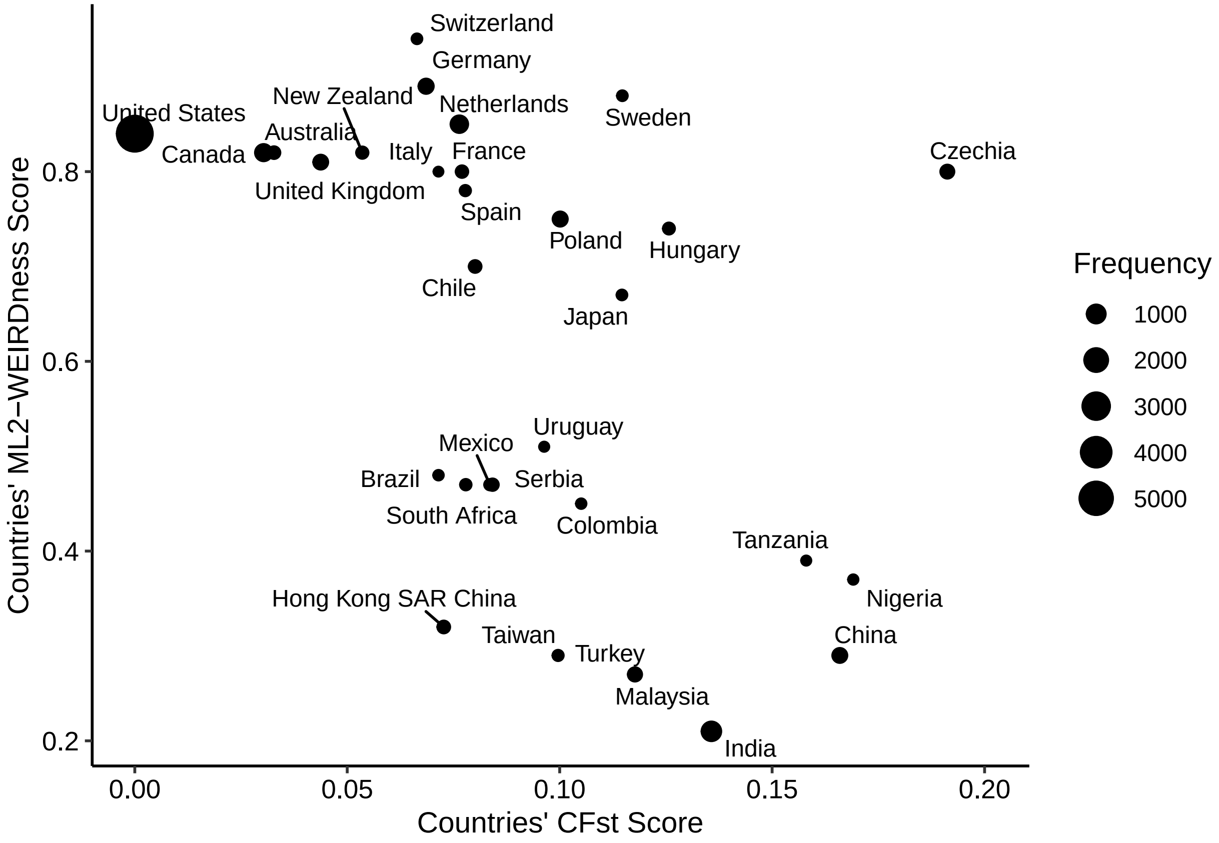

Supplementary Figure 1: Scatter plot for CF_{ST} values and ML2-WEIRDness scores. The plot reveals a moderate correlation between the CF_{ST} values and ML2-WEIRDness scores before the mean split, r = 0.49.

1.2.2 Analysis Plan

In Supplementary Figure 2 we show an overview of the pre-registered analysis plan.

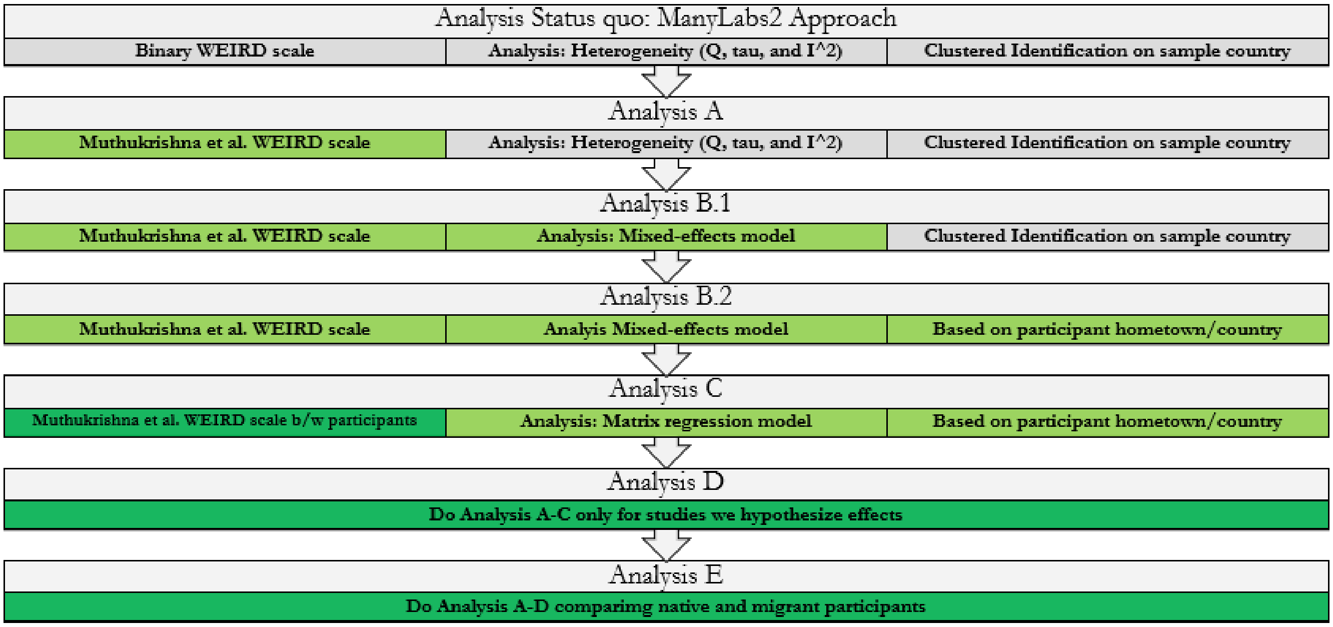

Supplementary Figure 2: Overview of the pre-registered analysis plan. The colors indicate the stepwise change in the conceptualization and operationalization of the measuring cultural differences. First, we used the analytic approaches of Klein et al. (gray). We then changed to cultural distance based on Muthukrishna et al. (2020) (light green), and finally used approaches based on Muthukrishna et al. (2020), but between participants (dark green).

Supplementary Table 2. Statistical analysis method to calculate the effect size for each study.

Study	Method	Formula
Cardinal direction and socioeconomic status (Huang, Tse, & Cho, 2014)	lm	mouse_position $\sim$ condition
Structure promotes goal pursuit (Kay et al., 2014)	lm	csv $\sim$ condition
Disfluency engages analytic processing (Alter et al., 2007)	lm	solved $\sim$ condition
Moral foundations of liberals versus conservatives (Graham, Haidt, & Nosek, 2009)	cor_fisherZ	binding $\sim$ politics
Affect and risk (Rottenstreich & Hsee, 2001)	glm	kiss_money $\sim$ condition
Consumerism undermines trust (Bauer et al., 2012)	lm	trust $\sim$ condition
Correspondence bias (Miyamoto & Kitayama, 2002)	lm	attitude $\sim$ constraint + condition
Disgust sensitivity predicts homophobia (Inbar et al., 2009)	cor_fisherZ	intentionality + dsr
Influence of incidental anchors on judgment (Critcher & Gilovich, 2008)	lm	sales_proportion $\sim$ condition
Social value orientation and family size (Van Lange et al., 1997)	cor_fisherZ	SVO $\sim$ siblings_total
Trolley Dilemma 1: principle of double effect (Hauser et al., 2007)	glm	morally_permission $\sim$ condition
Sociometric status and well-being (Anderson et al., 2012)	lm	subjective_well_being $\sim$ condition
False consensus: supermarket scenario (Ross, Greene, & House, 1977)	lm	peers $\sim$ you
False consensus: traffic-ticket scenario (Ross et al., 1977)	lm	peers $\sim$ you
Vertical position and power (Giessner & Schubert, 2007)	lm	dominance $\sim$ condition
Effect of framing on decision making (Tversky & Kahneman, 1981)	glm	agreement $\sim$ condition
Trolley Dilemma 2: principle of double effect (Hauser et al., 2007)	glm	morality_judgment $\sim$ condition
Reluctance to tempt fate (Risen & Gilovich, 2008)	lm	likelihood $\sim$ condition
Construing actions as choices (Savani et al., 2010)	glmer	choice $\sim$ importance * condition + (1 | ResponseID/variable)
Preferences for formal versus intuitive reasoning (Norenzayan et al., 2002)	lm	rule_based_choice $\sim$ condition
Less-is-better effect (Hsee, 1998)	lm	generosity $\sim$ condition
Moral typecasting (Gray & Wegner, 2009)	lm	responsibility $\sim$ condition
Moral violations and desire for cleansing (Zhong & Liljenquist, 2006)	lm	desirability $\sim$ condition
Assimilation and contrast effects in question sequences (Schwarz, Strack, & Mai, 1991)	cor_fisherZ	first + second
Effect of choosing versus rejectingon relative desirability (Shafir, 1993)	glm	choice $\sim$ condition
Priming "heat" increases belief in global warming (Zaval et al., 2014)	lm	belief $\sim$ condition
Perceived intentionality for side effects (Knobe, 2003)	lm	intentionality $\sim$ condition
Directionality and similarity (Tversky & Gati, 1978)	lmer	rating $\sim$ condition + (condition | ResponseID) + (condition | variable)

1.2.2.1 Analysis A *Does cultural distance measured by the Muthukrishna et al. cultural distance scale at a sample level explain variation in the outcomes of the studies?*

Analysis ManyLabs2

```
rma(effect_sample= q, vi = vi, mods = ~ ML2_weird)
```

Analysis A

```
rma(effect_sample= q, vi = vi, mods = ~ US_CFst_ sample)

rma(effect_sample= q, vi = vi, mods = ~ US_imputed_CFst_ sample)
```

1.2.2.2 Analysis B *Does cultural variation identified on the individual level by the Muthukrishna et al. WEIRD scale explain variation in the behavior of participants?*

Analysis B.1 Clustered

Effect_clustered_origin ~ US_CFst_source_country + [Controls][1]

```
Effect_clustered_origin ~ US_imputed_CFst_source_country + [Controls]
```

Analysis B.2 Unclustered

```
Effect_individual ~ US_CFst_birth_country_filtered + [Controls]

Effect_individual ~ US_imputed_CFst_birth_country_filtered + [Controls]

Effect_individual ~ US_CFst_birth_country_imputed + [Controls]

Effect_individual ~ US_imputed_CFst_birth_country_imputed + [Controls]

Effect_individual ~ US_CFst_hometown + [Controls]

Effect_individual ~ US_imputed_CFst_hometown + [Controls]
```

1.2.2.3 Analysis C *Does the cultural distance between participants at an individual level explain variation in the behavior of participants?*

For the matrix regression we used the `multi.mantel` function from the R package phytools 1.2-0 (Revell, 2012). Therefore, matching the variants of Analysis B we ran 8 different Analysis C variants.

Analysis C B.1 Clustered

```
multi.mantel(Y = Effect_clustered_origin, X = US_CFst_source_country, nperm=1000)

multi.mantel(Y = Effect_clustered_origin, X = US_imputed_CFst_source_country, nperm=1000)
```

Analysis C B.2 Unclustered

```
multi.mantel(Y = Effect_individual, X = US_CFst_birth_country_filtered, nperm=1000)

multi.mantel(Y = Effect_individual, X = US_imputed_CFst_birth_country_filtered, nperm=1000)

multi.mantel(Y = Effect_individual, X = US_CFst_birth_country_imputed, nperm=1000)

multi.mantel(Y = Effect_individual, X = US_imputed_CFst_birth_country_imputed, nperm=1000)

multi.mantel(Y = Effect_individual, X = US_CFst_hometown, nperm=1000)

multi.mantel(Y = Effect_individual, X = US_imputed_CFst_hometown, nperm=1000)
```

[1] The included control variables refer to the variables controlled for in the ML-2 study.

1.2.2.4 Analysis D *For each study, we have stated whether we expect culture to matter or not. We ran our analyses on this subset of culturally relevant studies as well as the full set of studies.*

Our hypotheses are subjective. To properly develop hypotheses for all study would require carefully considering each paper and linking it to the existing body of theory or developing new theory (Muthukrishna & Henrich, 2019). Since this is not feasible in the current scope of the re-analysis, we instead use subjective criteria as to suspect the replication of studies:

- Cultural variation can only be predicted when the effect is a real effect. The study should replicate in the population where it was initially reported (or, the closest approximation of that population). However, if the 'effect' is not a real effect in that it does not replicate there, we can't expect population-level variation. We argue that this would exclude many studies, though in some cases it is difficult to judge based on the reported results.
- Studies which did not replicate in ML2 are only included in our analysis for the sake of completeness. For those that did replicate in ML2, we differentiate based on how much prior theoretical evidence exists (none, 1 theory, theory + evidence), based on our knowledge. For criteria that are 'maybe', we will include one analysis including these as an exploratory analysis.

Supplementary Table 3. Which studies we hypothesize could vary culturally. The background of table cells are colored to indicate hypothesized effects and whether studies replicated or not. Studies that failed to replicate in ML2 are colored in gray and studies that replicated in ML2 are colored in pale green to indicate that this is an exploratory analysis, with no predictions for cross-cultural variation. If there is at least one existing theory indicating non-random cultural variation this is a middle ground, with a mid number of studies and is colored in mid green. When there is existing theoretical and empirical evidence indicating non-random cultural variation this strictest analysis is indicated in the darkest green. Studies where WEIRD moderation was found in ML2 are colored in blue. * is used to indicate studies where a different cultural difference measure were used compared to that used in the original study.

Study	Successful Replication in ML-2	Theoretical Evidence for non-random cultural variation	Theoretical Evidence & Empirical Evidence	WEIRD Moderation found in ML2
Huang, Tse, & Cho (2014)	Yes*	No	No	Yes
Kay, Laurin, Fitzsimons, & Landau (2014)	No	No	No	No
Alter, Oppenheimer, Epley, & Eyre (2007)	No	No	No	No
Graham, Haidt, & Nosek (2009)	Yes	Maybe	No	No
Rottenstreich & Hsee (2001)	No	No	No	No
Bauer, Wilkie, Kim, & Bodenhausen (2012)	Yes	Maybe	No	No
Miyamoto & Kitayama (2002)	Yes*	Yes	Yes	No
Inbar, Pizarro, Knobe, & Bloom (2009)	No	No	No	No
Critcher & Gilovich (2008)	No	No	No	No
Van Lange, Otten, De Bruin, & Joireman (1997)	No effect size est.	No	No	No
Hauser, Cushman, Young, Jin, & Mikhail (2007)	Yes	Yes	Maybe	No
Anderson, Kraus, Galinsky, & Keltner (2012)	No	No	No	No
Ross, Greene, & House (1977)	Yes	Maybe	No	No
Ross et al. (1977)	Yes	Maybe	No	No
Giessner & Schubert (2007)	No	No	No	No
Tversky & Kahneman (1981)	Yes	No	No	No
Hauser et al. (2007)	Yes	Yes	Yes	No
Risen & Gilovich (2008)	Yes	Yes	No	No
Savani, Markus, Naidu, Kumar, & Berlia (2010)	No*	No	No	No
Norenzayan, Smith, Kim, & Nisbett (2002)	Yes*	Yes	Yes	Yes
Hsee (1998)	Yes	No	No	No
Gray & Wegner (2009)	Yes	Maybe	Maybe	No
Zhong & Liljenquist (2006)	No	No	No	No
Schwarz, Strack, & Mai (1991)	No	Maybe	No	No
Shafir (1993)	No	No	No	No
Zaval, Keenan, Johnson, & Weber (2014)	No	No	No	No
Knobe (2003)	Yes	Yes	Yes	Yes
Tversky & Gati (1978)	No	No	No	No

1.2.2.5 Analysis E *How similar is the behavior of participants in their native country to participants not in their native country (i.e. how different are migrant populations in their country of origin)?*

For comparability, we only ran Analysis E for studies where we used either linear regression (`lm`) or logistic regression (`glm`) as the statistical analysis method. This excluded five studies from analysis. Four studies were excluded as they had different experimental designs meaning that a regression model was inappropriate for re-analysis: Graham et al. (2009), Inbar et al. (2009), Schwarz et al. (1991), and Van Lange et al. (1997), and one study was excluded for the model failing to converge: Savani et al. (2010).

As stated in our preregistration we ran three versions of Analysis E using the three methods of determining a participant's country of origin: by their birth country excluding participants missing birth countries and hometowns, by their birth country, where participants missing birth countries are assumed to be native to the sample country and by their hometown.

1.2.3 Reanalysis Software

The full code used for reanalysis can be found in the folder "scripts". The reanalysis was written in R 4.2.1 (R Core Team, 2022). The packages tidyverse 1.3.2 (Wickham et al., 2019), data.table 1.14.2 (Dowle & Srinivasan, 2022), countrycode 1.4.0 (Arel-Bundock et al., 2018), rnaturalearth 0.1.0 (South, 2017), sf 1.0-8 (Pebesma, 2018), stringi 1.7.8 (Gagolewski, 2022), maps 3.4.0 (Brownrigg et al., 2021), plyr 1.8.8 (Wickham, 2011b), rio 0.5.29 (Chan et al., 2021), reshape2 1.4.4 (Wickham, 2007), devtools 2.4.4 (Wickham et al., 2022) and gdata 2.18.0 (Warnes et al., 2022) were used for data cleaning. The following packages were used for statistical analysis: lme4 1.1-30 (Bates et al., 2015), lmerTest 3.1-3 (Kuznetsova et al., 2017), metafor 3.8-1 (Viechtbauer, 2010), ecodist 2.0.9 (Goslee & Urban, 2007), phytools 1.2-0 (Revell, 2012). Additionally the `cor_test_fisherZ` from the original analysis was used for performing Fisher's r-to-z test (https://raw.githubusercontent.com/ManyLabsOpenScience/manylabRs/master/R/manylabRs_SOURCE.R). The package testthat 3.1.4 (Wickham, 2011a) was used for unit tests. For data visualization the tidyverse 1.3.2 (Wickham et al., 2019), patchwork 1.1.2 (Pedersen, 2022), ggforestplot 0.1.0 (Scheinin et al., 2022) and ggrepel 0.9.1 (Slowikowski, 2021) packages were used.

2 Supplementary Results

2.1 Additional Simulation Results

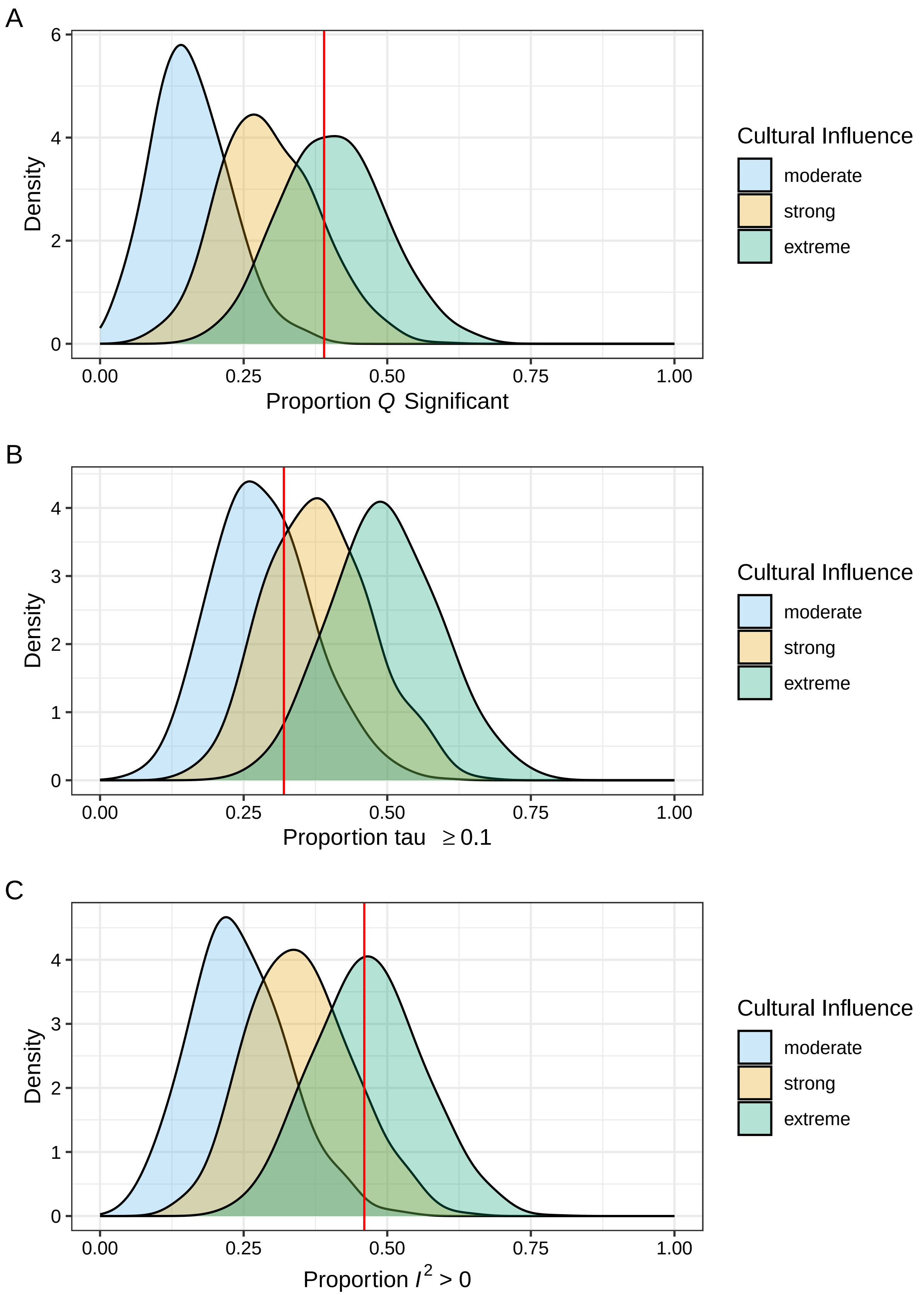

Supplementary Figure 3: Comparing the share of studies replicated in ML2 with significant heterogeneity to results from simulations that induce moderate, strong, and extreme levels of heterogeneity. A shows the proportion of Q test effects that were significant at the 0.001 level, B shows the proportion of effects that were significant at the tau >= 0.1 level, and C shows the proportion of effects with $I^2 > 0$. The red lines indicate the observed results in ML2. The results suggest that the pattern of results from ML2 are most consistent with strong levels of heterogeneity, of which cultural heterogeneity is one, but not the only possible source of heterogeneity (e.g., variation in studies being administered online or in the lab could also play a role).

2.2 Description of ML-2 Sample

2.2.1 Overview of Distribution of Birth Countries

Supplementary Table 4. Frequency of participants per birth country in Slate 1.

Birth Country	*N*
United States	2040
China	574
Netherlands	458
India	434
Canada	335
Turkey	239
Poland	231
Hungary	170
Germany	159
Mexico	156
Hong Kong SAR China	155
Chile	153
United Kingdom	148
Czechia	131
Taiwan	126
Japan	123
Sweden	108
Belgium	105
Brazil	105
Costa Rica	102
Serbia	101
Switzerland	100
Uruguay	81
South Africa	75
Austria	74
New Zealand	69
France	50
South Korea	47
United Arab Emirates	44
Spain	39
Pakistan	36
Portugal	35
Philippines	31
Sri Lanka	20
Saudi Arabia	14
Ukraine	13
Singapore	12
Other	11
Australia	10
Egypt	10
Jordan	10
Albania	8
Bangladesh	8
Slovakia	8
Vietnam	8
Colombia	7

Supplementary Table 4. Frequency of participants per birth country in Slate 1. *(continued)*

Birth Country	*N*
Iran	7
Norway	7
Afghanistan	6
Ecuador	6
Ghana	6
Israel	6
Jamaica	6
Syria	6
Thailand	6
Venezuela	6
Armenia	5
Bulgaria	5
Montenegro	5
Argentina	4
Bosnia & Herzegovina	4
Dominican Republic	4
Finland	4
Iraq	4
Italy	4
Malaysia	4
Romania	4
Russia	4
Angola	3
Belarus	3
Bolivia	3
Haiti	3
Indonesia	3
Kazakhstan	3
Kenya	3
Nepal	3
Nigeria	3
Zimbabwe	3
Congo - Brazzaville	2
Estonia	2
Greece	2
Guyana	2
Libya	2
Oman	2
Panama	2
Somalia	2
Suriname	2
Trinidad & Tobago	2
Algeria	1
Bahamas	1
Brunei	1
Cambodia	1
Croatia	1
Cuba	1
Cyprus	1

Supplementary Table 4. Frequency of participants per birth country in Slate 1. *(continued)*

Birth Country	*N*
Côte d'Ivoire	1
Denmark	1
Fiji	1
Guatemala	1
Guinea	1
Honduras	1
Iceland	1
Ireland	1
Kuwait	1
Lebanon	1
Liberia	1
Madagascar	1
Mauritius	1
Micronesia (Federated States of)	1
Morocco	1
Myanmar (Burma)	1
Namibia	1
Nicaragua	1
North Korea	1
Palau	1
Peru	1
Samoa	1
Senegal	1
Seychelles	1
Sierra Leone	1
Slovenia	1
St. Vincent & Grenadines	1
Sudan	1
Tanzania	1
Uganda	1
Uzbekistan	1

Supplementary Table 5. Frequency of participants per birth country in Slate 2.

Birth Country	*N*
United States	2830
India	825
Germany	428
Netherlands	271
Poland	253
United Kingdom	244
Costa Rica	190
France	189
Czechia	185
Australia	181
Canada	142
China	138
Turkey	125

Supplementary Table 5. Frequency of participants per birth country in Slate 2. *(continued)*

Birth Country	*N*
Malaysia	114
Colombia	105
Nigeria	105
Italy	102
Belgium	94
Serbia	93
Spain	87
New Zealand	86
Hong Kong SAR China	85
South Africa	83
Chile	80
Austria	78
United Arab Emirates	74
Philippines	40
Tanzania	37
Russia	33
Saudi Arabia	31
South Korea	30
Pakistan	26
Egypt	21
Japan	20
Other	20
Mexico	19
Ukraine	17
Brazil	16
Slovakia	16
Jordan	15
Singapore	15
Vietnam	14
Greece	11
Lebanon	11
Finland	10
Romania	10
Iran	9
Kenya	9
Uganda	9
Bulgaria	8
Indonesia	8
Israel	8
Syria	8
Zimbabwe	8
Belarus	7
Bosnia & Herzegovina	7
Croatia	7
Ecuador	7
Iraq	7
Ireland	7
Afghanistan	6
Albania	6

Supplementary Table 5. Frequency of participants per birth country in Slate 2. *(continued)*

Birth Country	*N*
Bangladesh	6
Hungary	6
Kazakhstan	6
Algeria	5
Argentina	5
Cameroon	5
Dominican Republic	5
Ethiopia	5
Ghana	5
Kuwait	5
Montenegro	5
Morocco	5
Sweden	5
Switzerland	5
Venezuela	5
Guatemala	4
Lithuania	4
Norway	4
Panama	4
Sri Lanka	4
Tajikistan	4
Thailand	4
Azerbaijan	3
Cyprus	3
Luxembourg	3
Namibia	3
Peru	3
Portugal	3
Senegal	3
Slovenia	3
Angola	2
Antigua & Barbuda	2
Bahrain	2
Botswana	2
Denmark	2
Georgia	2
Haiti	2
Iceland	2
Jamaica	2
Latvia	2
Libya	2
Malawi	2
Mongolia	2
Nepal	2
Nicaragua	2
Oman	2
Qatar	2
Samoa	2
Somalia	2

Supplementary Table 5. Frequency of participants per birth country in Slate 2. *(continued)*

Birth Country	*N*
Trinidad & Tobago	2
Andorra	1
Armenia	1
Bahamas	1
Benin	1
Brunei	1
Burundi	1
Cambodia	1
Cape Verde	1
Cuba	1
Djibouti	1
Eritrea	1
Estonia	1
Guinea	1
Guyana	1
Kyrgyzstan	1
Liberia	1
Madagascar	1
Mauritius	1
Nauru	1
North Korea	1
North Macedonia	1
Togo	1
Tonga	1
Turkmenistan	1
Uruguay	1
Yemen	1

2.2.2 Movement From Birth Countries to Sample Countries

Supplementary Table 6. Constitution of sample countries by birth countries as indicated by participants for birth countries with less than 5 participants. This table is in complement to Figure 2, which shows the constitution of sample countries by birth countries as indicated by participants for birth countries 5 or more participants.

Birth Country	Source Country	Frequency
Bangladesh	United States	5
Croatia	United States	5
Greece	United States	5
Haiti	United States	5
Iran	United States	5
Ireland	United States	5
Jamaica	United States	5
Jordan	United States	5
Lebanon	United States	5
Portugal	United States	5
Sweden	United States	5
Argentina	United States	4
Austria	United States	4
Belgium	United States	4
Ethiopia	United States	4
Guatemala	United States	4
Hungary	United States	4
Malaysia	United States	4
Norway	United States	4
Panama	United States	4
Senegal	United States	4
Serbia	United States	4
South Africa	United States	4
Tanzania	United States	4
Angola	United States	3
Azerbaijan	United States	3
Cameroon	United States	3
Czechia	United States	3
Egypt	United States	3
Lithuania	United States	3
Slovenia	United States	3
Trinidad & Tobago	United States	3
Algeria	United States	2
Antigua & Barbuda	United States	2
Bahamas	United States	2
Bolivia	United States	2
Bosnia & Herzegovina	United States	2
Georgia	United States	2
Iraq	United States	2
Latvia	United States	2
Liberia	United States	2
Mongolia	United States	2
Nepal	United States	2
Peru	United States	2
Samoa	United States	2
Spain	United States	2
Switzerland	United States	2
Syria	United States	2
Uganda	United States	2
United Arab Emirates	United States	2
Armenia	United States	1
Benin	United States	1
Cape Verde	United States	1
Chile	United States	1
Congo - Brazzaville	United States	1

Supplementary Table 6. Constitution of sample countries by birth countries as indicated by participants for birth countries with less than 5 participants. This table is in complement to Figure 2, which shows the constitution of sample countries by birth countries as indicated by participants for birth countries 5 or more participants. *(continued)*

Birth Country	Source Country	Frequency
Costa Rica	United States	1
Cuba	United States	1
Cyprus	United States	1
Côte d'Ivoire	United States	1
Denmark	United States	1
Eritrea	United States	1
Guinea	United States	1
Iceland	United States	1
Kazakhstan	United States	1
Luxembourg	United States	1
Madagascar	United States	1
Micronesia (Federated States of)	United States	1
Myanmar (Burma)	United States	1
Namibia	United States	1
Nicaragua	United States	1
North Korea	United States	1
North Macedonia	United States	1
Oman	United States	1
Slovakia	United States	1
St. Vincent & Grenadines	United States	1
Togo	United States	1
Uruguay	United States	1
Yemen	United States	1
Bulgaria	United Kingdom	5
Malaysia	United Kingdom	5
Norway	United Kingdom	5
United Arab Emirates	Canada	5
Bangladesh	Canada	4
Japan	Canada	4
Malaysia	Canada	4
Russia	United Kingdom	4
Saudi Arabia	Canada	4
Singapore	United Kingdom	4
Zimbabwe	United Kingdom	4
Afghanistan	Canada	3
Cyprus	United Kingdom	3
France	United Kingdom	3
Germany	Canada	3
Guyana	Canada	3
Italy	United Kingdom	3
Mexico	Canada	3
Netherlands	United Kingdom	3
Russia	Canada	3
Singapore	Canada	3
Spain	United Kingdom	3
Australia	United Kingdom	2
Belarus	United Kingdom	2
Canada	United Kingdom	2
Colombia	Canada	2
Egypt	Canada	2
Estonia	United Kingdom	2
Ghana	Canada	2
Greece	United Kingdom	2
Iraq	Canada	2
Israel	Canada	2
Jamaica	United Kingdom	2

Supplementary Table 6. Constitution of sample countries by birth countries as indicated by participants for birth countries with less than 5 participants. This table is in complement to Figure 2, which shows the constitution of sample countries by birth countries as indicated by participants for birth countries 5 or more participants. *(continued)*

Birth Country	Source Country	Frequency
Kenya	United Kingdom	2
Nepal	United Kingdom	2
Saudi Arabia	United Kingdom	2
Slovakia	United Kingdom	2
Somalia	Canada	2
Somalia	United Kingdom	2
South Africa	Canada	2
South Africa	United Kingdom	2
South Korea	United Kingdom	2
Sri Lanka	United Kingdom	2
Syria	Canada	2
Vietnam	United Kingdom	2
Albania	United Kingdom	1
Algeria	Canada	1
Argentina	Canada	1
Belarus	Canada	1
Belgium	United Kingdom	1
Bosnia & Herzegovina	Canada	1
Brazil	United Kingdom	1
Cambodia	United Kingdom	1
Denmark	United Kingdom	1
Ecuador	Canada	1
Estonia	Canada	1
Finland	United Kingdom	1
France	Canada	1
Iraq	United Kingdom	1
Ireland	United Kingdom	1
Italy	Canada	1
Jamaica	Canada	1
Jordan	Canada	1
Kazakhstan	Canada	1
Kazakhstan	United Kingdom	1
Kuwait	Canada	1
Lebanon	United Kingdom	1
Mauritius	Canada	1
Nepal	Canada	1
Netherlands	Canada	1
Nigeria	United Kingdom	1
Oman	Canada	1
Oman	United Kingdom	1
Peru	United Kingdom	1
Philippines	United Kingdom	1
Romania	Canada	1
Romania	United Kingdom	1
Serbia	Canada	1
Switzerland	Canada	1
Thailand	Canada	1
Trinidad & Tobago	Canada	1
Turkey	Canada	1
Turkey	United Kingdom	1
Turkmenistan	Canada	1
Ukraine	United Kingdom	1
Vietnam	Canada	1
China	Netherlands	4
Armenia	Netherlands	3
Bosnia & Herzegovina	Netherlands	3
Afghanistan	Netherlands	2

Supplementary Table 6. Constitution of sample countries by birth countries as indicated by participants for birth countries with less than 5 participants. This table is in complement to Figure 2, which shows the constitution of sample countries by birth countries as indicated by participants for birth countries 5 or more participants. *(continued)*

Birth Country	Source Country	Frequency
Australia	Netherlands	2
Iran	Netherlands	2
Spain	Netherlands	2
Suriname	Netherlands	2
Switzerland	Netherlands	2
United Kingdom	Netherlands	2
Albania	Netherlands	1
Bangladesh	Netherlands	1
Belgium	Netherlands	1
Brunei	Netherlands	1
Colombia	Netherlands	1
Dominican Republic	Netherlands	1
Ethiopia	Netherlands	1
Ghana	Netherlands	1
Guatemala	Netherlands	1
Hungary	Netherlands	1
Indonesia	Netherlands	1
Iraq	Netherlands	1
Ireland	Netherlands	1
Lithuania	Netherlands	1
North Korea	Netherlands	1
Poland	Netherlands	1
Russia	Netherlands	1
United Arab Emirates	Netherlands	1
Zimbabwe	Netherlands	1
Australia	New Zealand	5
Singapore	Australia	5
United States	New Zealand	5
Brazil	Australia	4
South Africa	New Zealand	4
United Kingdom	Australia	4
Canada	New Zealand	3
India	Australia	3
Philippines	Australia	3
France	Australia	2
India	New Zealand	2
Philippines	New Zealand	2
Singapore	New Zealand	2
South Korea	Australia	2
United States	Australia	2
Austria	New Zealand	1
Brunei	Australia	1
China	New Zealand	1
Fiji	New Zealand	1
Ghana	Australia	1
Hong Kong SAR China	New Zealand	1
Indonesia	Australia	1
Iran	Australia	1
Iraq	Australia	1
Iraq	New Zealand	1
Ireland	New Zealand	1
Israel	New Zealand	1
Japan	Australia	1
Jordan	Australia	1
Lebanon	Australia	1
Malaysia	New Zealand	1
Norway	Australia	1

Supplementary Table 6. Constitution of sample countries by birth countries as indicated by participants for birth countries with less than 5 participants. This table is in complement to Figure 2, which shows the constitution of sample countries by birth countries as indicated by participants for birth countries 5 or more participants. *(continued)*

Birth Country	Source Country	Frequency
Samoa	New Zealand	1
Saudi Arabia	Australia	1
Serbia	Australia	1
Seychelles	New Zealand	1
South Korea	New Zealand	1
Sri Lanka	Australia	1
Sri Lanka	New Zealand	1
Sudan	New Zealand	1
Sweden	Australia	1
Switzerland	New Zealand	1
Vietnam	Australia	1
Zimbabwe	New Zealand	1
Poland	Germany	5
Kazakhstan	Germany	4
Russia	Germany	4
Greece	Germany	3
Luxembourg	Germany	2
Switzerland	Germany	2
Australia	Germany	1
Belgium	Germany	1
Bulgaria	Germany	1
Burundi	Germany	1
Finland	Germany	1
France	Germany	1
Iraq	Germany	1
Israel	Germany	1
Kyrgyzstan	Germany	1
Netherlands	Germany	1
Romania	Germany	1
Singapore	Germany	1
South Africa	Germany	1
Turkey	Germany	1
United States	Germany	1
Kuwait	United Arab Emirates	5
Lebanon	United Arab Emirates	5
Libya	United Arab Emirates	4
Bangladesh	United Arab Emirates	3
United Kingdom	United Arab Emirates	3
Algeria	United Arab Emirates	2
Canada	United Arab Emirates	2
Iran	United Arab Emirates	2
Iraq	United Arab Emirates	2
Qatar	United Arab Emirates	2
Ukraine	United Arab Emirates	2
Australia	United Arab Emirates	1
Bahrain	United Arab Emirates	1
Cameroon	United Arab Emirates	1
China	United Arab Emirates	1
Djibouti	United Arab Emirates	1
Kenya	United Arab Emirates	1
Oman	United Arab Emirates	1
Russia	United Arab Emirates	1
South Korea	United Arab Emirates	1
France	Switzerland	3
United States	Switzerland	2
Belgium	Switzerland	1

Supplementary Table 6. Constitution of sample countries by birth countries as indicated by participants for birth countries with less than 5 participants. This table is in complement to Figure 2, which shows the constitution of sample countries by birth countries as indicated by participants for birth countries 5 or more participants. *(continued)*

Birth Country	Source Country	Frequency
Bolivia	Switzerland	1
Brazil	Switzerland	1
Canada	Switzerland	1
Colombia	Switzerland	1
Germany	Switzerland	1
Greece	Switzerland	1
Portugal	Switzerland	1
Romania	Switzerland	1
Sweden	Switzerland	1
United Kingdom	Switzerland	1
Venezuela	Switzerland	1
Singapore	Spain	5
Tajikistan	Tanzania	4
Ecuador	Spain	3
Kenya	Tanzania	3
Australia	Tanzania	2
Italy	Spain	2
Malawi	Tanzania	2
Mexico	Spain	2
Algeria	Tanzania	1
Angola	Tanzania	1
Argentina	Spain	1
Armenia	Tanzania	1
Botswana	Tanzania	1
China	Spain	1
Costa Rica	Spain	1
Egypt	Tanzania	1
Germany	Spain	1
Namibia	Tanzania	1
Nauru	Tanzania	1
Panama	Spain	1
Peru	Spain	1
Romania	Spain	1
South Korea	Spain	1
Thailand	Tanzania	1
Tonga	Tanzania	1
United States	Spain	1
Saudi Arabia	India	4
Venezuela	Costa Rica	3
Argentina	Costa Rica	2
Australia	India	2
Belgium	India	2
Colombia	Costa Rica	2
Iceland	India	2
Nicaragua	Costa Rica	2
Albania	Costa Rica	1
Albania	India	1
Andorra	India	1
Angola	Costa Rica	1
Cameroon	India	1
Cuba	Costa Rica	1
Ecuador	Costa Rica	1
France	Costa Rica	1
Guinea	India	1
Honduras	India	1
Indonesia	India	1
Mexico	Costa Rica	1

Supplementary Table 6. Constitution of sample countries by birth countries as indicated by participants for birth countries with less than 5 participants. This table is in complement to Figure 2, which shows the constitution of sample countries by birth countries as indicated by participants for birth countries 5 or more participants. *(continued)*

Birth Country	Source Country	Frequency
Morocco	India	1
Spain	Costa Rica	1
United Kingdom	Costa Rica	1
United Kingdom	India	1
Bosnia & Herzegovina	Serbia	5
Russia	Czechia	4
Ukraine	Czechia	4
Croatia	Serbia	3
Morocco	Belgium	3
Netherlands	Belgium	3
Dominican Republic	Austria	2
Germany	Belgium	2
Hungary	Austria	2
Italy	Austria	2
Italy	Belgium	2
United States	Austria	2
United States	Serbia	2
Afghanistan	Poland	1
Austria	Serbia	1
Bahrain	Poland	1
Belarus	Poland	1
Bulgaria	Belgium	1
Canada	Czechia	1
France	Belgium	1
Germany	Poland	1
Greece	Poland	1
Greece	Serbia	1
Israel	Austria	1
Kazakhstan	Belgium	1
Kazakhstan	Czechia	1
Mauritius	Serbia	1
Namibia	Poland	1
Netherlands	Serbia	1
Palau	Austria	1
Poland	Czechia	1
Russia	Austria	1
Russia	Serbia	1
Sierra Leone	Belgium	1
Slovenia	Austria	1
Spain	Poland	1
Syria	Czechia	1
United States	Czechia	1
Uzbekistan	Poland	1
Vietnam	Czechia	1
Zimbabwe	South Africa	5
Austria	South Africa	1
Botswana	South Africa	1
Brazil	South Africa	1
Congo - Brazzaville	South Africa	1
Namibia	South Africa	1
Nigeria	South Africa	1
Bangladesh	Malaysia	1
India	Malaysia	1
Indonesia	Malaysia	1
Israel	Malaysia	1
Singapore	Malaysia	1
South Korea	Malaysia	1

Supplementary Table 6. Constitution of sample countries by birth countries as indicated by participants for birth countries with less than 5 participants. This table is in complement to Figure 2, which shows the constitution of sample countries by birth countries as indicated by participants for birth countries 5 or more participants. *(continued)*

Birth Country	Source Country	Frequency
Canada	Hong Kong SAR China	3
China	Taiwan	3
United States	Hong Kong SAR China	2
Argentina	Uruguay	1
Armenia	Taiwan	1
Brazil	Uruguay	1
Germany	Uruguay	1
Hong Kong SAR China	Taiwan	1
Italy	Uruguay	1
Madagascar	Taiwan	1
Malaysia	Hong Kong SAR China	1
Malaysia	Taiwan	1
Other	Hong Kong SAR China	1
Taiwan	Hong Kong SAR China	1
Venezuela	Uruguay	1
Other	Hungary	5
Slovakia	Hungary	5
Romania	Hungary	2
Serbia	Hungary	2
Denmark	Sweden	1
Finland	Sweden	1
Norway	Sweden	1
Sri Lanka	Sweden	1
Australia	China	3
South Korea	Japan	2
United States	Chile	2
Cambodia	China	1
Costa Rica	Chile	1
Hong Kong SAR China	China	1
Israel	Chile	1
Malaysia	Japan	1
United States	Japan	1
France	Portugal	2
Morocco	France	2
Other	Portugal	1
Panama	Colombia	1
Switzerland	France	1
United States	Colombia	1
Germany	Turkey	2
Romania	Italy	1
Uruguay	Mexico	1

2.2.3 Migrant populations in ML2

Supplementary Table 7. Share of migrant population in slate 1. Migrants are people who indicate a different birth country than the country they were sampled.

Country Site	Overall *N*	Migrants *N*	Migrants (%)
Austria	123	52	42.28
Belgium	110	8	7.27
Canada	593	266	44.86
Chile	155	2	1.29
China	383	3	0.78
Costa Rica	101	2	1.98
Czechia	140	10	7.14
France	44	2	4.55
Germany	91	3	3.30
Hong Kong SAR China	173	51	29.48
Hungary	183	14	7.65
India	360	9	2.50
Japan	113	4	3.54
Mexico	143	1	0.70
Netherlands	482	29	6.02
New Zealand	102	34	33.33
Poland	230	6	2.61
Portugal	35	3	8.57
Serbia	107	11	10.28
South Africa	74	4	5.41
Spain	54	17	31.48
Sweden	110	4	3.64
Switzerland	113	17	15.04
Taiwan	132	7	5.30
Turkey	240	6	2.50
United Arab Emirates	92	52	56.52
United Kingdom	142	25	17.61
United States	2361	352	14.91
Uruguay	85	5	5.88

Supplementary Table 8. Share of migrant population in slate 2. Migrants are people who indicate a different birth country than the country they were sampled.

Country Site	Overall *N*	Migrants *N*	Migrants (%)
Australia	234	82	35.04
Austria	103	40	38.83
Belgium	101	14	13.86
Canada	121	32	26.45
Chile	81	2	2.47
China	47	2	4.26
Colombia	102	2	1.96
Costa Rica	205	15	7.32
Czechia	203	20	9.85
France	177	7	3.95
Germany	380	43	11.32
Hong Kong SAR China	67	12	17.91
India	744	18	2.42
Italy	79	1	1.27
Malaysia	90	6	6.67
Netherlands	282	27	9.57
New Zealand	100	26	26.00
Poland	230	9	3.91
Serbia	104	14	13.46
South Africa	76	7	9.21
Spain	84	4	4.76
Tanzania	62	28	45.16
Turkey	125	6	4.80
United Arab Emirates	179	109	60.89
United Kingdom	291	121	41.58
United States	3556	760	21.37

2.2.4 CF_{ST} Scores for Slate 2

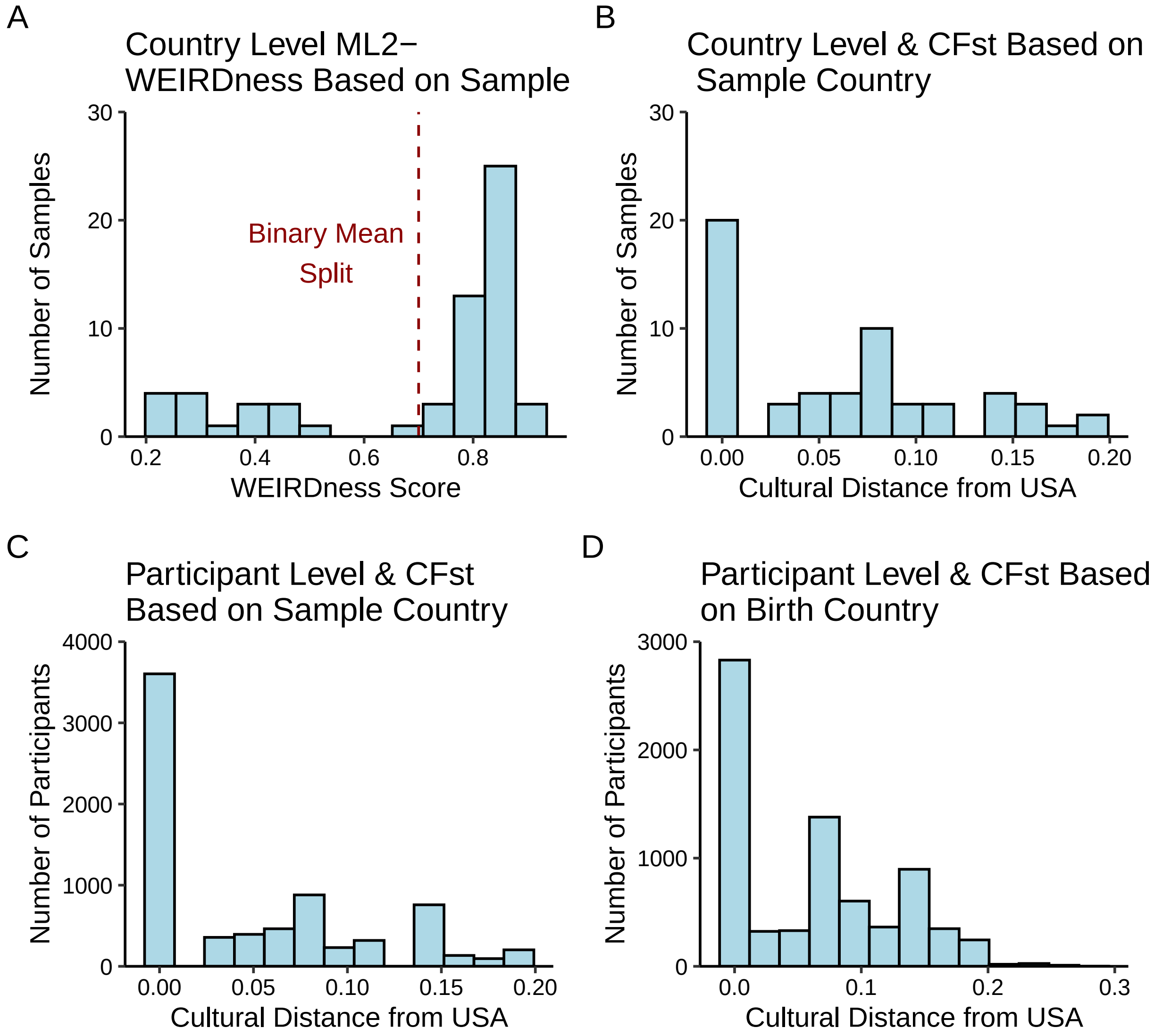

Supplementary Figure 4: Frequency distribution for different measures of cultural differences. The 3a) shows distribution of ML2-WEIRDness score in the Many Labs study (data retrieved from Klein et al. (2018) with cutoff for binary mean split at 0.7) on the sample level, based on the source country. 3b) shows the distribution of CF_{ST} values (Muthukrishna et al., 2020) on the sample level, based on the source country. 3c) shows CF_{ST} distribution on the participant level, based on the source country, and 3d) shows the CF_{ST} values on the participant level, based on the birth country of participants. The histograms show the data for Slate 2. The slate 1 histograms are presented in Figure 3.

2.3 ML2 - Reanalysis: Full Results

2.3.1 Analysis A Results

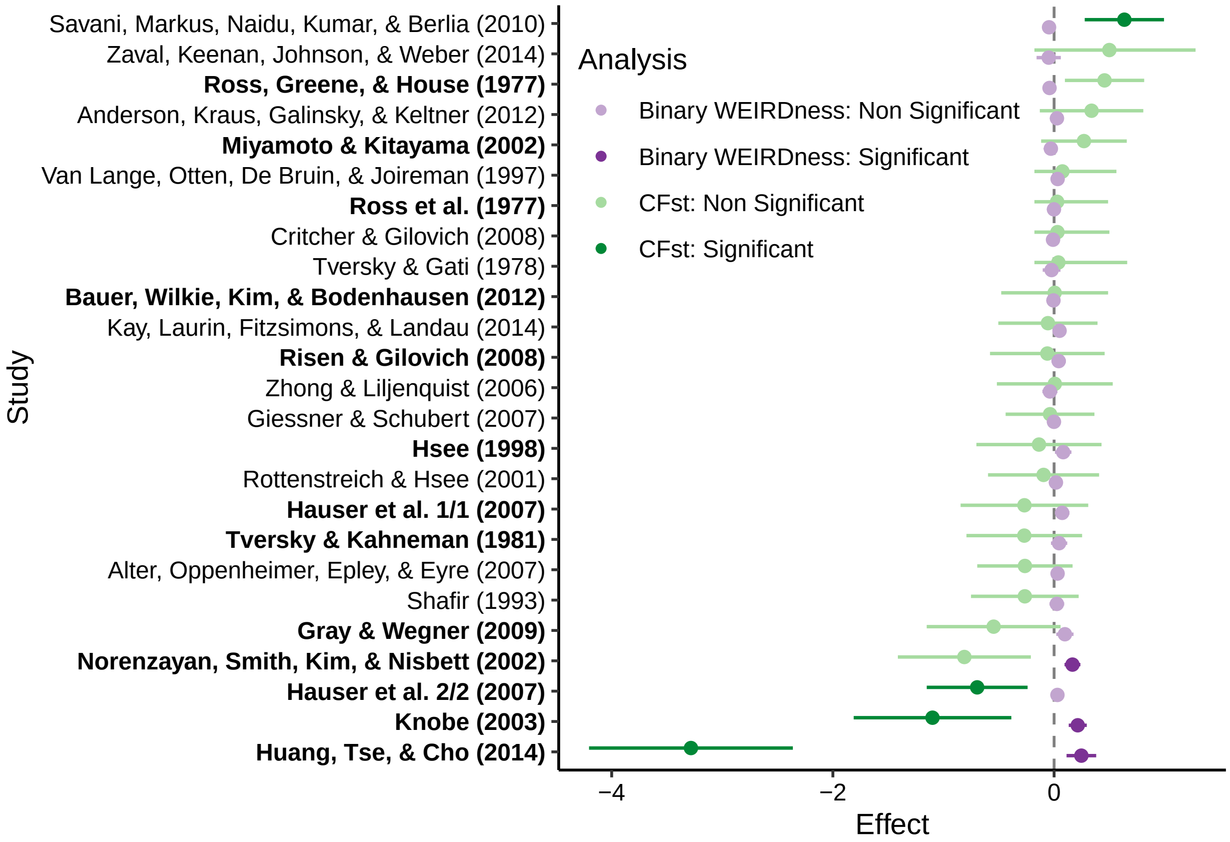

Supplementary Figure 5: Summary results for Analysis A. Forest plot showing the moderating role of CF_{ST} (green) and the ML2-WEIRDness variable (purple) on effect sizes. This shows that CF_{ST} has a significant moderator role in four studies (dark green) and that ML2-WEIRDness had a significant moderator role in three studies (dark purple). The significance threshold for the migration status analysis uses the alpha = 0.004 (Slate 1) and alpha = 0.003 (Slate 2) as calculated in the original ML2 analysis using Bonferroni correction for multiple comparisons. Studies in bold replicated in the original ML2 study. The error bars are the 2.5% and 97.5% confidence intervals.

Supplementary Table 9. **Analysis A**: Replication of the original analysis conducted by Klein et al. (2018). *p* values are adjusted using Bonferroni correction. The values in brackets represent the 2.5% and 97.5% confidence intervals.

Study	*N*	Effect	*p* value	Adjusted *p* value
Huang, Tse, & Cho (2014)	6381	0.25 [0.11, 0.38]	0.00031	0.0078
Kay, Laurin, Fitzsimons, & Landau (2014)	5806	0.049 [-0.0056, 0.1]	0.079	1
Alter, Oppenheimer, Epley, & Eyre (2007)	6749	0.032 [-0.021, 0.084]	0.24	1
Rottenstreich & Hsee (2001)	6578	0.017 [-0.046, 0.079]	0.6	1
Bauer, Wilkie, Kim, & Bodenhausen (2012)	5977	-0.0058 [-0.065, 0.053]	0.85	1
Miyamoto & Kitayama (2002)	6487	-0.029 [-0.076, 0.018]	0.23	1
Critcher & Gilovich (2008)	6219	-0.0097 [-0.068, 0.049]	0.75	1
Van Lange, Otten, De Bruin, & Joireman (1997)	5669	0.032 [-0.026, 0.09]	0.28	1
Hauser et al. 1/1 (2007)	6228	0.073 [0.0078, 0.14]	0.028	0.71
Anderson, Kraus, Galinsky, & Keltner (2012)	6277	0.026 [-0.03, 0.081]	0.36	1
Ross, Greene, & House (1977)	6528	-0.041 [-0.085, 0.0028]	0.067	1
Ross et al. (1977)	7727	-0.0013 [-0.062, 0.06]	0.97	1
Giessner & Schubert (2007)	7844	-0.0012 [-0.055, 0.053]	0.96	1
Tversky & Kahneman (1981)	7183	0.045 [-0.028, 0.12]	0.23	1
Hauser et al. 2/2 (2007)	7907	0.03 [-0.032, 0.093]	0.34	1
Risen & Gilovich (2008)	7954	0.042 [-0.023, 0.11]	0.2	1
Savani, Markus, Naidu, Kumar, & Berlia (2010)	5928	-0.046 [-0.092, 0.00094]	0.055	1
Norenzayan, Smith, Kim, & Nisbett (2002)	7317	0.17 [0.094, 0.24]	5.7e-06	0.00014
Hsee (1998)	7604	0.082 [0.0077, 0.16]	0.031	0.76
Gray & Wegner (2009)	7986	0.098 [0.02, 0.18]	0.014	0.35
Zhong & Liljenquist (2006)	6826	-0.038 [-0.11, 0.03]	0.28	1
Shafir (1993)	6504	0.025 [-0.041, 0.091]	0.46	1
Zaval, Keenan, Johnson, & Weber (2014)	3994	-0.049 [-0.16, 0.061]	0.38	1
Knobe (2003)	7936	0.21 [0.13, 0.3]	2.7e-07	6.8e-06
Tversky & Gati (1978)	3256	-0.023 [-0.1, 0.058]	0.58	1

Supplementary Table 10. **Analysis A**: Replication of the original analysis replacing the dichotomous WEIRDness score with a continuous proxy for a WEIRD scale—cultural distance from the United States (Muthukrishna et al., 2020), filtering countries missing cultural distance. *p* values are adjusted using Bonferroni correction. The values in brackets represent the 2.5% and 97.5% confidence intervals.

Study	*N*	Effect	*p* value	Adjusted *p* value
Huang, Tse, & Cho (2014)	6381	-3.3 [-4.2, -2.4]	2.9e-12	7.2e-11
Kay, Laurin, Fitzsimons, & Landau (2014)	5806	-0.056 [-0.5, 0.39]	0.81	1
Alter, Oppenheimer, Epley, & Eyre (2007)	6749	-0.26 [-0.69, 0.17]	0.23	1
Rottenstreich & Hsee (2001)	6578	-0.095 [-0.6, 0.41]	0.71	1
Bauer, Wilkie, Kim, & Bodenhausen (2012)	5977	0.0051 [-0.48, 0.49]	0.98	1
Miyamoto & Kitayama (2002)	6487	0.27 [-0.12, 0.66]	0.17	1
Critcher & Gilovich (2008)	6219	0.03 [-0.44, 0.5]	0.9	1
Van Lange, Otten, De Bruin, & Joireman (1997)	5669	0.075 [-0.41, 0.56]	0.76	1
Hauser et al. 1/1 (2007)	6228	-0.27 [-0.85, 0.31]	0.36	1
Anderson, Kraus, Galinsky, & Keltner (2012)	6277	0.34 [-0.13, 0.81]	0.16	1
Ross, Greene, & House (1977)	6528	0.46 [0.098, 0.81]	0.013	0.32
Ross et al. (1977)	7727	0.028 [-0.43, 0.49]	0.91	1
Giessner & Schubert (2007)	7844	-0.037 [-0.44, 0.36]	0.86	1
Tversky & Kahneman (1981)	7183	-0.27 [-0.79, 0.25]	0.31	1
Hauser et al. 2/2 (2007)	7907	-0.7 [-1.2, -0.24]	0.0028	0.07
Risen & Gilovich (2008)	7954	-0.061 [-0.58, 0.46]	0.82	1
Savani, Markus, Naidu, Kumar, & Berlia (2010)	5928	0.64 [0.28, 0.99]	0.00051	0.013
Norenzayan, Smith, Kim, & Nisbett (2002)	7317	-0.81 [-1.4, -0.21]	0.0082	0.2
Hsee (1998)	7604	-0.14 [-0.7, 0.43]	0.64	1
Gray & Wegner (2009)	7986	-0.55 [-1.2, 0.059]	0.077	1
Zhong & Liljenquist (2006)	6826	0.0065 [-0.52, 0.53]	0.98	1
Shafir (1993)	6504	-0.26 [-0.75, 0.22]	0.29	1
Zaval, Keenan, Johnson, & Weber (2014)	3994	0.5 [-0.28, 1.3]	0.21	1
Knobe (2003)	7936	-1.1 [-1.8, -0.39]	0.0025	0.062
Tversky & Gati (1978)	3256	0.038 [-0.58, 0.66]	0.9	1

Supplementary Table 11. **Analysis A**: Replication of the original analysis replacing the dichotomous WEIRDness score with a continuous proxy for a WEIRD scale—cultural distance from the United States (Muthukrishna et al., 2020), *imputing the* CF_{ST} *for countries missing cultural distance, using an average of their nearest neighbors*. *p* values are adjusted using Bonferroni correction. The values in brackets represent the 2.5% and 97.5% confidence intervals.

Study	*N*	Effect	*p* value	Adjusted *p* value
Huang, Tse, & Cho (2014)	6381	-3 [-3.9, -2.1]	1.3e-10	3.2e-09
Kay, Laurin, Fitzsimons, & Landau (2014)	5806	-0.072 [-0.5, 0.36]	0.74	1
Alter, Oppenheimer, Epley, & Eyre (2007)	6749	-0.21 [-0.62, 0.21]	0.32	1
Rottenstreich & Hsee (2001)	6578	-0.073 [-0.55, 0.41]	0.77	1
Bauer, Wilkie, Kim, & Bodenhausen (2012)	5977	-0.079 [-0.55, 0.39]	0.74	1
Miyamoto & Kitayama (2002)	6487	0.28 [-0.089, 0.65]	0.14	1
Critcher & Gilovich (2008)	6219	-0.027 [-0.49, 0.43]	0.91	1
Van Lange, Otten, De Bruin, & Joireman (1997)	5669	0.09 [-0.37, 0.55]	0.7	1
Hauser et al. 1/1 (2007)	6228	-0.26 [-0.79, 0.28]	0.35	1
Anderson, Kraus, Galinsky, & Keltner (2012)	6277	0.23 [-0.22, 0.68]	0.32	1
Ross, Greene, & House (1977)	6528	0.51 [0.17, 0.85]	0.003	0.075
Ross et al. (1977)	7727	-0.14 [-0.59, 0.31]	0.55	1
Giessner & Schubert (2007)	7844	-0.016 [-0.4, 0.37]	0.93	1
Tversky & Kahneman (1981)	7183	-0.38 [-0.85, 0.085]	0.11	1
Hauser et al. 2/2 (2007)	7907	-0.55 [-0.97, -0.13]	0.01	0.26
Risen & Gilovich (2008)	7954	-0.057 [-0.54, 0.42]	0.82	1
Savani, Markus, Naidu, Kumar, & Berlia (2010)	5928	0.47 [0.12, 0.81]	0.0083	0.21
Norenzayan, Smith, Kim, & Nisbett (2002)	7317	-0.64 [-1.2, -0.07]	0.028	0.69
Hsee (1998)	7604	-0.21 [-0.76, 0.34]	0.45	1
Gray & Wegner (2009)	7986	-0.56 [-1.1, 0.013]	0.056	1
Zhong & Liljenquist (2006)	6826	0.11 [-0.37, 0.58]	0.66	1
Shafir (1993)	6504	-0.37 [-0.82, 0.074]	0.1	1
Zaval, Keenan, Johnson, & Weber (2014)	3994	0.32 [-0.39, 1]	0.38	1
Knobe (2003)	7936	-1.1 [-1.8, -0.44]	0.0011	0.027
Tversky & Gati (1978)	3256	0.062 [-0.51, 0.63]	0.83	1

2.3.2 Analysis B Results

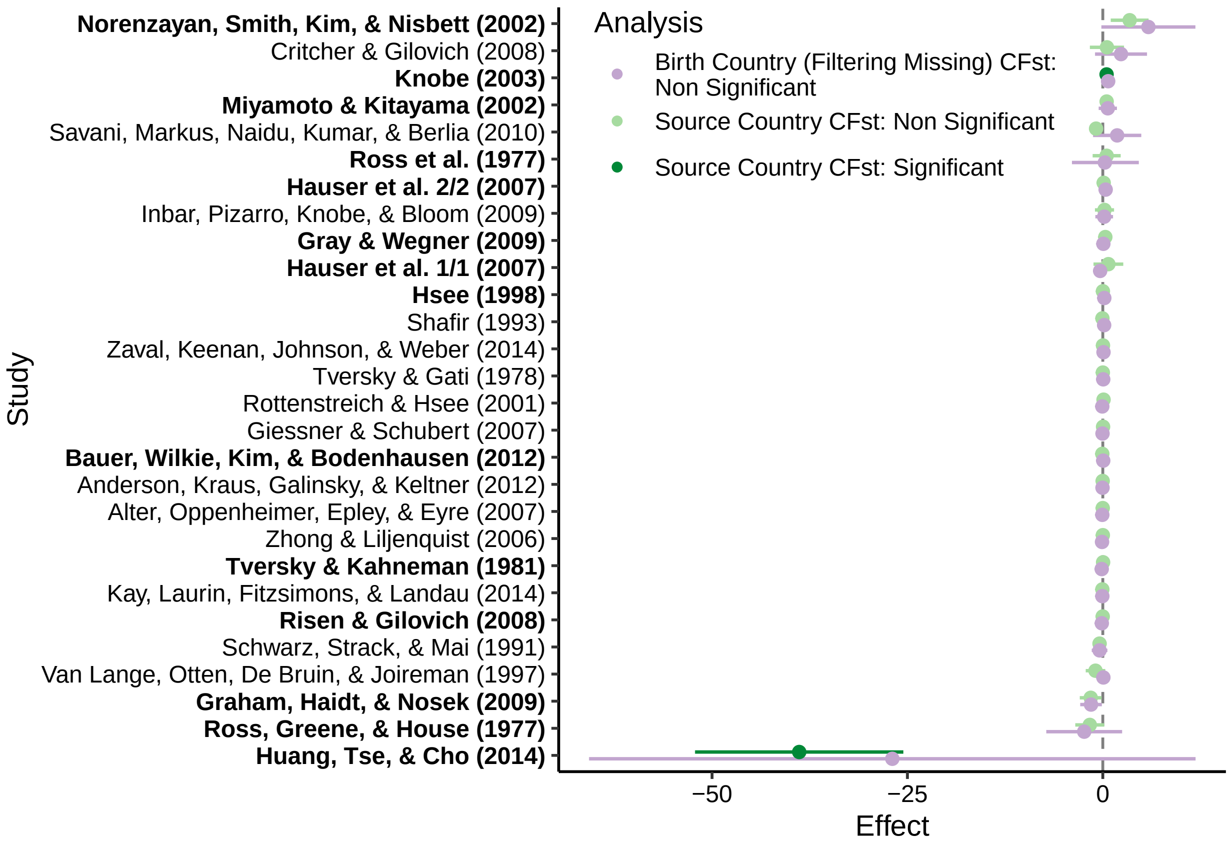

Supplementary Figure 6: Summary results for Analysis B1 (source country CF_{ST}) and B2 (birth country CF_{ST}). Forest plot showing the moderating role of CF_{ST} source country (green) and CF_{ST} birth country (purple) on effect sizes based on the estimates from the multilevel model. CF_{ST} identified by source countries is significant for two studies (indicated in dark green) and there were no significant results for CF_{ST} identified by the birth country of participants.The significance threshold for the migration status analysis uses the alpha = 0.004 (Slate 1) and alpha = 0.003 (Slate 2) as calculated in the original ML2 analysis using Bonferroni correction for multiple comparisons. Studies in bold replicated in the original ML2 study. The error bars are the 2.5% and 97.5% confidence intervals.

Supplementary Table 12. **Analysis B1**: Mixed effect model, where participants are clustered by source country, filtering countries missing cultural distance, and using an inclusion criteria of a *minimum of 100 participants per country*. *p* values are adjusted using Bonferroni correction. The values in brackets represent the 2.5% and 97.5% confidence intervals.

Study	*N*	Effect	*p* value	Adjusted *p* value
Huang, Tse, & Cho (2014)	5920	-40 [-52, -27]	3.4e-08	9.6e-07
Kay, Laurin, Fitzsimons, & Landau (2014)	6014	-0.057 [-0.19, 0.076]	0.4	1
Alter, Oppenheimer, Epley, & Eyre (2007)	6696	-0.013 [-0.063, 0.037]	0.6	1
Graham, Haidt, & Nosek (2009)	6782	-1.5 [-3, -0.017]	0.048	1
Rottenstreich & Hsee (2001)	6739	0.076 [-0.05, 0.2]	0.23	1
Bauer, Wilkie, Kim, & Bodenhausen (2012)	6175	-0.063 [-0.16, 0.034]	0.2	1
Miyamoto & Kitayama (2002)	6720	0.56 [-0.0083, 1.1]	0.053	1
Inbar, Pizarro, Knobe, & Bloom (2009)	6730	0.22 [-1.1, 1.5]	0.74	1
Critcher & Gilovich (2008)	6054	0.68 [-1.5, 2.8]	0.53	1
Van Lange, Otten, De Bruin, & Joireman (1997)	5507	-0.93 [-2.3, 0.43]	0.18	1
Hauser et al. 1/1 (2007)	6724	0.79 [-1.2, 2.8]	0.44	1
Anderson, Kraus, Galinsky, & Keltner (2012)	6757	-0.016 [-0.055, 0.024]	0.43	1
Ross, Greene, & House (1977)	6348	-1.8 [-3.6, 0.12]	0.066	1
Ross et al. (1977)	6454	0.27 [-1.8, 2.3]	0.79	1
Giessner & Schubert (2007)	6902	0.049 [-0.059, 0.16]	0.37	1
Tversky & Kahneman (1981)	6531	0.021 [-0.14, 0.19]	0.8	1
Hauser et al. 2/2 (2007)	7223	0.21 [0.072, 0.36]	0.0041	0.11
Risen & Gilovich (2008)	7197	0.054 [-0.11, 0.22]	0.51	1
Savani, Markus, Naidu, Kumar, & Berlia (2010)	7299	-0.26 [-0.52, 0.0052]	0.054	1
Norenzayan, Smith, Kim, & Nisbett (2002)	7308	1.4 [-1, 3.8]	0.26	1
Hsee (1998)	6993	-0.041 [-0.15, 0.063]	0.43	1
Gray & Wegner (2009)	7294	0.22 [0.018, 0.42]	0.034	0.94
Zhong & Liljenquist (2006)	6395	-0.022 [-0.13, 0.092]	0.7	1
Schwarz, Strack, & Mai (1991)	6438	-0.46 [-1.6, 0.72]	0.44	1
Shafir (1993)	7277	0.0074 [-0.18, 0.19]	0.94	1
Zaval, Keenan, Johnson, & Weber (2014)	3222	-0.019 [-0.12, 0.077]	0.69	1
Knobe (2003)	7273	0.21 [-0.092, 0.52]	0.17	1
Tversky & Gati (1978)	2600	0.0086 [-0.081, 0.098]	0.85	1

Supplementary Table 13. **Analysis B1**: Mixed effect model, where participants are clustered by source country, filtering countries missing cultural distance, and using an inclusion criteria of a *minimum of 50 participants per country*. *p* values are adjusted using Bonferroni correction. The values in brackets represent the 2.5% and 97.5% confidence intervals.

Study	*N*	Effect	*p* value	Adjusted *p* value
Huang, Tse, & Cho (2014)	6488	-39 [-52, -26]	1.8e-07	5e-06
Kay, Laurin, Fitzsimons, & Landau (2014)	6411	-0.063 [-0.2, 0.075]	0.37	1
Alter, Oppenheimer, Epley, & Eyre (2007)	7091	-0.015 [-0.066, 0.036]	0.56	1
Graham, Haidt, & Nosek (2009)	7181	-1.5 [-2.9, -0.13]	0.033	0.93
Rottenstreich & Hsee (2001)	7136	0.091 [-0.037, 0.22]	0.16	1
Bauer, Wilkie, Kim, & Bodenhausen (2012)	6574	-0.061 [-0.16, 0.035]	0.21	1
Miyamoto & Kitayama (2002)	7116	0.48 [-0.22, 1.2]	0.18	1
Inbar, Pizarro, Knobe, & Bloom (2009)	7036	0.2 [-1, 1.4]	0.74	1
Critcher & Gilovich (2008)	6708	0.56 [-1.6, 2.7]	0.61	1
Van Lange, Otten, De Bruin, & Joireman (1997)	6115	-0.91 [-2.2, 0.35]	0.15	1
Hauser et al. 1/1 (2007)	7121	0.72 [-1.2, 2.6]	0.46	1
Anderson, Kraus, Galinsky, & Keltner (2012)	7156	-0.017 [-0.06, 0.026]	0.44	1
Ross, Greene, & House (1977)	6925	-1.6 [-3.4, 0.22]	0.083	1
Ross et al. (1977)	7355	0.51 [-1.4, 2.4]	0.59	1
Giessner & Schubert (2007)	7842	0.033 [-0.059, 0.13]	0.47	1
Tversky & Kahneman (1981)	7179	0.055 [-0.1, 0.21]	0.48	1
Hauser et al. 2/2 (2007)	7869	0.11 [-0.027, 0.25]	0.11	1
Risen & Gilovich (2008)	7952	0.011 [-0.14, 0.17]	0.89	1
Savani, Markus, Naidu, Kumar, & Berlia (2010)	7953	-0.96 [-1.7, -0.18]	0.017	0.46
Norenzayan, Smith, Kim, & Nisbett (2002)	7968	3.1 [0.6, 5.5]	0.016	0.45
Hsee (1998)	7642	-0.038 [-0.13, 0.056]	0.42	1
Gray & Wegner (2009)	7953	0.35 [0.11, 0.59]	0.0045	0.13
Zhong & Liljenquist (2006)	6938	-0.01 [-0.11, 0.091]	0.84	1
Schwarz, Strack, & Mai (1991)	7413	-0.48 [-1.4, 0.46]	0.31	1
Shafir (1993)	7852	-0.072 [-0.24, 0.094]	0.39	1
Zaval, Keenan, Johnson, & Weber (2014)	4041	0.017 [-0.059, 0.094]	0.65	1
Knobe (2003)	7933	0.46 [0.14, 0.77]	0.0055	0.15
Tversky & Gati (1978)	3003	-0.0038 [-0.076, 0.068]	0.92	1

Supplementary Table 14. **Analysis B1**: Mixed effect model, where participants are clustered by source country, filtering countries missing cultural distance, and using an inclusion criteria of a *minimum of 36 participants per country*. *p* values are adjusted using Bonferroni correction. The values in brackets represent the 2.5% and 97.5% confidence intervals.

Study	*N*	Effect	*p* value	Adjusted *p* value
Huang, Tse, & Cho (2014)	6537	-39 [-52, -26]	1.9e-07	5.3e-06
Kay, Laurin, Fitzsimons, & Landau (2014)	6455	-0.061 [-0.2, 0.078]	0.39	1
Alter, Oppenheimer, Epley, & Eyre (2007)	7135	-0.012 [-0.067, 0.043]	0.66	1
Graham, Haidt, & Nosek (2009)	7261	-1.5 [-2.9, -0.15]	0.031	0.87
Rottenstreich & Hsee (2001)	7216	0.095 [-0.035, 0.22]	0.15	1
Bauer, Wilkie, Kim, & Bodenhausen (2012)	6654	-0.066 [-0.17, 0.035]	0.2	1
Miyamoto & Kitayama (2002)	7160	0.49 [-0.21, 1.2]	0.17	1
Inbar, Pizarro, Knobe, & Bloom (2009)	7080	0.21 [-1, 1.4]	0.73	1
Critcher & Gilovich (2008)	6752	0.53 [-1.7, 2.7]	0.63	1
Van Lange, Otten, De Bruin, & Joireman (1997)	6206	-0.94 [-2.2, 0.32]	0.14	1
Hauser et al. 1/1 (2007)	7201	0.71 [-1.2, 2.6]	0.46	1
Anderson, Kraus, Galinsky, & Keltner (2012)	7236	-0.017 [-0.06, 0.026]	0.43	1
Ross, Greene, & House (1977)	6968	-1.7 [-3.5, 0.19]	0.078	1
Ross et al. (1977)	7440	0.49 [-1.3, 2.3]	0.59	1
Giessner & Schubert (2007)	7889	0.032 [-0.058, 0.12]	0.48	1
Tversky & Kahneman (1981)	7227	0.035 [-0.11, 0.19]	0.64	1
Hauser et al. 2/2 (2007)	7917	0.11 [-0.026, 0.25]	0.11	1
Risen & Gilovich (2008)	7999	-0.015 [-0.17, 0.14]	0.84	1
Savani, Markus, Naidu, Kumar, & Berlia (2010)	8001	-0.86 [-1.6, -0.1]	0.026	0.74
Norenzayan, Smith, Kim, & Nisbett (2002)	8016	3.4 [1, 5.9]	0.0063	0.18
Hsee (1998)	7690	0.01 [-0.095, 0.12]	0.84	1
Gray & Wegner (2009)	8001	0.32 [0.084, 0.55]	0.0088	0.25
Zhong & Liljenquist (2006)	7022	0.0011 [-0.095, 0.097]	0.98	1
Schwarz, Strack, & Mai (1991)	7459	-0.42 [-1.3, 0.48]	0.36	1
Shafir (1993)	7900	-0.052 [-0.21, 0.11]	0.52	1
Zaval, Keenan, Johnson, & Weber (2014)	4133	0.02 [-0.055, 0.095]	0.59	1
Knobe (2003)	7981	0.48 [0.17, 0.79]	0.0027	0.075
Tversky & Gati (1978)	3381	0.0029 [-0.076, 0.082]	0.94	1

Supplementary Table 15. **Analysis B1**: Mixed effect model, where participants are clustered by source country, filtering countries missing cultural distance, and using an inclusion criteria of a *minimum of 10 participants per country*. *p* values are adjusted using Bonferroni correction. The values in brackets represent the 2.5% and 97.5% confidence intervals.

Study	*N*	Effect	*p* value	Adjusted *p* value
Huang, Tse, & Cho (2014)	6587	-39 [-53, -26]	1.7e-07	4.9e-06
Kay, Laurin, Fitzsimons, & Landau (2014)	6490	-0.061 [-0.2, 0.078]	0.39	1
Alter, Oppenheimer, Epley, & Eyre (2007)	7170	-0.012 [-0.067, 0.043]	0.66	1
Graham, Haidt, & Nosek (2009)	7261	-1.6 [-3, -0.15]	0.031	0.87
Rottenstreich & Hsee (2001)	7216	0.095 [-0.035, 0.23]	0.15	1
Bauer, Wilkie, Kim, & Bodenhausen (2012)	6654	-0.067 [-0.17, 0.035]	0.2	1
Miyamoto & Kitayama (2002)	7195	0.49 [-0.21, 1.2]	0.17	1
Inbar, Pizarro, Knobe, & Bloom (2009)	7115	0.21 [-1, 1.4]	0.73	1
Critcher & Gilovich (2008)	6785	0.53 [-1.7, 2.7]	0.63	1
Van Lange, Otten, De Bruin, & Joireman (1997)	6239	-0.94 [-2.2, 0.32]	0.14	1
Hauser et al. 1/1 (2007)	7201	0.71 [-1.2, 2.6]	0.46	1
Anderson, Kraus, Galinsky, & Keltner (2012)	7236	-0.017 [-0.06, 0.026]	0.43	1
Ross, Greene, & House (1977)	7002	-1.7 [-3.5, 0.19]	0.078	1
Ross et al. (1977)	7440	0.49 [-1.3, 2.3]	0.59	1
Giessner & Schubert (2007)	7889	0.032 [-0.058, 0.12]	0.48	1
Tversky & Kahneman (1981)	7227	0.035 [-0.11, 0.19]	0.64	1
Hauser et al. 2/2 (2007)	7917	0.11 [-0.026, 0.25]	0.11	1
Risen & Gilovich (2008)	7999	-0.015 [-0.17, 0.14]	0.84	1
Savani, Markus, Naidu, Kumar, & Berlia (2010)	8001	-0.87 [-1.6, -0.11]	0.026	0.74
Norenzayan, Smith, Kim, & Nisbett (2002)	8016	3.4 [1, 5.9]	0.0063	0.18
Hsee (1998)	7690	0.01 [-0.095, 0.12]	0.84	1
Gray & Wegner (2009)	8001	0.32 [0.084, 0.55]	0.0088	0.25
Zhong & Liljenquist (2006)	7056	-0.0012 [-0.098, 0.096]	0.98	1
Schwarz, Strack, & Mai (1991)	7459	-0.42 [-1.3, 0.49]	0.36	1
Shafir (1993)	7900	-0.052 [-0.22, 0.11]	0.52	1
Zaval, Keenan, Johnson, & Weber (2014)	4202	0.028 [-0.046, 0.1]	0.45	1
Knobe (2003)	7981	0.48 [0.18, 0.79]	0.0027	0.075
Tversky & Gati (1978)	3549	0.011 [-0.064, 0.086]	0.77	1

Supplementary Table 16. **Analysis B1**: Mixed effect model, where participants are clustered by source country, imputing the CF_{ST} for countries missing cultural distance, using an inclusion criteria of a *minimum of 100 participants per country*. *p* values are adjusted using Bonferroni correction. The values in brackets represent the 2.5% and 97.5% confidence intervals.

Study	*N*	Effect	*p* value	Adjusted *p* value
Huang, Tse, & Cho (2014)	5920	-40 [-52, -27]	1.8e-08	5e-07
Kay, Laurin, Fitzsimons, & Landau (2014)	6014	-0.053 [-0.18, 0.072]	0.4	1
Alter, Oppenheimer, Epley, & Eyre (2007)	6696	-0.0041 [-0.052, 0.044]	0.87	1
Graham, Haidt, & Nosek (2009)	6782	-1.4 [-2.8, -0.014]	0.048	1
Rottenstreich & Hsee (2001)	6739	0.055 [-0.067, 0.18]	0.37	1
Bauer, Wilkie, Kim, & Bodenhausen (2012)	6175	-0.08 [-0.17, 0.013]	0.091	1
Miyamoto & Kitayama (2002)	6720	0.57 [0.027, 1.1]	0.04	1
Inbar, Pizarro, Knobe, & Bloom (2009)	6730	0.18 [-1, 1.4]	0.77	1
Critcher & Gilovich (2008)	6054	0.5 [-1.7, 2.7]	0.65	1
Van Lange, Otten, De Bruin, & Joireman (1997)	5507	-0.97 [-2.3, 0.38]	0.15	1
Hauser et al. 1/1 (2007)	6724	0.65 [-1.2, 2.6]	0.5	1
Anderson, Kraus, Galinsky, & Keltner (2012)	6757	-0.012 [-0.05, 0.026]	0.54	1
Ross, Greene, & House (1977)	6348	-1.8 [-3.7, 0.049]	0.056	1
Ross et al. (1977)	6454	1 [-0.96, 3]	0.3	1
Giessner & Schubert (2007)	6902	0.044 [-0.061, 0.15]	0.4	1
Tversky & Kahneman (1981)	6531	0.068 [-0.079, 0.21]	0.36	1
Hauser et al. 2/2 (2007)	7223	0.17 [0.043, 0.3]	0.01	0.28
Risen & Gilovich (2008)	7197	0.046 [-0.11, 0.2]	0.55	1
Savani, Markus, Naidu, Kumar, & Berlia (2010)	7299	-0.21 [-0.45, 0.026]	0.079	1
Norenzayan, Smith, Kim, & Nisbett (2002)	7308	1 [-1.2, 3.2]	0.37	1
Hsee (1998)	6993	-0.045 [-0.15, 0.056]	0.37	1
Gray & Wegner (2009)	7294	0.21 [0.025, 0.39]	0.027	0.75
Zhong & Liljenquist (2006)	6395	-0.027 [-0.13, 0.075]	0.6	1
Schwarz, Strack, & Mai (1991)	6438	-0.41 [-1.5, 0.66]	0.44	1
Shafir (1993)	7277	-0.025 [-0.19, 0.14]	0.76	1
Zaval, Keenan, Johnson, & Weber (2014)	3222	-0.019 [-0.12, 0.077]	0.69	1
Knobe (2003)	7273	0.21 [-0.064, 0.48]	0.13	1
Tversky & Gati (1978)	2600	0.0086 [-0.081, 0.098]	0.85	1

Supplementary Table 17. **Analysis B1**: Mixed effect model, where participants are clustered by source country, imputing the CF_{ST} for countries missing cultural distance, using an inclusion criteria of a *minimum of 50 participants per country*. *p* values are adjusted using Bonferroni correction. The values in brackets represent the 2.5% and 97.5% confidence intervals.

Study	*N*	Effect	*p* value	Adjusted *p* value
Huang, Tse, & Cho (2014)	6488	-36 [-48, -23]	4.1e-07	1.2e-05
Kay, Laurin, Fitzsimons, & Landau (2014)	6411	-0.053 [-0.18, 0.076]	0.41	1
Alter, Oppenheimer, Epley, & Eyre (2007)	7091	-0.0085 [-0.058, 0.041]	0.73	1
Graham, Haidt, & Nosek (2009)	7181	-1.5 [-2.8, -0.18]	0.027	0.75
Rottenstreich & Hsee (2001)	7136	0.076 [-0.047, 0.2]	0.22	1
Bauer, Wilkie, Kim, & Bodenhausen (2012)	6574	-0.073 [-0.16, 0.018]	0.11	1
Miyamoto & Kitayama (2002)	7116	0.5 [-0.16, 1.2]	0.14	1
Inbar, Pizarro, Knobe, & Bloom (2009)	7036	0.17 [-0.99, 1.3]	0.77	1
Critcher & Gilovich (2008)	6708	0.18 [-1.9, 2.3]	0.86	1
Van Lange, Otten, De Bruin, & Joireman (1997)	6115	-0.95 [-2.1, 0.23]	0.11	1
Hauser et al. 1/1 (2007)	7121	0.56 [-1.2, 2.3]	0.53	1
Anderson, Kraus, Galinsky, & Keltner (2012)	7156	-0.015 [-0.055, 0.026]	0.48	1
Ross, Greene, & House (1977)	6925	-1.8 [-3.5, -0.078]	0.041	1
Ross et al. (1977)	7355	1.2 [-0.64, 3.1]	0.19	1
Giessner & Schubert (2007)	7842	0.034 [-0.058, 0.13]	0.46	1
Tversky & Kahneman (1981)	7179	0.077 [-0.066, 0.22]	0.28	1
Hauser et al. 2/2 (2007)	7869	0.099 [-0.031, 0.23]	0.13	1
Risen & Gilovich (2008)	7952	0.0083 [-0.14, 0.15]	0.91	1
Savani, Markus, Naidu, Kumar, & Berlia (2010)	7953	-0.83 [-1.6, -0.11]	0.024	0.67
Norenzayan, Smith, Kim, & Nisbett (2002)	7968	2.6 [0.21, 4.9]	0.033	0.93
Hsee (1998)	7642	-0.042 [-0.13, 0.051]	0.37	1
Gray & Wegner (2009)	7953	0.33 [0.1, 0.55]	0.0049	0.14
Zhong & Liljenquist (2006)	6938	-0.018 [-0.11, 0.074]	0.69	1
Schwarz, Strack, & Mai (1991)	7413	-0.43 [-1.3, 0.43]	0.32	1
Shafir (1993)	7852	-0.085 [-0.24, 0.069]	0.27	1
Zaval, Keenan, Johnson, & Weber (2014)	4041	0.012 [-0.058, 0.083]	0.72	1
Knobe (2003)	7933	0.43 [0.13, 0.72]	0.0055	0.15
Tversky & Gati (1978)	3003	0.0078 [-0.058, 0.073]	0.81	1

Supplementary Table 18. **Analysis B1**: Mixed effect model, where participants are clustered by source country, imputing the CF_{ST} for countries missing cultural distance, using an inclusion criteria of a *minimum of 36 participants per country*. *p* values are adjusted using Bonferroni correction. The values in brackets represent the 2.5% and 97.5% confidence intervals.

Study	*N*	Effect	*p* value	Adjusted *p* value
Huang, Tse, & Cho (2014)	6537	-36 [-48, -23]	4.2e-07	1.2e-05
Kay, Laurin, Fitzsimons, & Landau (2014)	6455	-0.051 [-0.18, 0.078]	0.43	1
Alter, Oppenheimer, Epley, & Eyre (2007)	7135	-0.0065 [-0.059, 0.046]	0.8	1
Graham, Haidt, & Nosek (2009)	7261	-1.5 [-2.8, -0.21]	0.024	0.66
Rottenstreich & Hsee (2001)	7216	0.079 [-0.044, 0.2]	0.2	1
Bauer, Wilkie, Kim, & Bodenhausen (2012)	6654	-0.076 [-0.17, 0.02]	0.12	1
Miyamoto & Kitayama (2002)	7160	0.5 [-0.15, 1.2]	0.13	1
Inbar, Pizarro, Knobe, & Bloom (2009)	7080	0.18 [-0.98, 1.3]	0.76	1
Critcher & Gilovich (2008)	6752	0.16 [-1.9, 2.2]	0.88	1
Van Lange, Otten, De Bruin, & Joireman (1997)	6206	-0.97 [-2.1, 0.2]	0.1	1
Hauser et al. 1/1 (2007)	7201	0.55 [-1.2, 2.3]	0.53	1
Anderson, Kraus, Galinsky, & Keltner (2012)	7236	-0.015 [-0.056, 0.025]	0.45	1
Ross, Greene, & House (1977)	6968	-1.8 [-3.6, -0.094]	0.039	1
Ross et al. (1977)	7440	1.1 [-0.72, 2.8]	0.24	1
Giessner & Schubert (2007)	7889	0.033 [-0.057, 0.12]	0.46	1
Tversky & Kahneman (1981)	7227	0.058 [-0.081, 0.2]	0.41	1
Hauser et al. 2/2 (2007)	7917	0.096 [-0.03, 0.22]	0.13	1
Risen & Gilovich (2008)	7999	-0.015 [-0.16, 0.13]	0.84	1
Savani, Markus, Naidu, Kumar, & Berlia (2010)	8001	-0.76 [-1.5, -0.054]	0.035	0.99
Norenzayan, Smith, Kim, & Nisbett (2002)	8016	2.9 [0.6, 5.3]	0.014	0.41
Hsee (1998)	7690	0.00047 [-0.1, 0.1]	0.99	1
Gray & Wegner (2009)	8001	0.3 [0.078, 0.52]	0.0088	0.25
Zhong & Liljenquist (2006)	7022	-0.0088 [-0.098, 0.08]	0.84	1
Schwarz, Strack, & Mai (1991)	7459	-0.38 [-1.2, 0.45]	0.36	1
Shafir (1993)	7900	-0.067 [-0.22, 0.084]	0.38	1
Zaval, Keenan, Johnson, & Weber (2014)	4133	0.015 [-0.055, 0.085]	0.68	1
Knobe (2003)	7981	0.45 [0.16, 0.74]	0.0028	0.078
Tversky & Gati (1978)	3381	0.0076 [-0.065, 0.08]	0.83	1

Supplementary Table 19. **Analysis B1**: Mixed effect model, where participants are clustered by source country, imputing the CF_{ST} for countries missing cultural distance, using an inclusion criteria of a *minimum of 10 participants per country*. *p* values are adjusted using Bonferroni correction. The values in brackets represent the 2.5% and 97.5% confidence intervals.

Study	*N*	Effect	*p* value	Adjusted *p* value
Huang, Tse, & Cho (2014)	6587	-36 [-49, -23]	3.3e-07	9.3e-06
Kay, Laurin, Fitzsimons, & Landau (2014)	6490	-0.053 [-0.18, 0.077]	0.42	1
Alter, Oppenheimer, Epley, & Eyre (2007)	7170	-0.0086 [-0.064, 0.047]	0.76	1
Graham, Haidt, & Nosek (2009)	7261	-1.5 [-2.8, -0.21]	0.024	0.66
Rottenstreich & Hsee (2001)	7216	0.08 [-0.044, 0.2]	0.2	1
Bauer, Wilkie, Kim, & Bodenhausen (2012)	6654	-0.076 [-0.17, 0.02]	0.12	1
Miyamoto & Kitayama (2002)	7195	0.5 [-0.15, 1.2]	0.13	1
Inbar, Pizarro, Knobe, & Bloom (2009)	7115	0.18 [-0.97, 1.3]	0.75	1
Critcher & Gilovich (2008)	6785	0.16 [-1.9, 2.2]	0.88	1
Van Lange, Otten, De Bruin, & Joireman (1997)	6239	-0.97 [-2.1, 0.19]	0.099	1
Hauser et al. 1/1 (2007)	7201	0.56 [-1.2, 2.3]	0.53	1
Anderson, Kraus, Galinsky, & Keltner (2012)	7236	-0.016 [-0.056, 0.025]	0.45	1
Ross, Greene, & House (1977)	7002	-1.8 [-3.6, -0.1]	0.038	1
Ross et al. (1977)	7440	1.1 [-0.73, 2.9]	0.24	1
Giessner & Schubert (2007)	7889	0.033 [-0.057, 0.12]	0.46	1
Tversky & Kahneman (1981)	7227	0.058 [-0.081, 0.2]	0.41	1
Hauser et al. 2/2 (2007)	7917	0.097 [-0.03, 0.22]	0.13	1
Risen & Gilovich (2008)	7999	-0.015 [-0.16, 0.13]	0.84	1
Savani, Markus, Naidu, Kumar, & Berlia (2010)	8001	-0.76 [-1.5, -0.054]	0.035	0.99
Norenzayan, Smith, Kim, & Nisbett (2002)	8016	2.9 [0.61, 5.3]	0.014	0.41
Hsee (1998)	7690	0.00047 [-0.1, 0.1]	0.99	1
Gray & Wegner (2009)	8001	0.3 [0.078, 0.52]	0.0088	0.25
Zhong & Liljenquist (2006)	7056	-0.01 [-0.1, 0.081]	0.83	1
Schwarz, Strack, & Mai (1991)	7459	-0.38 [-1.2, 0.45]	0.36	1
Shafir (1993)	7900	-0.068 [-0.22, 0.084]	0.38	1
Zaval, Keenan, Johnson, & Weber (2014)	4202	0.02 [-0.048, 0.089]	0.55	1
Knobe (2003)	7981	0.45 [0.16, 0.74]	0.0028	0.078
Tversky & Gati (1978)	3549	0.013 [-0.057, 0.083]	0.71	1

Supplementary Table 20. **Analysis B1**: Mixed effect model, where participants are clustered by source country and WEIRDness is operationalizated using the binary WEIRD score used by Klein et al. (2018). A *minimum of 100 participants per country* is used as an inclusion criteria. *p* values are adjusted using Bonferroni correction. The values in brackets represent the 2.5% and 97.5% confidence intervals.

Study	*N*	Effect	*p* value	Adjusted *p* value
Huang, Tse, & Cho (2014)	5920	3.3 [1.5, 5.2]	0.00061	0.017
Kay, Laurin, Fitzsimons, & Landau (2014)	6014	-0.00083 [-0.017, 0.015]	0.92	1
Alter, Oppenheimer, Epley, & Eyre (2007)	6696	0.0025 [-0.0035, 0.0086]	0.41	1
Graham, Haidt, & Nosek (2009)	6782	0.16 [-0.021, 0.33]	0.083	1
Rottenstreich & Hsee (2001)	6739	-0.0069 [-0.022, 0.0084]	0.37	1
Bauer, Wilkie, Kim, & Bodenhausen (2012)	6175	0.0092 [-0.0028, 0.021]	0.13	1
Miyamoto & Kitayama (2002)	6720	-0.046 [-0.12, 0.024]	0.19	1
Inbar, Pizarro, Knobe, & Bloom (2009)	6730	-0.033 [-0.19, 0.12]	0.66	1
Critcher & Gilovich (2008)	6054	-0.063 [-0.34, 0.21]	0.65	1
Van Lange, Otten, De Bruin, & Joireman (1997)	5507	0.11 [-0.065, 0.28]	0.21	1
Hauser et al. 1/1 (2007)	6724	-0.017 [-0.26, 0.22]	0.89	1
Anderson, Kraus, Galinsky, & Keltner (2012)	6757	-0.00099 [-0.0058, 0.0038]	0.68	1
Ross, Greene, & House (1977)	6348	0.11 [-0.12, 0.35]	0.35	1
Ross et al. (1977)	6454	0.056 [-0.27, 0.38]	0.73	1
Giessner & Schubert (2007)	6902	-0.01 [-0.027, 0.0073]	0.25	1
Tversky & Kahneman (1981)	6531	-0.024 [-0.049, 0.0013]	0.063	1
Hauser et al. 2/2 (2007)	7223	-0.013 [-0.034, 0.007]	0.19	1
Risen & Gilovich (2008)	7197	0.01 [-0.012, 0.033]	0.36	1
Savani, Markus, Naidu, Kumar, & Berlia (2010)	7299	0.019 [-0.017, 0.056]	0.29	1
Norenzayan, Smith, Kim, & Nisbett (2002)	7308	-0.52 [-0.82, -0.22]	0.0012	0.031
Hsee (1998)	6993	0.023 [0.0089, 0.036]	0.0018	0.049
Gray & Wegner (2009)	7294	-0.021 [-0.049, 0.0076]	0.15	1
Zhong & Liljenquist (2006)	6395	0.0088 [-0.0066, 0.024]	0.26	1
Schwarz, Strack, & Mai (1991)	6438	0.05 [-0.12, 0.22]	0.55	1
Shafir (1993)	7277	0.0028 [-0.022, 0.028]	0.82	1
Knobe (2003)	7273	-0.06 [-0.098, -0.022]	0.0028	0.076
Tversky & Gati (1978)	2600	0.0013 [-0.013, 0.015]	0.85	1

Supplementary Table 21. **Analysis B1**: Mixed effect model, where participants are clustered by source country and WEIRDness is operationalizated using the binary WEIRD score used by Klein et al. (2018). A *minimum of 50 participants per country* is used as an inclusion criteria. *p* values are adjusted using Bonferroni correction. The values in brackets represent the 2.5% and 97.5% confidence intervals.

Study	*N*	Effect	*p* value	Adjusted *p* value
Huang, Tse, & Cho (2014)	6488	2.5 [0.73, 4.2]	0.0059	0.16
Kay, Laurin, Fitzsimons, & Landau (2014)	6411	0.0041 [-0.012, 0.02]	0.61	1
Alter, Oppenheimer, Epley, & Eyre (2007)	7091	0.0016 [-0.0042, 0.0074]	0.59	1
Graham, Haidt, & Nosek (2009)	7181	0.16 [0.0032, 0.32]	0.046	1
Rottenstreich & Hsee (2001)	7136	-0.013 [-0.028, 0.0012]	0.071	1
Bauer, Wilkie, Kim, & Bodenhausen (2012)	6574	0.0054 [-0.0058, 0.017]	0.34	1
Miyamoto & Kitayama (2002)	7116	-0.0026 [-0.082, 0.077]	0.95	1
Inbar, Pizarro, Knobe, & Bloom (2009)	7036	-0.021 [-0.16, 0.12]	0.76	1
Critcher & Gilovich (2008)	6708	0.053 [-0.19, 0.3]	0.67	1
Van Lange, Otten, De Bruin, & Joireman (1997)	6115	0.1 [-0.045, 0.25]	0.17	1
Hauser et al. 1/1 (2007)	7121	-0.0014 [-0.21, 0.21]	0.99	1
Anderson, Kraus, Galinsky, & Keltner (2012)	7156	-0.0023 [-0.0071, 0.0025]	0.34	1
Ross, Greene, & House (1977)	6925	0.083 [-0.13, 0.29]	0.43	1
Ross et al. (1977)	7355	-0.013 [-0.27, 0.24]	0.92	1
Giessner & Schubert (2007)	7842	-0.0033 [-0.016, 0.009]	0.59	1
Tversky & Kahneman (1981)	7179	-0.0086 [-0.029, 0.012]	0.4	1
Hauser et al. 2/2 (2007)	7869	2.2e-05 [-0.018, 0.018]	1	1
Risen & Gilovich (2008)	7952	0.0053 [-0.014, 0.025]	0.59	1
Savani, Markus, Naidu, Kumar, & Berlia (2010)	7953	0.11 [0.015, 0.21]	0.025	0.67
Norenzayan, Smith, Kim, & Nisbett (2002)	7968	-0.78 [-1, -0.53]	8.9e-08	2.4e-06
Hsee (1998)	7642	0.012 [0.00037, 0.024]	0.044	1
Gray & Wegner (2009)	7953	-0.043 [-0.073, -0.013]	0.0052	0.14
Zhong & Liljenquist (2006)	6938	0.0068 [-0.0062, 0.02]	0.3	1
Schwarz, Strack, & Mai (1991)	7413	0.043 [-0.071, 0.16]	0.45	1
Shafir (1993)	7852	0.0089 [-0.012, 0.03]	0.39	1
Knobe (2003)	7933	-0.091 [-0.13, -0.056]	2.2e-06	6e-05
Tversky & Gati (1978)	3003	-0.0023 [-0.014, 0.009]	0.69	1

Supplementary Table 22. **Analysis B1**: Mixed effect model, where participants are clustered by source country and WEIRDness is operationalizated using the binary WEIRD score used by Klein et al. (2018). A *minimum of 36 participants per country* is used as an inclusion criteria. *p* values are adjusted using Bonferroni correction. The values in brackets represent the 2.5% and 97.5% confidence intervals.

Study	*N*	Effect	*p* value	Adjusted *p* value
Huang, Tse, & Cho (2014)	6537	2.5 [0.78, 4.2]	0.005	0.13
Kay, Laurin, Fitzsimons, & Landau (2014)	6455	0.0048 [-0.011, 0.02]	0.55	1
Alter, Oppenheimer, Epley, & Eyre (2007)	7135	0.0023 [-0.0039, 0.0085]	0.46	1
Graham, Haidt, & Nosek (2009)	7261	0.15 [-9e-04, 0.31]	0.051	1
Rottenstreich & Hsee (2001)	7216	-0.012 [-0.026, 0.0027]	0.11	1
Bauer, Wilkie, Kim, & Bodenhausen (2012)	6654	0.0043 [-0.0074, 0.016]	0.46	1
Miyamoto & Kitayama (2002)	7160	-0.00084 [-0.08, 0.078]	0.98	1
Inbar, Pizarro, Knobe, & Bloom (2009)	7080	-0.019 [-0.16, 0.12]	0.78	1
Critcher & Gilovich (2008)	6752	0.045 [-0.2, 0.29]	0.72	1
Van Lange, Otten, De Bruin, & Joireman (1997)	6206	0.093 [-0.05, 0.24]	0.2	1
Hauser et al. 1/1 (2007)	7201	-0.0039 [-0.21, 0.2]	0.97	1
Anderson, Kraus, Galinsky, & Keltner (2012)	7236	-0.0026 [-0.0074, 0.0022]	0.28	1
Ross, Greene, & House (1977)	6968	0.069 [-0.14, 0.28]	0.52	1
Ross et al. (1977)	7440	-0.014 [-0.25, 0.23]	0.91	1
Giessner & Schubert (2007)	7889	-0.0032 [-0.015, 0.0088]	0.59	1
Tversky & Kahneman (1981)	7227	-0.0058 [-0.026, 0.014]	0.56	1
Hauser et al. 2/2 (2007)	7917	-0.00022 [-0.017, 0.017]	0.98	1
Risen & Gilovich (2008)	7999	0.008 [-0.011, 0.027]	0.4	1
Savani, Markus, Naidu, Kumar, & Berlia (2010)	8001	0.1 [0.0069, 0.2]	0.036	0.97
Norenzayan, Smith, Kim, & Nisbett (2002)	8016	-0.81 [-1.1, -0.56]	2.1e-08	5.7e-07
Hsee (1998)	7690	0.0066 [-0.0071, 0.02]	0.34	1
Gray & Wegner (2009)	8001	-0.04 [-0.069, -0.01]	0.0092	0.25
Zhong & Liljenquist (2006)	7022	0.0041 [-0.008, 0.016]	0.5	1
Schwarz, Strack, & Mai (1991)	7459	0.038 [-0.074, 0.15]	0.5	1
Shafir (1993)	7900	0.0066 [-0.014, 0.027]	0.52	1
Knobe (2003)	7981	-0.093 [-0.13, -0.059]	8.6e-07	2.3e-05
Tversky & Gati (1978)	3381	-0.0058 [-0.016, 0.0045]	0.27	1

Supplementary Table 23. **Analysis B1**: Mixed effect model, where participants are clustered by source country and WEIRDness is operationalizated using the binary WEIRD score used by Klein et al. (2018). A *minimum of 10 participants per country* is used as an inclusion criteria. *p* values are adjusted using Bonferroni correction. The values in brackets represent the 2.5% and 97.5% confidence intervals.

Study	*N*	Effect	*p* value	Adjusted *p* value
Huang, Tse, & Cho (2014)	6587	2.4 [0.63, 4.1]	0.0081	0.22
Kay, Laurin, Fitzsimons, & Landau (2014)	6490	0.0044 [-0.011, 0.02]	0.57	1
Alter, Oppenheimer, Epley, & Eyre (2007)	7170	0.0016 [-0.0049, 0.0081]	0.63	1
Graham, Haidt, & Nosek (2009)	7261	0.15 [-0.00091, 0.31]	0.051	1
Rottenstreich & Hsee (2001)	7216	-0.012 [-0.026, 0.0027]	0.11	1
Bauer, Wilkie, Kim, & Bodenhausen (2012)	6654	0.0043 [-0.0074, 0.016]	0.46	1
Miyamoto & Kitayama (2002)	7195	-0.0017 [-0.08, 0.077]	0.97	1
Inbar, Pizarro, Knobe, & Bloom (2009)	7115	-0.018 [-0.15, 0.12]	0.79	1
Critcher & Gilovich (2008)	6785	0.044 [-0.2, 0.29]	0.72	1
Van Lange, Otten, De Bruin, & Joireman (1997)	6239	0.092 [-0.05, 0.23]	0.2	1
Hauser et al. 1/1 (2007)	7201	-0.0039 [-0.21, 0.21]	0.97	1
Anderson, Kraus, Galinsky, & Keltner (2012)	7236	-0.0026 [-0.0074, 0.0022]	0.28	1
Ross, Greene, & House (1977)	7002	0.07 [-0.14, 0.28]	0.51	1
Ross et al. (1977)	7440	-0.014 [-0.26, 0.23]	0.91	1
Giessner & Schubert (2007)	7889	-0.0032 [-0.015, 0.0088]	0.59	1
Tversky & Kahneman (1981)	7227	-0.0058 [-0.026, 0.014]	0.56	1
Hauser et al. 2/2 (2007)	7917	-0.00022 [-0.017, 0.017]	0.98	1
Risen & Gilovich (2008)	7999	0.008 [-0.011, 0.027]	0.4	1
Savani, Markus, Naidu, Kumar, & Berlia (2010)	8001	0.1 [0.0069, 0.2]	0.036	0.97
Norenzayan, Smith, Kim, & Nisbett (2002)	8016	-0.81 [-1.1, -0.56]	2.1e-08	5.7e-07
Hsee (1998)	7690	0.0066 [-0.0071, 0.02]	0.34	1
Gray & Wegner (2009)	8001	-0.04 [-0.069, -0.01]	0.0092	0.25
Zhong & Liljenquist (2006)	7056	0.004 [-0.0084, 0.016]	0.52	1
Schwarz, Strack, & Mai (1991)	7459	0.038 [-0.074, 0.15]	0.5	1
Shafir (1993)	7900	0.0067 [-0.014, 0.027]	0.52	1
Knobe (2003)	7981	-0.093 [-0.13, -0.059]	8.6e-07	2.3e-05
Tversky & Gati (1978)	3549	-0.0088 [-0.018, 0.00031]	0.058	1

Supplementary Table 24. **Analysis B1**: Mixed effect model, where participants are clustered by source country and WEIRDness is operationalizated using the mean WEIRD score developed by Klein et al. (2018). A **minimum of 100 participants per country** is used as an inclusion criteria. *p* values are adjusted using Bonferroni correction. The values in brackets represent the 2.5% and 97.5% confidence intervals.

Study	*N*	Effect	*p* value	Adjusted *p* value
Huang, Tse, & Cho (2014)	5920	6.8 [3.2, 10]	0.00037	0.01
Kay, Laurin, Fitzsimons, & Landau (2014)	6014	0.0016 [-0.032, 0.035]	0.92	1
Alter, Oppenheimer, Epley, & Eyre (2007)	6696	0.0066 [-0.0057, 0.019]	0.29	1
Graham, Haidt, & Nosek (2009)	6782	0.31 [-0.045, 0.67]	0.086	1
Rottenstreich & Hsee (2001)	6739	-0.024 [-0.055, 0.0065]	0.12	1
Bauer, Wilkie, Kim, & Bodenhausen (2012)	6175	0.019 [-0.0057, 0.043]	0.13	1
Miyamoto & Kitayama (2002)	6720	-0.1 [-0.25, 0.037]	0.14	1
Inbar, Pizarro, Knobe, & Bloom (2009)	6730	-0.087 [-0.4, 0.22]	0.58	1
Critcher & Gilovich (2008)	6054	-0.13 [-0.68, 0.41]	0.63	1
Van Lange, Otten, De Bruin, & Joireman (1997)	5507	0.22 [-0.12, 0.57]	0.2	1
Hauser et al. 1/1 (2007)	6724	-0.025 [-0.51, 0.46]	0.92	1
Anderson, Kraus, Galinsky, & Keltner (2012)	6757	-0.0014 [-0.011, 0.0084]	0.78	1
Ross, Greene, & House (1977)	6348	0.3 [-0.17, 0.77]	0.2	1
Ross et al. (1977)	6454	0.12 [-0.45, 0.69]	0.67	1
Giessner & Schubert (2007)	6902	-0.019 [-0.049, 0.011]	0.2	1
Tversky & Kahneman (1981)	6531	-0.054 [-0.11, 0.00073]	0.053	1
Hauser et al. 2/2 (2007)	7223	-0.035 [-0.072, 0.0023]	0.065	1
Risen & Gilovich (2008)	7197	0.016 [-0.025, 0.058]	0.43	1
Savani, Markus, Naidu, Kumar, & Berlia (2010)	7299	0.034 [-0.033, 0.1]	0.31	1
Norenzayan, Smith, Kim, & Nisbett (2002)	7308	-1.1 [-1.6, -0.54]	0.00022	0.0061
Hsee (1998)	6993	0.034 [0.0078, 0.061]	0.012	0.34
Gray & Wegner (2009)	7294	-0.049 [-0.1, 0.0026]	0.062	1
Zhong & Liljenquist (2006)	6395	0.011 [-0.017, 0.039]	0.44	1
Schwarz, Strack, & Mai (1991)	6438	0.1 [-0.2, 0.41]	0.49	1
Shafir (1993)	7277	-0.014 [-0.06, 0.033]	0.56	1
Zaval, Keenan, Johnson, & Weber (2014)	3222	0.031 [-0.12, 0.19]	0.69	1
Knobe (2003)	7273	-0.13 [-0.2, -0.064]	3e-04	0.0083
Tversky & Gati (1978)	2600	-0.00051 [-0.023, 0.022]	0.96	1

Supplementary Table 25. **Analysis B1**: Mixed effect model, where participants are clustered by source country and WEIRDness is operationalizated using the mean WEIRD score developed by Klein et al. (2018). A *minimum of 50 participants per country* is used as an inclusion criteria. *p* values are adjusted using Bonferroni correction. The values in brackets represent the 2.5% and 97.5% confidence intervals.

Study	*N*	Effect	*p* value	Adjusted *p* value
Huang, Tse, & Cho (2014)	6488	5.5 [2, 9.1]	0.0028	0.077
Kay, Laurin, Fitzsimons, & Landau (2014)	6411	0.0065 [-0.026, 0.04]	0.69	1
Alter, Oppenheimer, Epley, & Eyre (2007)	7091	0.0055 [-0.0066, 0.018]	0.36	1
Graham, Haidt, & Nosek (2009)	7181	0.33 [0.00076, 0.65]	0.049	1
Rottenstreich & Hsee (2001)	7136	-0.032 [-0.062, -0.0026]	0.034	0.94
Bauer, Wilkie, Kim, & Bodenhausen (2012)	6574	0.014 [-0.0098, 0.037]	0.25	1
Miyamoto & Kitayama (2002)	7116	-0.043 [-0.21, 0.12]	0.61	1
Inbar, Pizarro, Knobe, & Bloom (2009)	7036	-0.067 [-0.36, 0.22]	0.64	1
Critcher & Gilovich (2008)	6708	0.023 [-0.49, 0.53]	0.93	1
Van Lange, Otten, De Bruin, & Joireman (1997)	6115	0.22 [-0.08, 0.52]	0.15	1
Hauser et al. 1/1 (2007)	7121	-0.004 [-0.44, 0.44]	0.99	1
Anderson, Kraus, Galinsky, & Keltner (2012)	7156	-0.0034 [-0.013, 0.0066]	0.5	1
Ross, Greene, & House (1977)	6925	0.26 [-0.17, 0.7]	0.23	1
Ross et al. (1977)	7355	0.014 [-0.47, 0.5]	0.95	1
Giessner & Schubert (2007)	7842	-0.011 [-0.035, 0.013]	0.38	1
Tversky & Kahneman (1981)	7179	-0.025 [-0.069, 0.018]	0.25	1
Hauser et al. 2/2 (2007)	7869	-0.009 [-0.044, 0.026]	0.61	1
Risen & Gilovich (2008)	7952	0.012 [-0.026, 0.049]	0.54	1
Savani, Markus, Naidu, Kumar, & Berlia (2010)	7953	0.18 [-0.0064, 0.37]	0.058	1
Norenzayan, Smith, Kim, & Nisbett (2002)	7968	-1.5 [-2, -1]	8.9e-08	2.5e-06
Hsee (1998)	7642	0.024 [7.8e-05, 0.047]	0.049	1
Gray & Wegner (2009)	7953	-0.084 [-0.14, -0.026]	0.0055	0.15
Zhong & Liljenquist (2006)	6938	0.0072 [-0.017, 0.032]	0.56	1
Schwarz, Strack, & Mai (1991)	7413	0.094 [-0.13, 0.32]	0.4	1
Shafir (1993)	7852	0.0012 [-0.04, 0.042]	0.95	1
Zaval, Keenan, Johnson, & Weber (2014)	4041	-0.011 [-0.03, 0.0088]	0.28	1
Knobe (2003)	7933	-0.18 [-0.25, -0.11]	2.1e-06	5.9e-05
Tversky & Gati (1978)	3003	-0.0038 [-0.023, 0.016]	0.69	1

Supplementary Table 26. **Analysis B1**: Mixed effect model, where participants are clustered by source country and WEIRDness is operationalizated using the mean WEIRD score developed by Klein et al. (2018). A *minimum of 36 participants per country* is used as an inclusion criteria. *p* values are adjusted using Bonferroni correction. The values in brackets represent the 2.5% and 97.5% confidence intervals.

Study	*N*	Effect	*p* value	Adjusted *p* value
Huang, Tse, & Cho (2014)	6537	5.6 [2.1, 9.1]	0.0024	0.067
Kay, Laurin, Fitzsimons, & Landau (2014)	6455	0.0078 [-0.025, 0.041]	0.64	1
Alter, Oppenheimer, Epley, & Eyre (2007)	7135	0.0067 [-0.0061, 0.02]	0.3	1
Graham, Haidt, & Nosek (2009)	7261	0.32 [-0.0027, 0.64]	0.052	1
Rottenstreich & Hsee (2001)	7216	-0.03 [-0.06, -0.00045]	0.047	1
Bauer, Wilkie, Kim, & Bodenhausen (2012)	6654	0.011 [-0.013, 0.036]	0.35	1
Miyamoto & Kitayama (2002)	7160	-0.04 [-0.2, 0.12]	0.63	1
Inbar, Pizarro, Knobe, & Bloom (2009)	7080	-0.064 [-0.35, 0.22]	0.66	1
Critcher & Gilovich (2008)	6752	0.01 [-0.5, 0.52]	0.97	1
Van Lange, Otten, De Bruin, & Joireman (1997)	6206	0.21 [-0.087, 0.51]	0.16	1
Hauser et al. 1/1 (2007)	7201	-0.0074 [-0.44, 0.43]	0.97	1
Anderson, Kraus, Galinsky, & Keltner (2012)	7236	-0.0038 [-0.014, 0.0062]	0.45	1
Ross, Greene, & House (1977)	6968	0.24 [-0.2, 0.68]	0.28	1
Ross et al. (1977)	7440	0.029 [-0.44, 0.5]	0.9	1
Giessner & Schubert (2007)	7889	-0.01 [-0.033, 0.013]	0.38	1
Tversky & Kahneman (1981)	7227	-0.017 [-0.059, 0.024]	0.41	1
Hauser et al. 2/2 (2007)	7917	-0.009 [-0.042, 0.024]	0.59	1
Risen & Gilovich (2008)	7999	0.017 [-0.02, 0.054]	0.35	1
Savani, Markus, Naidu, Kumar, & Berlia (2010)	8001	0.16 [-0.021, 0.35]	0.082	1
Norenzayan, Smith, Kim, & Nisbett (2002)	8016	-1.6 [-2.1, -1.1]	1.9e-08	5.4e-07
Hsee (1998)	7690	0.011 [-0.015, 0.038]	0.4	1
Gray & Wegner (2009)	8001	-0.076 [-0.13, -0.019]	0.01	0.29
Zhong & Liljenquist (2006)	7022	0.0029 [-0.02, 0.026]	0.8	1
Schwarz, Strack, & Mai (1991)	7459	0.082 [-0.14, 0.3]	0.46	1
Shafir (1993)	7900	-0.0028 [-0.043, 0.037]	0.89	1
Zaval, Keenan, Johnson, & Weber (2014)	4133	-0.011 [-0.03, 0.0075]	0.23	1
Knobe (2003)	7981	-0.18 [-0.25, -0.12]	8e-07	2.2e-05
Tversky & Gati (1978)	3381	-0.01 [-0.029, 0.0089]	0.29	1

Supplementary Table 27. **Analysis B1**: Mixed effect model, where participants are clustered by source country and WEIRDness is operationalizated using the mean WEIRD score developed by Klein et al. (2018). A *minimum of 10 participants per country* is used as an inclusion criteria. *p* values are adjusted using Bonferroni correction. The values in brackets represent the 2.5% and 97.5% confidence intervals.

Study	*N*	Effect	*p* value	Adjusted *p* value
Huang, Tse, & Cho (2014)	6587	5.4 [1.9, 9]	0.0034	0.095
Kay, Laurin, Fitzsimons, & Landau (2014)	6490	0.0075 [-0.025, 0.04]	0.65	1
Alter, Oppenheimer, Epley, & Eyre (2007)	7170	0.0062 [-0.0074, 0.02]	0.37	1
Graham, Haidt, & Nosek (2009)	7261	0.32 [-0.0027, 0.65]	0.052	1
Rottenstreich & Hsee (2001)	7216	-0.031 [-0.061, -0.00045]	0.047	1
Bauer, Wilkie, Kim, & Bodenhausen (2012)	6654	0.011 [-0.013, 0.036]	0.35	1
Miyamoto & Kitayama (2002)	7195	-0.041 [-0.2, 0.12]	0.62	1
Inbar, Pizarro, Knobe, & Bloom (2009)	7115	-0.063 [-0.35, 0.22]	0.66	1
Critcher & Gilovich (2008)	6785	0.009 [-0.5, 0.52]	0.97	1
Van Lange, Otten, De Bruin, & Joireman (1997)	6239	0.21 [-0.085, 0.51]	0.16	1
Hauser et al. 1/1 (2007)	7201	-0.0075 [-0.44, 0.43]	0.97	1
Anderson, Kraus, Galinsky, & Keltner (2012)	7236	-0.0038 [-0.014, 0.0062]	0.45	1
Ross, Greene, & House (1977)	7002	0.24 [-0.2, 0.68]	0.28	1
Ross et al. (1977)	7440	0.029 [-0.44, 0.5]	0.9	1
Giessner & Schubert (2007)	7889	-0.01 [-0.034, 0.013]	0.38	1
Tversky & Kahneman (1981)	7227	-0.018 [-0.06, 0.025]	0.41	1
Hauser et al. 2/2 (2007)	7917	-0.0091 [-0.043, 0.025]	0.59	1
Risen & Gilovich (2008)	7999	0.017 [-0.02, 0.055]	0.35	1
Savani, Markus, Naidu, Kumar, & Berlia (2010)	8001	0.16 [-0.021, 0.35]	0.082	1
Norenzayan, Smith, Kim, & Nisbett (2002)	8016	-1.6 [-2.1, -1.1]	1.9e-08	5.4e-07
Hsee (1998)	7690	0.011 [-0.015, 0.038]	0.4	1
Gray & Wegner (2009)	8001	-0.076 [-0.13, -0.019]	0.01	0.29
Zhong & Liljenquist (2006)	7056	0.0032 [-0.02, 0.027]	0.79	1
Schwarz, Strack, & Mai (1991)	7459	0.082 [-0.14, 0.3]	0.46	1
Shafir (1993)	7900	-0.0028 [-0.043, 0.037]	0.89	1
Zaval, Keenan, Johnson, & Weber (2014)	4202	-0.012 [-0.03, 0.005]	0.16	1
Knobe (2003)	7981	-0.18 [-0.25, -0.12]	8e-07	2.2e-05
Tversky & Gati (1978)	3549	-0.016 [-0.034, 0.0019]	0.08	1

Supplementary Table 28. **Analysis B2**: Mixed effect model, where participants are clustered by birth country and participants missing birth countries are excluded from analysis. WEIRDness is operationalizated using CF_{ST}, where countries missing CF_{ST} are excluded from analysis. A *minimum of 100 participants per country* is used as an inclusion criteria. *p* values are adjusted using Bonferroni correction. The values in brackets represent the 2.5% and 97.5% confidence intervals.

Study	*N*	Effect	*p* value	Adjusted *p* value
Huang, Tse, & Cho (2014)	5210	-23 [-61, 16]	0.23	1
Kay, Laurin, Fitzsimons, & Landau (2014)	5277	0.0049 [-0.27, 0.28]	0.97	1
Alter, Oppenheimer, Epley, & Eyre (2007)	5817	-0.061 [-0.16, 0.038]	0.21	1
Graham, Haidt, & Nosek (2009)	5870	-2 [-3.6, -0.3]	0.024	0.66
Rottenstreich & Hsee (2001)	5856	-0.085 [-0.49, 0.32]	0.66	1
Bauer, Wilkie, Kim, & Bodenhausen (2012)	5405	0.071 [-0.15, 0.29]	0.51	1
Miyamoto & Kitayama (2002)	5844	0.54 [-0.8, 1.9]	0.41	1
Inbar, Pizarro, Knobe, & Bloom (2009)	5839	0.24 [-0.94, 1.4]	0.67	1
Critcher & Gilovich (2008)	5224	2.1 [-0.51, 4.7]	0.11	1
Van Lange, Otten, De Bruin, & Joireman (1997)	4792	0.0023 [-0.9, 0.91]	1	1
Hauser et al. 1/1 (2007)	5748	-0.39 [-1.2, 0.39]	0.31	1
Anderson, Kraus, Galinsky, & Keltner (2012)	5869	-0.044 [-0.12, 0.034]	0.25	1
Ross, Greene, & House (1977)	5595	-1.1 [-4.4, 2.1]	0.48	1
Ross et al. (1977)	5139	-1.5 [-6.5, 3.5]	0.52	1
Giessner & Schubert (2007)	5591	-0.061 [-0.35, 0.23]	0.65	1
Tversky & Kahneman (1981)	4843	-0.036 [-0.43, 0.36]	0.84	1
Hauser et al. 2/2 (2007)	5613	0.16 [-0.19, 0.51]	0.34	1
Risen & Gilovich (2008)	5655	0.16 [-0.25, 0.58]	0.4	1
Savani, Markus, Naidu, Kumar, & Berlia (2010)	5664	-0.19 [-0.48, 0.11]	0.19	1
Norenzayan, Smith, Kim, & Nisbett (2002)	5661	6.3 [0.26, 12]	0.042	1
Hsee (1998)	5460	0.19 [-0.13, 0.51]	0.21	1
Gray & Wegner (2009)	5653	0.18 [-0.41, 0.77]	0.52	1
Zhong & Liljenquist (2006)	5228	-0.013 [-0.17, 0.15]	0.86	1
Schwarz, Strack, & Mai (1991)	5337	-0.71 [-1.9, 0.52]	0.23	1
Shafir (1993)	5643	0.07 [-0.28, 0.42]	0.67	1
Zaval, Keenan, Johnson, & Weber (2014)	2602	0.075 [-0.19, 0.34]	0.48	1
Knobe (2003)	5638	0.42 [-0.44, 1.3]	0.31	1
Tversky & Gati (1978)	2015	0.0052 [-0.11, 0.12]	0.89	1

Supplementary Table 29. **Analysis B2**: Mixed effect model, where participants are clustered by birth country and participants missing birth countries are excluded from analysis. WEIRDness is operationalizated using CF_{ST}, where countries missing CF_{ST} are excluded from analysis. A *minimum of 50 participants per country* is used as an inclusion criteria. *p* values are adjusted using Bonferroni correction. The values in brackets represent the 2.5% and 97.5% confidence intervals.

Study	*N*	Effect	*p* value	Adjusted *p* value
Huang, Tse, & Cho (2014)	5674	-27 [-65, 12]	0.17	1
Kay, Laurin, Fitzsimons, & Landau (2014)	5595	-0.026 [-0.26, 0.21]	0.82	1
Alter, Oppenheimer, Epley, & Eyre (2007)	6137	-0.073 [-0.19, 0.039]	0.19	1
Graham, Haidt, & Nosek (2009)	6191	-1.5 [-2.8, -0.17]	0.029	0.81
Rottenstreich & Hsee (2001)	6175	-0.048 [-0.37, 0.27]	0.76	1
Bauer, Wilkie, Kim, & Bodenhausen (2012)	5724	0.044 [-0.15, 0.24]	0.64	1
Miyamoto & Kitayama (2002)	6162	0.62 [-0.49, 1.7]	0.26	1
Inbar, Pizarro, Knobe, & Bloom (2009)	6158	0.3 [-0.7, 1.3]	0.54	1
Critcher & Gilovich (2008)	5807	2 [-0.49, 4.6]	0.11	1
Van Lange, Otten, De Bruin, & Joireman (1997)	5382	0.03 [-0.72, 0.78]	0.93	1
Hauser et al. 1/1 (2007)	6166	-0.38 [-1, 0.24]	0.22	1
Anderson, Kraus, Galinsky, & Keltner (2012)	6190	-0.055 [-0.14, 0.03]	0.19	1
Ross, Greene, & House (1977)	6005	-1.8 [-5, 1.4]	0.26	1
Ross et al. (1977)	5966	0.26 [-4, 4.5]	0.9	1
Giessner & Schubert (2007)	6259	0.00051 [-0.21, 0.21]	1	1
Tversky & Kahneman (1981)	5620	-0.044 [-0.42, 0.33]	0.81	1
Hauser et al. 2/2 (2007)	6289	0.34 [-0.037, 0.72]	0.074	1
Risen & Gilovich (2008)	6336	-0.074 [-0.54, 0.39]	0.74	1
Savani, Markus, Naidu, Kumar, & Berlia (2010)	6345	-0.18 [-0.56, 0.2]	0.34	1
Norenzayan, Smith, Kim, & Nisbett (2002)	6344	6.3 [0.074, 13]	0.048	1
Hsee (1998)	6137	0.16 [-0.12, 0.44]	0.24	1
Gray & Wegner (2009)	6333	0.073 [-0.33, 0.47]	0.71	1
Zhong & Liljenquist (2006)	5846	-0.098 [-0.32, 0.12]	0.36	1
Schwarz, Strack, & Mai (1991)	5961	-0.26 [-1.3, 0.77]	0.6	1
Shafir (1993)	6226	-0.015 [-0.35, 0.32]	0.93	1
Zaval, Keenan, Johnson, & Weber (2014)	3165	0.088 [-0.07, 0.25]	0.25	1
Knobe (2003)	6321	0.56 [-0.16, 1.3]	0.12	1
Tversky & Gati (1978)	2384	0.0091 [-0.086, 0.1]	0.83	1

Supplementary Table 30. **Analysis B2**: Mixed effect model, where participants are clustered by birth country and participants missing birth countries are excluded from analysis. WEIRDness is operationalizated using CF_{ST}, where countries missing CF_{ST} are excluded from analysis. A *minimum of 36 participants per country* is used as an inclusion criteria. *p* values are adjusted using Bonferroni correction. The values in brackets represent the 2.5% and 97.5% confidence intervals.

Study	*N*	Effect	*p* value	Adjusted *p* value
Huang, Tse, & Cho (2014)	5714	-27 [-66, 12]	0.16	1
Kay, Laurin, Fitzsimons, & Landau (2014)	5728	-0.063 [-0.35, 0.22]	0.65	1
Alter, Oppenheimer, Epley, & Eyre (2007)	6270	-0.067 [-0.28, 0.15]	0.52	1
Graham, Haidt, & Nosek (2009)	6325	-1.5 [-2.9, -0.13]	0.034	0.94
Rottenstreich & Hsee (2001)	6309	-0.068 [-0.41, 0.28]	0.69	1
Bauer, Wilkie, Kim, & Bodenhausen (2012)	5858	0.053 [-0.17, 0.28]	0.63	1
Miyamoto & Kitayama (2002)	6296	0.64 [-0.53, 1.8]	0.27	1
Inbar, Pizarro, Knobe, & Bloom (2009)	6292	0.18 [-0.96, 1.3]	0.75	1
Critcher & Gilovich (2008)	5938	2.3 [-1, 5.7]	0.16	1
Van Lange, Otten, De Bruin, & Joireman (1997)	5511	0.071 [-0.82, 0.96]	0.87	1
Hauser et al. 1/1 (2007)	6299	-0.34 [-1, 0.33]	0.3	1
Anderson, Kraus, Galinsky, & Keltner (2012)	6324	-0.039 [-0.16, 0.08]	0.5	1
Ross, Greene, & House (1977)	6137	-2.4 [-7.2, 2.5]	0.32	1
Ross et al. (1977)	5966	0.26 [-4.1, 4.6]	0.9	1
Giessner & Schubert (2007)	6295	-0.039 [-0.24, 0.17]	0.7	1
Tversky & Kahneman (1981)	5657	-0.14 [-0.53, 0.25]	0.46	1
Hauser et al. 2/2 (2007)	6325	0.36 [0.0049, 0.72]	0.047	1
Risen & Gilovich (2008)	6373	-0.13 [-0.58, 0.32]	0.55	1
Savani, Markus, Naidu, Kumar, & Berlia (2010)	6382	1.8 [-1.2, 4.9]	0.23	1
Norenzayan, Smith, Kim, & Nisbett (2002)	6382	5.8 [-0.19, 12]	0.057	1
Hsee (1998)	6174	0.19 [-0.08, 0.46]	0.16	1
Gray & Wegner (2009)	6371	0.068 [-0.31, 0.45]	0.71	1
Zhong & Liljenquist (2006)	5846	-0.1 [-0.32, 0.12]	0.36	1
Schwarz, Strack, & Mai (1991)	5998	-0.41 [-1.4, 0.59]	0.4	1
Shafir (1993)	6262	0.17 [-0.27, 0.61]	0.42	1
Zaval, Keenan, Johnson, & Weber (2014)	3302	0.089 [-0.042, 0.22]	0.17	1
Knobe (2003)	6359	0.69 [-0.019, 1.4]	0.056	1
Tversky & Gati (1978)	2719	0.052 [-0.14, 0.25]	0.58	1

Supplementary Table 31. **Analysis B2**: Mixed effect model, where participants are clustered by birth country and participants missing birth countries are excluded from analysis. WEIRDness is operationalizated using CF_{ST}, where countries missing CF_{ST} are excluded from analysis. A *minimum of 10 participants per country* is used as an inclusion criteria. *p* values are adjusted using Bonferroni correction. The values in brackets represent the 2.5% and 97.5% confidence intervals.

Study	*N*	Effect	*p* value	Adjusted *p* value
Huang, Tse, & Cho (2014)	5859	10 [-19, 40]	0.48	1
Kay, Laurin, Fitzsimons, & Landau (2014)	5860	-0.13 [-0.4, 0.14]	0.34	1
Alter, Oppenheimer, Epley, & Eyre (2007)	6402	-0.017 [-0.24, 0.2]	0.88	1
Graham, Haidt, & Nosek (2009)	6457	-0.82 [-1.7, 0.024]	0.056	1
Rottenstreich & Hsee (2001)	6441	0.13 [-0.15, 0.4]	0.35	1
Bauer, Wilkie, Kim, & Bodenhausen (2012)	5990	0.26 [0.0077, 0.51]	0.044	1
Miyamoto & Kitayama (2002)	6428	-0.82 [-2.2, 0.53]	0.22	1
Inbar, Pizarro, Knobe, & Bloom (2009)	6381	0.11 [-0.69, 0.92]	0.78	1
Critcher & Gilovich (2008)	6046	-2.6 [-6.6, 1.3]	0.18	1
Van Lange, Otten, De Bruin, & Joireman (1997)	5629	0.017 [-0.5, 0.53]	0.95	1
Hauser et al. 1/1 (2007)	6430	3.2 [0.87, 5.6]	0.0089	0.25
Anderson, Kraus, Galinsky, & Keltner (2012)	6456	-0.1 [-0.2, -0.00068]	0.049	1
Ross, Greene, & House (1977)	6256	-2.4 [-5.7, 0.89]	0.15	1
Ross et al. (1977)	6306	1.4 [-2.6, 5.3]	0.48	1
Giessner & Schubert (2007)	6641	-0.22 [-0.49, 0.051]	0.11	1
Tversky & Kahneman (1981)	6000	-1.5 [-3.7, 0.66]	0.17	1
Hauser et al. 2/2 (2007)	6668	1 [-0.96, 3]	0.3	1
Risen & Gilovich (2008)	6722	-0.059 [-0.48, 0.36]	0.78	1
Savani, Markus, Naidu, Kumar, & Berlia (2010)	6722	1.9 [-1.9, 5.7]	0.31	1
Norenzayan, Smith, Kim, & Nisbett (2002)	6732	4.7 [0.43, 9.1]	0.032	0.9
Hsee (1998)	6537	0.17 [-0.068, 0.41]	0.16	1
Gray & Wegner (2009)	6720	0.14 [-0.23, 0.52]	0.45	1
Zhong & Liljenquist (2006)	6173	0.0097 [-0.25, 0.26]	0.94	1
Schwarz, Strack, & Mai (1991)	6303	-0.17 [-0.73, 0.39]	0.54	1
Shafir (1993)	6630	0.36 [-1.4, 2.1]	0.67	1
Zaval, Keenan, Johnson, & Weber (2014)	3560	0.12 [-0.095, 0.33]	0.27	1
Knobe (2003)	6707	0.62 [0.1, 1.1]	0.02	0.56
Tversky & Gati (1978)	2911	0.099 [-0.058, 0.26]	0.21	1

Supplementary Table 32. **Analysis B2**: Mixed effect model, where participants are clustered by birth country and participants missing birth countries are excluded from analysis. WEIRDness is operationalizated using CF_{ST}, imputing the CF_{ST} for countries missing cultural distance. A *minimum of 100 participants per country* is used as an inclusion criteria. *p* values are adjusted using Bonferroni correction. The values in brackets represent the 2.5% and 97.5% confidence intervals.

Study	*N*	Effect	*p* value	Adjusted *p* value
Huang, Tse, & Cho (2014)	5310	-24 [-62, 13]	0.19	1
Kay, Laurin, Fitzsimons, & Landau (2014)	5377	0.00038 [-0.24, 0.24]	1	1
Alter, Oppenheimer, Epley, & Eyre (2007)	6021	-0.019 [-0.13, 0.088]	0.71	1
Graham, Haidt, & Nosek (2009)	6076	-1.5 [-3, 0.056]	0.058	1
Rottenstreich & Hsee (2001)	6061	-0.18 [-0.56, 0.2]	0.32	1
Bauer, Wilkie, Kim, & Bodenhausen (2012)	5507	-0.035 [-0.27, 0.2]	0.76	1
Miyamoto & Kitayama (2002)	6048	0.53 [-0.72, 1.8]	0.39	1
Inbar, Pizarro, Knobe, & Bloom (2009)	6044	0.2 [-0.84, 1.2]	0.69	1
Critcher & Gilovich (2008)	5224	2.1 [-0.51, 4.6]	0.11	1
Van Lange, Otten, De Bruin, & Joireman (1997)	4792	0.0023 [-0.9, 0.91]	1	1
Hauser et al. 1/1 (2007)	5950	-0.34 [-1, 0.33]	0.3	1
Anderson, Kraus, Galinsky, & Keltner (2012)	6074	-0.016 [-0.092, 0.06]	0.66	1
Ross, Greene, & House (1977)	5696	-1.4 [-4.8, 2]	0.41	1
Ross et al. (1977)	5304	0.82 [-4.7, 6.4]	0.75	1
Giessner & Schubert (2007)	5772	-0.057 [-0.31, 0.19]	0.63	1
Tversky & Kahneman (1981)	5029	-0.0031 [-0.34, 0.34]	0.98	1
Hauser et al. 2/2 (2007)	5797	0.11 [-0.2, 0.42]	0.46	1
Risen & Gilovich (2008)	5843	0.057 [-0.33, 0.45]	0.76	1
Savani, Markus, Naidu, Kumar, & Berlia (2010)	5852	-0.2 [-0.45, 0.06]	0.12	1
Norenzayan, Smith, Kim, & Nisbett (2002)	5849	4.8 [-0.96, 11]	0.095	1
Hsee (1998)	5647	0.071 [-0.26, 0.4]	0.64	1
Gray & Wegner (2009)	5840	0.29 [-0.24, 0.83]	0.26	1
Zhong & Liljenquist (2006)	5374	-0.067 [-0.23, 0.094]	0.38	1
Schwarz, Strack, & Mai (1991)	5515	-0.68 [-1.8, 0.39]	0.19	1
Shafir (1993)	5831	-0.097 [-0.49, 0.29]	0.6	1
Zaval, Keenan, Johnson, & Weber (2014)	2602	0.074 [-0.19, 0.34]	0.48	1
Knobe (2003)	5825	0.49 [-0.27, 1.2]	0.18	1
Tversky & Gati (1978)	2015	0.0052 [-0.11, 0.12]	0.89	1

Supplementary Table 33. **Analysis B2**: Mixed effect model, where participants are clustered by birth country and participants missing birth countries are excluded from analysis. WEIRDness is operationalizated using CF_{ST}, imputing the CF_{ST} for countries missing cultural distance. A *minimum of 50 participants per country* is used as an inclusion criteria. *p* values are adjusted using Bonferroni correction. The values in brackets represent the 2.5% and 97.5% confidence intervals.

Study	*N*	Effect	*p* value	Adjusted *p* value
Huang, Tse, & Cho (2014)	5929	-24 [-59, 10]	0.16	1
Kay, Laurin, Fitzsimons, & Landau (2014)	5759	-0.026 [-0.23, 0.18]	0.79	1
Alter, Oppenheimer, Epley, & Eyre (2007)	6406	-0.038 [-0.15, 0.077]	0.5	1
Graham, Haidt, & Nosek (2009)	6463	-1.1 [-2.3, 0.091]	0.068	1
Rottenstreich & Hsee (2001)	6446	-0.12 [-0.43, 0.18]	0.41	1
Bauer, Wilkie, Kim, & Bodenhausen (2012)	5892	-0.045 [-0.25, 0.16]	0.65	1
Miyamoto & Kitayama (2002)	6432	0.61 [-0.46, 1.7]	0.25	1
Inbar, Pizarro, Knobe, & Bloom (2009)	6429	0.26 [-0.63, 1.2]	0.55	1
Critcher & Gilovich (2008)	6060	1.3 [-2.1, 4.7]	0.43	1
Van Lange, Otten, De Bruin, & Joireman (1997)	5540	0.096 [-0.62, 0.81]	0.78	1
Hauser et al. 1/1 (2007)	6433	-0.33 [-0.87, 0.22]	0.23	1
Anderson, Kraus, Galinsky, & Keltner (2012)	6461	-0.035 [-0.13, 0.056]	0.43	1
Ross, Greene, & House (1977)	6263	-2.6 [-5.6, 0.41]	0.087	1
Ross et al. (1977)	6357	1.6 [-2.5, 5.8]	0.42	1
Giessner & Schubert (2007)	6675	0.004 [-0.21, 0.22]	0.97	1
Tversky & Kahneman (1981)	6040	-0.05 [-0.37, 0.27]	0.75	1
Hauser et al. 2/2 (2007)	6708	0.25 [-0.078, 0.59]	0.13	1
Risen & Gilovich (2008)	6763	-0.089 [-0.51, 0.33]	0.67	1
Savani, Markus, Naidu, Kumar, & Berlia (2010)	6772	-0.23 [-0.64, 0.19]	0.27	1
Norenzayan, Smith, Kim, & Nisbett (2002)	6770	5.5 [-0.33, 11]	0.063	1
Hsee (1998)	6469	0.12 [-0.19, 0.44]	0.43	1
Gray & Wegner (2009)	6758	0.13 [-0.26, 0.52]	0.5	1
Zhong & Liljenquist (2006)	6226	-0.15 [-0.37, 0.058]	0.15	1
Schwarz, Strack, & Mai (1991)	6292	-0.25 [-1.2, 0.67]	0.58	1
Shafir (1993)	6650	-0.094 [-0.41, 0.22]	0.54	1
Zaval, Keenan, Johnson, & Weber (2014)	3343	0.1 [-0.034, 0.24]	0.13	1
Knobe (2003)	6747	0.54 [-0.078, 1.2]	0.084	1
Tversky & Gati (1978)	2470	0.044 [-0.054, 0.14]	0.34	1

Supplementary Table 34. **Analysis B2**: Mixed effect model, where participants are clustered by birth country and participants missing birth countries are excluded from analysis. WEIRDness is operationalizated using CF_{ST}, imputing the CF_{ST} for countries missing cultural distance. A *minimum of 36 participants per country* is used as an inclusion criteria. *p* values are adjusted using Bonferroni correction. The values in brackets represent the 2.5% and 97.5% confidence intervals.

Study	*N*	Effect	*p* value	Adjusted *p* value
Huang, Tse, & Cho (2014)	6011	-23 [-57, 10]	0.17	1
Kay, Laurin, Fitzsimons, & Landau (2014)	5935	-0.03 [-0.29, 0.23]	0.81	1
Alter, Oppenheimer, Epley, & Eyre (2007)	6583	-0.057 [-0.26, 0.15]	0.57	1
Graham, Haidt, & Nosek (2009)	6641	-1.2 [-2.5, 0.055]	0.06	1
Rottenstreich & Hsee (2001)	6623	-0.15 [-0.47, 0.17]	0.34	1
Bauer, Wilkie, Kim, & Bodenhausen (2012)	6069	-0.018 [-0.24, 0.2]	0.86	1
Miyamoto & Kitayama (2002)	6609	0.79 [-0.35, 1.9]	0.17	1
Inbar, Pizarro, Knobe, & Bloom (2009)	6563	0.12 [-0.89, 1.1]	0.81	1
Critcher & Gilovich (2008)	6232	1.9 [-2, 5.9]	0.32	1
Van Lange, Otten, De Bruin, & Joireman (1997)	5711	0.16 [-0.64, 0.96]	0.69	1
Hauser et al. 1/1 (2007)	6610	-0.21 [-0.81, 0.38]	0.47	1
Anderson, Kraus, Galinsky, & Keltner (2012)	6639	-0.035 [-0.15, 0.083]	0.55	1
Ross, Greene, & House (1977)	6435	-3.1 [-7.3, 1.2]	0.15	1
Ross et al. (1977)	6357	1.7 [-2.6, 6]	0.42	1
Giessner & Schubert (2007)	6711	-0.027 [-0.24, 0.19]	0.8	1
Tversky & Kahneman (1981)	6077	-0.13 [-0.46, 0.21]	0.44	1
Hauser et al. 2/2 (2007)	6744	0.28 [-0.041, 0.6]	0.085	1
Risen & Gilovich (2008)	6800	-0.14 [-0.55, 0.28]	0.5	1
Savani, Markus, Naidu, Kumar, & Berlia (2010)	6809	1.4 [-1.3, 4.1]	0.29	1
Norenzayan, Smith, Kim, & Nisbett (2002)	6808	5.2 [-0.44, 11]	0.069	1
Hsee (1998)	6506	0.15 [-0.16, 0.45]	0.33	1
Gray & Wegner (2009)	6796	0.12 [-0.25, 0.5]	0.51	1
Zhong & Liljenquist (2006)	6226	-0.16 [-0.37, 0.059]	0.15	1
Schwarz, Strack, & Mai (1991)	6372	-0.32 [-1.2, 0.56]	0.46	1
Shafir (1993)	6686	0.062 [-0.34, 0.46]	0.75	1
Zaval, Keenan, Johnson, & Weber (2014)	3480	0.1 [-0.019, 0.22]	0.094	1
Knobe (2003)	6785	0.65 [0.031, 1.3]	0.04	1
Tversky & Gati (1978)	2881	0.071 [-0.096, 0.24]	0.38	1

Supplementary Table 35. **Analysis B2**: Mixed effect model, where participants are clustered by birth country and participants missing birth countries are excluded from analysis. WEIRDness is operationalizated using CF_{ST}, imputing the CF_{ST} for countries missing cultural distance. A *minimum of 10 participants per country*is used as an inclusion criteria. *p* values are adjusted using Bonferroni correction. The values in brackets represent the 2.5% and 97.5% confidence intervals.

Study	*N*	Effect	*p* value	Adjusted *p* value
Huang, Tse, & Cho (2014)	6187	7.7 [-19, 35]	0.57	1
Kay, Laurin, Fitzsimons, & Landau (2014)	6100	-0.11 [-0.36, 0.15]	0.4	1
Alter, Oppenheimer, Epley, & Eyre (2007)	6747	-0.016 [-0.22, 0.19]	0.87	1
Graham, Haidt, & Nosek (2009)	6806	-0.7 [-1.5, 0.095]	0.082	1
Rottenstreich & Hsee (2001)	6788	0.058 [-0.21, 0.33]	0.66	1
Bauer, Wilkie, Kim, & Bodenhausen (2012)	6234	0.21 [-0.036, 0.45]	0.093	1
Miyamoto & Kitayama (2002)	6773	-0.71 [-2, 0.58]	0.27	1
Inbar, Pizarro, Knobe, & Bloom (2009)	6685	0.098 [-0.64, 0.84]	0.79	1
Critcher & Gilovich (2008)	6371	-2.3 [-6.3, 1.6]	0.23	1
Van Lange, Otten, De Bruin, & Joireman (1997)	5859	0.063 [-0.42, 0.55]	0.79	1
Hauser et al. 1/1 (2007)	6774	3 [0.8, 5.2]	0.0089	0.25
Anderson, Kraus, Galinsky, & Keltner (2012)	6804	-0.091 [-0.19, 0.0075]	0.069	1
Ross, Greene, & House (1977)	6585	-2.8 [-5.9, 0.22]	0.068	1
Ross et al. (1977)	6697	1.6 [-2.2, 5.4]	0.4	1
Giessner & Schubert (2007)	7057	-0.2 [-0.46, 0.057]	0.12	1
Tversky & Kahneman (1981)	6420	-1.4 [-3.5, 0.64]	0.17	1
Hauser et al. 2/2 (2007)	7087	1 [-0.86, 2.9]	0.28	1
Risen & Gilovich (2008)	7149	-0.086 [-0.48, 0.31]	0.67	1
Savani, Markus, Naidu, Kumar, & Berlia (2010)	7149	1.8 [-1.8, 5.3]	0.32	1
Norenzayan, Smith, Kim, & Nisbett (2002)	7158	4.7 [0.44, 8.9]	0.031	0.88
Hsee (1998)	6869	0.15 [-0.1, 0.39]	0.24	1
Gray & Wegner (2009)	7145	0.16 [-0.2, 0.53]	0.37	1
Zhong & Liljenquist (2006)	6553	-0.021 [-0.27, 0.22]	0.86	1
Schwarz, Strack, & Mai (1991)	6677	-0.15 [-0.68, 0.39]	0.59	1
Shafir (1993)	7054	0.34 [-1.3, 1.9]	0.67	1
Zaval, Keenan, Johnson, & Weber (2014)	3763	0.11 [-0.089, 0.3]	0.28	1
Knobe (2003)	7133	0.61 [0.12, 1.1]	0.015	0.42
Tversky & Gati (1978)	3103	0.1 [-0.047, 0.25]	0.18	1

Supplementary Table 36. **Analysis B2**: Mixed effect model, where participants are clustered by birth country and participants missing birth countries are assumed to be native to the sample country. WEIRDness is operationalizated using CF_{ST}, where countries missing CF_{ST} are excluded from analysis. A *minimum of 100 participants per country* is used as an inclusion criteria. *p* values are adjusted using Bonferroni correction. The values in brackets represent the 2.5% and 97.5% confidence intervals.

Study	*N*	Effect	*p* value	Adjusted *p* value
Huang, Tse, & Cho (2014)	5292	-24 [-63, 14]	0.2	1
Kay, Laurin, Fitzsimons, & Landau (2014)	5365	-0.0042 [-0.27, 0.27]	0.97	1
Alter, Oppenheimer, Epley, & Eyre (2007)	5916	-0.058 [-0.16, 0.043]	0.24	1
Graham, Haidt, & Nosek (2009)	6097	-1.9 [-3.6, -0.26]	0.025	0.71
Rottenstreich & Hsee (2001)	5959	-0.071 [-0.48, 0.33]	0.71	1
Bauer, Wilkie, Kim, & Bodenhausen (2012)	5514	0.064 [-0.16, 0.29]	0.56	1
Miyamoto & Kitayama (2002)	6042	0.73 [-0.6, 2.1]	0.27	1
Inbar, Pizarro, Knobe, & Bloom (2009)	5936	0.23 [-0.97, 1.4]	0.7	1
Critcher & Gilovich (2008)	5408	1.4 [-1.3, 4.2]	0.29	1
Van Lange, Otten, De Bruin, & Joireman (1997)	5065	0.095 [-0.81, 1]	0.83	1
Hauser et al. 1/1 (2007)	5947	-0.38 [-1.2, 0.42]	0.33	1
Anderson, Kraus, Galinsky, & Keltner (2012)	6073	-0.042 [-0.12, 0.036]	0.27	1
Ross, Greene, & House (1977)	5788	-1.5 [-5, 2]	0.37	1
Ross et al. (1977)	5699	-0.015 [-4.6, 4.6]	0.99	1
Giessner & Schubert (2007)	6195	-0.0062 [-0.24, 0.23]	0.96	1
Tversky & Kahneman (1981)	5427	0.051 [-0.34, 0.44]	0.78	1
Hauser et al. 2/2 (2007)	6209	0.33 [-0.059, 0.71]	0.091	1
Risen & Gilovich (2008)	6270	-0.058 [-0.55, 0.43]	0.8	1
Savani, Markus, Naidu, Kumar, & Berlia (2010)	6270	-0.29 [-0.59, 0.011]	0.058	1
Norenzayan, Smith, Kim, & Nisbett (2002)	6279	8.4 [2.8, 14]	0.0063	0.18
Hsee (1998)	6056	0.16 [-0.11, 0.44]	0.23	1
Gray & Wegner (2009)	6270	0.076 [-0.48, 0.63]	0.78	1
Zhong & Liljenquist (2006)	5446	0.0091 [-0.18, 0.2]	0.92	1
Schwarz, Strack, & Mai (1991)	5712	-0.65 [-1.8, 0.53]	0.25	1
Shafir (1993)	6153	-0.036 [-0.43, 0.36]	0.85	1
Zaval, Keenan, Johnson, & Weber (2014)	2883	0.088 [-0.15, 0.32]	0.39	1
Knobe (2003)	6248	0.72 [-0.099, 1.5]	0.08	1
Tversky & Gati (1978)	2184	0.051 [-0.12, 0.22]	0.45	1

Supplementary Table 37. **Analysis B2**: Mixed effect model, where participants are clustered by birth country and participants missing birth countries are assumed to be native to the sample country. WEIRDness is operationalizated using CF_{ST}, where countries missing CF_{ST} are excluded from analysis. A *minimum of 50 participants per country* is used as an inclusion criteria. *p* values are adjusted using Bonferroni correction. The values in brackets represent the 2.5% and 97.5% confidence intervals.

Study	*N*	Effect	*p* value	Adjusted *p* value
Huang, Tse, & Cho (2014)	5763	-28 [-67, 12]	0.16	1
Kay, Laurin, Fitzsimons, & Landau (2014)	5738	-0.045 [-0.27, 0.18]	0.68	1
Alter, Oppenheimer, Epley, & Eyre (2007)	6290	-0.084 [-0.23, 0.059]	0.24	1
Graham, Haidt, & Nosek (2009)	6373	-1.5 [-2.8, -0.11]	0.036	1
Rottenstreich & Hsee (2001)	6333	-0.056 [-0.38, 0.27]	0.72	1
Bauer, Wilkie, Kim, & Bodenhausen (2012)	5888	0.048 [-0.17, 0.27]	0.65	1
Miyamoto & Kitayama (2002)	6315	0.68 [-0.4, 1.8]	0.2	1
Inbar, Pizarro, Knobe, & Bloom (2009)	6310	0.22 [-0.87, 1.3]	0.68	1
Critcher & Gilovich (2008)	5899	1.8 [-0.78, 4.3]	0.16	1
Van Lange, Otten, De Bruin, & Joireman (1997)	5464	0.03 [-0.72, 0.78]	0.94	1
Hauser et al. 1/1 (2007)	6321	-0.37 [-1, 0.27]	0.25	1
Anderson, Kraus, Galinsky, & Keltner (2012)	6349	-0.054 [-0.14, 0.03]	0.2	1
Ross, Greene, & House (1977)	6104	-1.8 [-5, 1.4]	0.25	1
Ross et al. (1977)	6360	0.51 [-3.7, 4.7]	0.8	1
Giessner & Schubert (2007)	6698	0.021 [-0.17, 0.21]	0.82	1
Tversky & Kahneman (1981)	6042	-0.054 [-0.44, 0.33]	0.77	1
Hauser et al. 2/2 (2007)	6721	0.33 [-0.028, 0.7]	0.069	1
Risen & Gilovich (2008)	6784	-0.031 [-0.45, 0.39]	0.88	1
Savani, Markus, Naidu, Kumar, & Berlia (2010)	6785	-0.29 [-0.67, 0.097]	0.14	1
Norenzayan, Smith, Kim, & Nisbett (2002)	6795	6.6 [0.054, 13]	0.048	1
Hsee (1998)	6568	0.17 [-0.1, 0.44]	0.21	1
Gray & Wegner (2009)	6783	0.027 [-0.41, 0.47]	0.9	1
Zhong & Liljenquist (2006)	5983	-0.069 [-0.29, 0.15]	0.52	1
Schwarz, Strack, & Mai (1991)	6374	-0.11 [-1.1, 0.91]	0.83	1
Shafir (1993)	6669	-0.016 [-0.38, 0.35]	0.93	1
Zaval, Keenan, Johnson, & Weber (2014)	3363	0.072 [-0.095, 0.24]	0.37	1
Knobe (2003)	6764	0.59 [-0.15, 1.3]	0.11	1
Tversky & Gati (1978)	2563	0.018 [-0.077, 0.11]	0.68	1

Supplementary Table 38. **Analysis B2**: Mixed effect model, where participants are clustered by birth country and participants missing birth countries are assumed to be native to the sample country. WEIRDness is operationalizated using CF_{ST}, where countries missing CF_{ST} are excluded from analysis. A *minimum of 36 participants per country* is used as an inclusion criteria. *p* values are adjusted using Bonferroni correction. The values in brackets represent the 2.5% and 97.5% confidence intervals.

Study	*N*	Effect	*p* value	Adjusted *p* value
Huang, Tse, & Cho (2014)	5803	-28 [-68, 11]	0.15	1
Kay, Laurin, Fitzsimons, & Landau (2014)	5858	-0.09 [-0.34, 0.16]	0.47	1
Alter, Oppenheimer, Epley, & Eyre (2007)	6411	-0.052 [-0.24, 0.14]	0.58	1
Graham, Haidt, & Nosek (2009)	6495	-1.6 [-2.9, -0.39]	0.013	0.35
Rottenstreich & Hsee (2001)	6455	-0.2 [-0.53, 0.13]	0.22	1
Bauer, Wilkie, Kim, & Bodenhausen (2012)	6009	0.048 [-0.15, 0.24]	0.62	1
Miyamoto & Kitayama (2002)	6437	-0.19 [-1.5, 1.1]	0.77	1
Inbar, Pizarro, Knobe, & Bloom (2009)	6396	0.16 [-1, 1.3]	0.78	1
Critcher & Gilovich (2008)	6032	2.1 [-1.4, 5.5]	0.22	1
Van Lange, Otten, De Bruin, & Joireman (1997)	5630	0.0093 [-0.78, 0.8]	0.98	1
Hauser et al. 1/1 (2007)	6406	-0.34 [-1, 0.35]	0.32	1
Anderson, Kraus, Galinsky, & Keltner (2012)	6471	0.0089 [-0.095, 0.11]	0.86	1
Ross, Greene, & House (1977)	6274	-2.8 [-6.9, 1.3]	0.18	1
Ross et al. (1977)	6360	0.52 [-3.8, 4.8]	0.8	1
Giessner & Schubert (2007)	6784	0.01 [-0.16, 0.18]	0.9	1
Tversky & Kahneman (1981)	6124	-0.14 [-0.5, 0.21]	0.41	1
Hauser et al. 2/2 (2007)	6805	0.26 [-0.13, 0.64]	0.18	1
Risen & Gilovich (2008)	6869	-0.27 [-0.72, 0.19]	0.24	1
Savani, Markus, Naidu, Kumar, & Berlia (2010)	6872	1.5 [-1.5, 4.4]	0.31	1
Norenzayan, Smith, Kim, & Nisbett (2002)	6883	6.8 [0.79, 13]	0.028	0.79
Hsee (1998)	6652	0.17 [-0.078, 0.41]	0.17	1
Gray & Wegner (2009)	6871	0.13 [-0.28, 0.55]	0.51	1
Zhong & Liljenquist (2006)	5983	-0.071 [-0.3, 0.15]	0.52	1
Schwarz, Strack, & Mai (1991)	6457	-0.035 [-1, 0.95]	0.94	1
Shafir (1993)	6754	0.14 [-0.29, 0.57]	0.5	1
Zaval, Keenan, Johnson, & Weber (2014)	3540	0.074 [-0.066, 0.21]	0.28	1
Knobe (2003)	6853	0.97 [0.19, 1.7]	0.017	0.46
Tversky & Gati (1978)	2940	0.025 [-0.23, 0.28]	0.84	1

Supplementary Table 39. **Analysis B2**: Mixed effect model, where participants are clustered by birth country and participants missing birth countries are assumed to be native to the sample country. WEIRDness is operationalizated using CF_{ST}, where countries missing CF_{ST} are excluded from analysis. A *minimum of 10 participants per country* is used as an inclusion criteria. *p* values are adjusted using Bonferroni correction. The values in brackets represent the 2.5% and 97.5% confidence intervals.

Study	*N*	Effect	*p* value	Adjusted *p* value
Huang, Tse, & Cho (2014)	5963	10 [-19, 40]	0.49	1
Kay, Laurin, Fitzsimons, & Landau (2014)	5958	-0.14 [-0.41, 0.12]	0.28	1
Alter, Oppenheimer, Epley, & Eyre (2007)	6510	-0.021 [-0.23, 0.19]	0.84	1
Graham, Haidt, & Nosek (2009)	6595	-0.83 [-1.7, 0.029]	0.058	1
Rottenstreich & Hsee (2001)	6555	0.15 [-0.12, 0.42]	0.26	1
Bauer, Wilkie, Kim, & Bodenhausen (2012)	6109	0.26 [0.0068, 0.52]	0.045	1
Miyamoto & Kitayama (2002)	6537	-0.75 [-2.1, 0.61]	0.27	1
Inbar, Pizarro, Knobe, & Bloom (2009)	6489	0.072 [-0.74, 0.88]	0.86	1
Critcher & Gilovich (2008)	6143	-2.7 [-6.6, 1.2]	0.17	1
Van Lange, Otten, De Bruin, & Joireman (1997)	5715	0.011 [-0.5, 0.53]	0.97	1
Hauser et al. 1/1 (2007)	6541	3.2 [0.82, 5.6]	0.01	0.29
Anderson, Kraus, Galinsky, & Keltner (2012)	6571	-0.1 [-0.2, -0.0061]	0.038	1
Ross, Greene, & House (1977)	6361	-2.7 [-5.6, 0.23]	0.069	1
Ross et al. (1977)	6687	1.2 [-2.8, 5.2]	0.54	1
Giessner & Schubert (2007)	7087	-0.21 [-0.45, 0.03]	0.085	1
Tversky & Kahneman (1981)	6435	-1.1 [-3.7, 1.5]	0.39	1
Hauser et al. 2/2 (2007)	7115	1.1 [-0.88, 3]	0.27	1
Risen & Gilovich (2008)	7184	-0.12 [-0.63, 0.39]	0.64	1
Savani, Markus, Naidu, Kumar, & Berlia (2010)	7186	1.5 [-2.4, 5.4]	0.45	1
Norenzayan, Smith, Kim, & Nisbett (2002)	7198	4.1 [-0.97, 9.2]	0.11	1
Hsee (1998)	6984	0.19 [-0.059, 0.45]	0.13	1
Gray & Wegner (2009)	7185	0.16 [-0.21, 0.53]	0.39	1
Zhong & Liljenquist (2006)	6321	0.053 [-0.22, 0.32]	0.69	1
Schwarz, Strack, & Mai (1991)	6710	-0.1 [-0.66, 0.46]	0.72	1
Shafir (1993)	7082	0.33 [-1.4, 2]	0.7	1
Zaval, Keenan, Johnson, & Weber (2014)	3759	0.095 [-0.098, 0.29]	0.32	1
Knobe (2003)	7166	0.63 [0.11, 1.1]	0.02	0.55
Tversky & Gati (1978)	3113	0.09 [-0.061, 0.24]	0.23	1

Supplementary Table 40. **Analysis B2**: Mixed effect model, where participants are clustered by birth country and participants missing birth countries are assumed to be native to the sample country. WEIRDness is operationalizated using CF_{ST}, imputing the CF_{ST} for countries missing cultural distance. A *minimum of 100 participants per country* is used as an inclusion criteria. *p* values are adjusted using Bonferroni correction. The values in brackets represent the 2.5% and 97.5% confidence intervals.

Study	*N*	Effect	*p* value	Adjusted *p* value
Huang, Tse, & Cho (2014)	5393	-26 [-64, 12]	0.17	1
Kay, Laurin, Fitzsimons, & Landau (2014)	5467	-0.0062 [-0.24, 0.23]	0.96	1
Alter, Oppenheimer, Epley, & Eyre (2007)	6123	-0.015 [-0.12, 0.094]	0.77	1
Graham, Haidt, & Nosek (2009)	6306	-1.4 [-2.9, 0.096]	0.065	1
Rottenstreich & Hsee (2001)	6167	-0.16 [-0.54, 0.21]	0.38	1
Bauer, Wilkie, Kim, & Bodenhausen (2012)	5618	-0.036 [-0.27, 0.2]	0.75	1
Miyamoto & Kitayama (2002)	6249	0.7 [-0.55, 1.9]	0.26	1
Inbar, Pizarro, Knobe, & Bloom (2009)	6144	0.17 [-0.88, 1.2]	0.73	1
Critcher & Gilovich (2008)	5508	1.8 [-1.4, 4.9]	0.25	1
Van Lange, Otten, De Bruin, & Joireman (1997)	5065	0.095 [-0.81, 1]	0.83	1
Hauser et al. 1/1 (2007)	6151	-0.33 [-1, 0.36]	0.33	1
Anderson, Kraus, Galinsky, & Keltner (2012)	6281	-0.013 [-0.088, 0.062]	0.72	1
Ross, Greene, & House (1977)	5890	-1.8 [-5.4, 1.8]	0.31	1
Ross et al. (1977)	5868	1.9 [-3.1, 6.8]	0.43	1
Giessner & Schubert (2007)	6381	-0.016 [-0.22, 0.19]	0.87	1
Tversky & Kahneman (1981)	5619	0.049 [-0.3, 0.39]	0.76	1
Hauser et al. 2/2 (2007)	6400	0.24 [-0.12, 0.61]	0.18	1
Risen & Gilovich (2008)	6465	-0.12 [-0.57, 0.33]	0.58	1
Savani, Markus, Naidu, Kumar, & Berlia (2010)	6464	-0.28 [-0.55, -0.0091]	0.044	1
Norenzayan, Smith, Kim, & Nisbett (2002)	6475	6.7 [1.2, 12]	0.021	0.58
Hsee (1998)	6250	0.065 [-0.22, 0.35]	0.63	1
Gray & Wegner (2009)	6465	0.18 [-0.34, 0.7]	0.47	1
Zhong & Liljenquist (2006)	5596	-0.044 [-0.23, 0.14]	0.61	1
Schwarz, Strack, & Mai (1991)	5897	-0.69 [-1.7, 0.33]	0.17	1
Shafir (1993)	6349	-0.17 [-0.58, 0.24]	0.38	1
Zaval, Keenan, Johnson, & Weber (2014)	2883	0.088 [-0.14, 0.32]	0.39	1
Knobe (2003)	6442	0.74 [0.0019, 1.5]	0.049	1
Tversky & Gati (1978)	2184	0.051 [-0.12, 0.22]	0.45	1

Supplementary Table 41. **Analysis B2**: Mixed effect model, where participants are clustered by birth country and participants missing birth countries are assumed to be native to the sample country. WEIRDness is operationalizated using CF_{ST}, imputing the CF_{ST} for countries missing cultural distance. A *minimum of 50 participants per country* is used as an inclusion criteria. *p* values are adjusted using Bonferroni correction. The values in brackets represent the 2.5% and 97.5% confidence intervals.

Study	*N*	Effect	*p* value	Adjusted *p* value
Huang, Tse, & Cho (2014)	6029	-25 [-60, 10]	0.16	1
Kay, Laurin, Fitzsimons, & Landau (2014)	5913	-0.045 [-0.25, 0.16]	0.65	1
Alter, Oppenheimer, Epley, & Eyre (2007)	6570	-0.047 [-0.18, 0.088]	0.48	1
Graham, Haidt, & Nosek (2009)	6657	-1.1 [-2.3, 0.17]	0.087	1
Rottenstreich & Hsee (2001)	6616	-0.13 [-0.44, 0.19]	0.41	1
Bauer, Wilkie, Kim, & Bodenhausen (2012)	6067	-0.028 [-0.25, 0.2]	0.8	1
Miyamoto & Kitayama (2002)	6597	0.67 [-0.37, 1.7]	0.2	1
Inbar, Pizarro, Knobe, & Bloom (2009)	6593	0.14 [-0.84, 1.1]	0.77	1
Critcher & Gilovich (2008)	6163	0.97 [-2.3, 4.3]	0.55	1
Van Lange, Otten, De Bruin, & Joireman (1997)	5631	0.087 [-0.64, 0.81]	0.81	1
Hauser et al. 1/1 (2007)	6599	-0.31 [-0.88, 0.26]	0.27	1
Anderson, Kraus, Galinsky, & Keltner (2012)	6632	-0.033 [-0.12, 0.055]	0.44	1
Ross, Greene, & House (1977)	6373	-2.5 [-5.5, 0.44]	0.091	1
Ross et al. (1977)	6763	2.1 [-2.2, 6.3]	0.32	1
Giessner & Schubert (2007)	7128	0.017 [-0.18, 0.22]	0.86	1
Tversky & Kahneman (1981)	6476	-0.058 [-0.38, 0.27]	0.72	1
Hauser et al. 2/2 (2007)	7155	0.25 [-0.075, 0.58]	0.12	1
Risen & Gilovich (2008)	7226	-0.058 [-0.44, 0.32]	0.76	1
Savani, Markus, Naidu, Kumar, & Berlia (2010)	7226	-0.3 [-0.72, 0.11]	0.14	1
Norenzayan, Smith, Kim, & Nisbett (2002)	7237	5.7 [-0.45, 12]	0.068	1
Hsee (1998)	6913	0.13 [-0.17, 0.43]	0.38	1
Gray & Wegner (2009)	7224	0.079 [-0.34, 0.5]	0.7	1
Zhong & Liljenquist (2006)	6374	-0.14 [-0.36, 0.083]	0.21	1
Schwarz, Strack, & Mai (1991)	6715	-0.16 [-1.1, 0.75]	0.71	1
Shafir (1993)	7109	-0.11 [-0.44, 0.23]	0.52	1
Zaval, Keenan, Johnson, & Weber (2014)	3547	0.095 [-0.047, 0.24]	0.18	1
Knobe (2003)	7205	0.57 [-0.07, 1.2]	0.078	1
Tversky & Gati (1978)	2650	0.05 [-0.045, 0.14]	0.27	1

Supplementary Table 42. **Analysis B2**: Mixed effect model, where participants are clustered by birth country and participants missing birth countries are assumed to be native to the sample country. WEIRDness is operationalizated using CF_{ST}, imputing the CF_{ST} for countries missing cultural distance. A *minimum of 36 participants per country* is used as an inclusion criteria. *p* values are adjusted using Bonferroni correction. The values in brackets represent the 2.5% and 97.5% confidence intervals.

Study	*N*	Effect	*p* value	Adjusted *p* value
Huang, Tse, & Cho (2014)	6111	-24 [-58, 11]	0.17	1
Kay, Laurin, Fitzsimons, & Landau (2014)	6076	-0.06 [-0.29, 0.17]	0.6	1
Alter, Oppenheimer, Epley, & Eyre (2007)	6735	-0.045 [-0.23, 0.14]	0.63	1
Graham, Haidt, & Nosek (2009)	6859	-1.3 [-2.5, -0.17]	0.026	0.73
Rottenstreich & Hsee (2001)	6817	-0.27 [-0.58, 0.047]	0.093	1
Bauer, Wilkie, Kim, & Bodenhausen (2012)	6267	-0.022 [-0.23, 0.19]	0.83	1
Miyamoto & Kitayama (2002)	6762	0.059 [-1.2, 1.3]	0.93	1
Inbar, Pizarro, Knobe, & Bloom (2009)	6679	0.072 [-0.97, 1.1]	0.89	1
Critcher & Gilovich (2008)	6337	1.6 [-2.3, 5.5]	0.4	1
Van Lange, Otten, De Bruin, & Joireman (1997)	5839	0.092 [-0.65, 0.83]	0.8	1
Hauser et al. 1/1 (2007)	6764	-0.23 [-0.84, 0.39]	0.46	1
Anderson, Kraus, Galinsky, & Keltner (2012)	6834	0.01 [-0.098, 0.12]	0.85	1
Ross, Greene, & House (1977)	6583	-3.3 [-6.9, 0.42]	0.08	1
Ross et al. (1977)	6763	2.1 [-2.2, 6.5]	0.32	1
Giessner & Schubert (2007)	7214	0.011 [-0.17, 0.2]	0.9	1
Tversky & Kahneman (1981)	6558	-0.13 [-0.44, 0.18]	0.39	1
Hauser et al. 2/2 (2007)	7239	0.2 [-0.14, 0.55]	0.24	1
Risen & Gilovich (2008)	7311	-0.26 [-0.68, 0.16]	0.21	1
Savani, Markus, Naidu, Kumar, & Berlia (2010)	7313	1.2 [-1.4, 3.7]	0.37	1
Norenzayan, Smith, Kim, & Nisbett (2002)	7325	6.2 [0.33, 12]	0.039	1
Hsee (1998)	6997	0.14 [-0.14, 0.42]	0.32	1
Gray & Wegner (2009)	7312	0.16 [-0.24, 0.57]	0.42	1
Zhong & Liljenquist (2006)	6374	-0.14 [-0.37, 0.085]	0.21	1
Schwarz, Strack, & Mai (1991)	6843	-0.042 [-0.93, 0.85]	0.92	1
Shafir (1993)	7194	0.033 [-0.36, 0.43]	0.86	1
Zaval, Keenan, Johnson, & Weber (2014)	3724	0.092 [-0.033, 0.22]	0.14	1
Knobe (2003)	7294	0.9 [0.2, 1.6]	0.014	0.38
Tversky & Gati (1978)	3103	0.042 [-0.17, 0.26]	0.69	1

Supplementary Table 43. **Analysis B2**: Mixed effect model, where participants are clustered by birth country and participants missing birth countries are assumed to be native to the sample country. WEIRDness is operationalizated using CF_{ST}, imputing the CF_{ST} for countries missing cultural distance. A *minimum of 10 participants per country* is used as an inclusion criteria. *p* values are adjusted using Bonferroni correction. The values in brackets represent the 2.5% and 97.5% confidence intervals.

Study	*N*	Effect	*p* value	Adjusted *p* value
Huang, Tse, & Cho (2014)	6304	7.9 [-19, 35]	0.55	1
Kay, Laurin, Fitzsimons, & Landau (2014)	6211	-0.12 [-0.37, 0.13]	0.33	1
Alter, Oppenheimer, Epley, & Eyre (2007)	6869	-0.018 [-0.22, 0.18]	0.86	1
Graham, Haidt, & Nosek (2009)	6959	-0.71 [-1.5, 0.1]	0.084	1
Rottenstreich & Hsee (2001)	6917	0.086 [-0.18, 0.35]	0.52	1
Bauer, Wilkie, Kim, & Bodenhausen (2012)	6367	0.21 [-0.04, 0.46]	0.097	1
Miyamoto & Kitayama (2002)	6897	-0.63 [-1.9, 0.67]	0.33	1
Inbar, Pizarro, Knobe, & Bloom (2009)	6807	0.04 [-0.71, 0.79]	0.91	1
Critcher & Gilovich (2008)	6482	-2.4 [-6.3, 1.5]	0.21	1
Van Lange, Otten, De Bruin, & Joireman (1997)	5957	0.044 [-0.45, 0.54]	0.86	1
Hauser et al. 1/1 (2007)	6899	3 [0.76, 5.2]	0.0098	0.27
Anderson, Kraus, Galinsky, & Keltner (2012)	6934	-0.092 [-0.19, 0.0046]	0.061	1
Ross, Greene, & House (1977)	6704	-2.9 [-5.6, -0.28]	0.031	0.87
Ross et al. (1977)	7090	1.5 [-2.3, 5.4]	0.42	1
Giessner & Schubert (2007)	7517	-0.2 [-0.43, 0.037]	0.097	1
Tversky & Kahneman (1981)	6869	-1 [-3.4, 1.4]	0.41	1
Hauser et al. 2/2 (2007)	7549	1.1 [-0.79, 2.9]	0.25	1
Risen & Gilovich (2008)	7626	-0.15 [-0.63, 0.34]	0.54	1
Savani, Markus, Naidu, Kumar, & Berlia (2010)	7627	1.4 [-2.3, 5]	0.46	1
Norenzayan, Smith, Kim, & Nisbett (2002)	7640	4.1 [-0.87, 9]	0.1	1
Hsee (1998)	7329	0.17 [-0.084, 0.43]	0.18	1
Gray & Wegner (2009)	7626	0.18 [-0.18, 0.54]	0.32	1
Zhong & Liljenquist (2006)	6712	0.018 [-0.24, 0.28]	0.89	1
Schwarz, Strack, & Mai (1991)	7096	-0.091 [-0.63, 0.45]	0.73	1
Shafir (1993)	7522	0.3 [-1.3, 1.9]	0.71	1
Zaval, Keenan, Johnson, & Weber (2014)	3968	0.086 [-0.094, 0.27]	0.34	1
Knobe (2003)	7607	0.62 [0.13, 1.1]	0.015	0.42
Tversky & Gati (1978)	3307	0.092 [-0.048, 0.23]	0.19	1

Supplementary Table 44. **Analysis B2**: Mixed effect model, where participants are clustered by their country of origin, where country of origin is based on participants stated hometowns. WEIRDness is operationalizated using CF_{ST}, where countries missing CF_{ST} are excluded from analysis. A *minimum of 100 participants per country* is used as an inclusion criteria. *p* values are adjusted using Bonferroni correction. The values in brackets represent the 2.5% and 97.5% confidence intervals.

Study	*N*	Effect	*p* value	Adjusted *p* value
Huang, Tse, & Cho (2014)	5279	-24 [-63, 15]	0.2	1
Kay, Laurin, Fitzsimons, & Landau (2014)	5351	-0.0092 [-0.28, 0.26]	0.94	1
Alter, Oppenheimer, Epley, & Eyre (2007)	5899	-0.057 [-0.16, 0.044]	0.25	1
Graham, Haidt, & Nosek (2009)	6054	-2 [-3.6, -0.35]	0.02	0.57
Rottenstreich & Hsee (2001)	5940	-0.055 [-0.45, 0.34]	0.77	1
Bauer, Wilkie, Kim, & Bodenhausen (2012)	5484	0.069 [-0.15, 0.29]	0.52	1
Miyamoto & Kitayama (2002)	6026	0.66 [-0.7, 2]	0.32	1
Inbar, Pizarro, Knobe, & Bloom (2009)	5919	0.21 [-0.96, 1.4]	0.71	1
Critcher & Gilovich (2008)	5396	1.6 [-1.3, 4.6]	0.26	1
Van Lange, Otten, De Bruin, & Joireman (1997)	5056	0.033 [-0.86, 0.93]	0.94	1
Hauser et al. 1/1 (2007)	5931	-0.39 [-1.2, 0.41]	0.32	1
Anderson, Kraus, Galinsky, & Keltner (2012)	6053	-0.037 [-0.12, 0.041]	0.33	1
Ross, Greene, & House (1977)	5773	-1.6 [-5.3, 2.1]	0.37	1
Ross et al. (1977)	5676	0.29 [-4.2, 4.8]	0.89	1
Giessner & Schubert (2007)	6163	-0.021 [-0.25, 0.21]	0.85	1
Tversky & Kahneman (1981)	5404	0.045 [-0.34, 0.43]	0.8	1
Hauser et al. 2/2 (2007)	6181	0.3 [-0.071, 0.68]	0.1	1
Risen & Gilovich (2008)	6236	-0.028 [-0.54, 0.48]	0.91	1
Savani, Markus, Naidu, Kumar, & Berlia (2010)	6240	-0.3 [-0.6, 0.0047]	0.053	1
Norenzayan, Smith, Kim, & Nisbett (2002)	6244	8.2 [2.5, 14]	0.0079	0.22
Hsee (1998)	6022	0.16 [-0.13, 0.44]	0.26	1
Gray & Wegner (2009)	6236	0.057 [-0.5, 0.62]	0.83	1
Zhong & Liljenquist (2006)	5426	-0.0091 [-0.2, 0.18]	0.92	1
Schwarz, Strack, & Mai (1991)	5684	-0.69 [-1.8, 0.46]	0.22	1
Shafir (1993)	6118	-0.037 [-0.43, 0.36]	0.84	1
Zaval, Keenan, Johnson, & Weber (2014)	2870	0.083 [-0.15, 0.32]	0.41	1
Knobe (2003)	6217	0.71 [-0.098, 1.5]	0.08	1
Tversky & Gati (1978)	2175	0.064 [-0.15, 0.27]	0.45	1

Supplementary Table 45. **Analysis B2**: Mixed effect model, where participants are clustered by their country of origin, where country of origin is based on participants stated hometowns. WEIRDness is operationalizated using CF_{ST}, where countries missing CF_{ST} are excluded from analysis. A *minimum of 50 participants per country* is used as an inclusion criteria. *p* values are adjusted using Bonferroni correction. The values in brackets represent the 2.5% and 97.5% confidence intervals.

Study	*N*	Effect	*p* value	Adjusted *p* value
Huang, Tse, & Cho (2014)	5748	-28 [-67, 12]	0.16	1
Kay, Laurin, Fitzsimons, & Landau (2014)	5723	-0.05 [-0.28, 0.18]	0.66	1
Alter, Oppenheimer, Epley, & Eyre (2007)	6272	-0.084 [-0.23, 0.061]	0.24	1
Graham, Haidt, & Nosek (2009)	6329	-1.5 [-2.9, -0.18]	0.028	0.8
Rottenstreich & Hsee (2001)	6313	-0.044 [-0.36, 0.27]	0.78	1
Bauer, Wilkie, Kim, & Bodenhausen (2012)	5857	0.052 [-0.17, 0.27]	0.62	1
Miyamoto & Kitayama (2002)	6298	0.63 [-0.47, 1.7]	0.25	1
Inbar, Pizarro, Knobe, & Bloom (2009)	6292	0.21 [-0.87, 1.3]	0.69	1
Critcher & Gilovich (2008)	5885	2 [-0.64, 4.6]	0.13	1
Van Lange, Otten, De Bruin, & Joireman (1997)	5454	-0.011 [-0.75, 0.73]	0.98	1
Hauser et al. 1/1 (2007)	6304	-0.37 [-1, 0.26]	0.24	1
Anderson, Kraus, Galinsky, & Keltner (2012)	6328	-0.05 [-0.14, 0.036]	0.24	1
Ross, Greene, & House (1977)	6088	-1.9 [-5.2, 1.5]	0.25	1
Ross et al. (1977)	6336	0.81 [-3.5, 5.1]	0.7	1
Giessner & Schubert (2007)	6664	0.0057 [-0.18, 0.19]	0.95	1
Tversky & Kahneman (1981)	6016	-0.056 [-0.44, 0.33]	0.77	1
Hauser et al. 2/2 (2007)	6691	0.32 [-0.039, 0.67]	0.078	1
Risen & Gilovich (2008)	6748	-0.0055 [-0.46, 0.45]	0.98	1
Savani, Markus, Naidu, Kumar, & Berlia (2010)	6753	-0.3 [-0.7, 0.11]	0.14	1
Norenzayan, Smith, Kim, & Nisbett (2002)	6758	6.5 [-0.011, 13]	0.05	1
Hsee (1998)	6532	0.17 [-0.11, 0.44]	0.21	1
Gray & Wegner (2009)	6747	0.018 [-0.42, 0.45]	0.93	1
Zhong & Liljenquist (2006)	5962	-0.08 [-0.3, 0.14]	0.44	1
Schwarz, Strack, & Mai (1991)	6342	-0.13 [-1.1, 0.86]	0.78	1
Shafir (1993)	6632	-0.017 [-0.37, 0.34]	0.92	1
Zaval, Keenan, Johnson, & Weber (2014)	3350	0.073 [-0.09, 0.24]	0.35	1
Knobe (2003)	6731	0.59 [-0.14, 1.3]	0.11	1
Tversky & Gati (1978)	2554	0.018 [-0.073, 0.11]	0.66	1

Supplementary Table 46. **Analysis B2**: Mixed effect model, where participants are clustered by their country of origin, where country of origin is based on participants stated hometowns. WEIRDness is operationalizated using CF_{ST}, where countries missing CF_{ST} are excluded from analysis. A *minimum of 36 participants per country* is used as an inclusion criteria. *p* values are adjusted using Bonferroni correction. The values in brackets represent the 2.5% and 97.5% confidence intervals.

Study	*N*	Effect	*p* value	Adjusted *p* value
Huang, Tse, & Cho (2014)	5788	-28 [-67, 11]	0.15	1
Kay, Laurin, Fitzsimons, & Landau (2014)	5807	-0.08 [-0.37, 0.21]	0.57	1
Alter, Oppenheimer, Epley, & Eyre (2007)	6357	-0.066 [-0.28, 0.15]	0.54	1
Graham, Haidt, & Nosek (2009)	6415	-1.6 [-3, -0.19]	0.028	0.78
Rottenstreich & Hsee (2001)	6399	-0.049 [-0.39, 0.29]	0.77	1
Bauer, Wilkie, Kim, & Bodenhausen (2012)	5942	0.043 [-0.18, 0.26]	0.69	1
Miyamoto & Kitayama (2002)	6384	0.66 [-0.53, 1.9]	0.26	1
Inbar, Pizarro, Knobe, & Bloom (2009)	6378	0.15 [-0.99, 1.3]	0.79	1
Critcher & Gilovich (2008)	6018	2.3 [-1.2, 5.8]	0.19	1
Van Lange, Otten, De Bruin, & Joireman (1997)	5584	0.031 [-0.86, 0.93]	0.94	1
Hauser et al. 1/1 (2007)	6389	-0.35 [-1, 0.34]	0.31	1
Anderson, Kraus, Galinsky, & Keltner (2012)	6414	-0.04 [-0.15, 0.066]	0.44	1
Ross, Greene, & House (1977)	6222	-2.4 [-7.1, 2.3]	0.3	1
Ross et al. (1977)	6336	0.83 [-3.6, 5.2]	0.7	1
Giessner & Schubert (2007)	6741	0.036 [-0.14, 0.21]	0.67	1
Tversky & Kahneman (1981)	6091	-0.14 [-0.5, 0.22]	0.43	1
Hauser et al. 2/2 (2007)	6767	0.13 [-0.37, 0.62]	0.6	1
Risen & Gilovich (2008)	6824	-0.15 [-0.59, 0.28]	0.47	1
Savani, Markus, Naidu, Kumar, & Berlia (2010)	6831	1.4 [-1.5, 4.4]	0.32	1
Norenzayan, Smith, Kim, & Nisbett (2002)	6837	6.8 [0.78, 13]	0.028	0.8
Hsee (1998)	6608	0.2 [-0.044, 0.45]	0.1	1
Gray & Wegner (2009)	6826	0.12 [-0.29, 0.53]	0.56	1
Zhong & Liljenquist (2006)	5962	-0.082 [-0.3, 0.14]	0.44	1
Schwarz, Strack, & Mai (1991)	6417	-0.12 [-1.1, 0.82]	0.79	1
Shafir (1993)	6706	0.13 [-0.29, 0.55]	0.53	1
Zaval, Keenan, Johnson, & Weber (2014)	3524	0.07 [-0.064, 0.2]	0.29	1
Knobe (2003)	6810	1 [0.22, 1.8]	0.015	0.43
Tversky & Gati (1978)	2930	0.024 [-0.22, 0.27]	0.84	1

Supplementary Table 47. **Analysis B2**: Mixed effect model, where participants are clustered by their country of origin, where country of origin is based on participants stated hometowns. WEIRDness is operationalizated using CF_{ST}, where countries missing CF_{ST} are excluded from analysis. A *minimum of 10 participants per country* is used as an inclusion criteria. *p* values are adjusted using Bonferroni correction. The values in brackets represent the 2.5% and 97.5% confidence intervals.

Study	*N*	Effect	*p* value	Adjusted *p* value
Huang, Tse, & Cho (2014)	5947	10 [-19, 40]	0.49	1
Kay, Laurin, Fitzsimons, & Landau (2014)	5941	-0.14 [-0.4, 0.12]	0.27	1
Alter, Oppenheimer, Epley, & Eyre (2007)	6490	-0.015 [-0.23, 0.2]	0.88	1
Graham, Haidt, & Nosek (2009)	6549	-0.92 [-1.8, -0.05]	0.039	1
Rottenstreich & Hsee (2001)	6533	0.16 [-0.1, 0.43]	0.22	1
Bauer, Wilkie, Kim, & Bodenhausen (2012)	6076	0.26 [-5.7e-05, 0.53]	0.05	1
Miyamoto & Kitayama (2002)	6518	-0.77 [-2.1, 0.59]	0.26	1
Inbar, Pizarro, Knobe, & Bloom (2009)	6458	0.052 [-0.76, 0.86]	0.9	1
Critcher & Gilovich (2008)	6127	-2.4 [-6.1, 1.3]	0.2	1
Van Lange, Otten, De Bruin, & Joireman (1997)	5703	-0.013 [-0.52, 0.5]	0.96	1
Hauser et al. 1/1 (2007)	6522	3.2 [0.75, 5.7]	0.012	0.34
Anderson, Kraus, Galinsky, & Keltner (2012)	6548	-0.1 [-0.2, -0.0042]	0.042	1
Ross, Greene, & House (1977)	6343	-2.6 [-5.9, 0.82]	0.13	1
Ross et al. (1977)	6655	0.89 [-3.2, 4.9]	0.66	1
Giessner & Schubert (2007)	7038	-0.17 [-0.41, 0.058]	0.14	1
Tversky & Kahneman (1981)	6386	-1.1 [-3.7, 1.4]	0.38	1
Hauser et al. 2/2 (2007)	7061	0.99 [-1, 3]	0.32	1
Risen & Gilovich (2008)	7123	-0.12 [-0.61, 0.36]	0.61	1
Savani, Markus, Naidu, Kumar, & Berlia (2010)	7129	1.5 [-2.5, 5.4]	0.46	1
Norenzayan, Smith, Kim, & Nisbett (2002)	7136	4.8 [0.1, 9.5]	0.045	1
Hsee (1998)	6924	0.19 [-0.072, 0.44]	0.15	1
Gray & Wegner (2009)	7124	0.16 [-0.23, 0.55]	0.41	1
Zhong & Liljenquist (2006)	6287	0.06 [-0.21, 0.33]	0.65	1
Schwarz, Strack, & Mai (1991)	6664	-0.14 [-0.68, 0.41]	0.61	1
Shafir (1993)	7028	0.31 [-1.4, 2]	0.71	1
Zaval, Keenan, Johnson, & Weber (2014)	3740	0.089 [-0.11, 0.29]	0.36	1
Knobe (2003)	7107	0.63 [0.092, 1.2]	0.023	0.64
Tversky & Gati (1978)	3095	0.078 [-0.073, 0.23]	0.3	1

Supplementary Table 48. **Analysis B2**: Mixed effect model, where participants are clustered by their country of origin, where country of origin is based on participants stated hometowns. WEIRDness is operationalizated using CF_{ST}, imputing the CF_{ST} for countries missing cultural distance. A *minimum of 100 participants per country* is used as an inclusion criteria. *p* values are adjusted using Bonferroni correction. The values in brackets represent the 2.5% and 97.5% confidence intervals.

Study	*N*	Effect	*p* value	Adjusted *p* value
Huang, Tse, & Cho (2014)	5380	-26 [-64, 12]	0.17	1
Kay, Laurin, Fitzsimons, & Landau (2014)	5451	-0.012 [-0.25, 0.23]	0.92	1
Alter, Oppenheimer, Epley, & Eyre (2007)	6104	-0.015 [-0.12, 0.094]	0.78	1
Graham, Haidt, & Nosek (2009)	6261	-1.5 [-3, 0.024]	0.053	1
Rottenstreich & Hsee (2001)	6146	-0.16 [-0.53, 0.21]	0.38	1
Bauer, Wilkie, Kim, & Bodenhausen (2012)	5586	-0.039 [-0.27, 0.2]	0.73	1
Miyamoto & Kitayama (2002)	6231	0.64 [-0.62, 1.9]	0.3	1
Inbar, Pizarro, Knobe, & Bloom (2009)	6125	0.18 [-0.85, 1.2]	0.72	1
Critcher & Gilovich (2008)	5496	2 [-1.3, 5.3]	0.22	1
Van Lange, Otten, De Bruin, & Joireman (1997)	5056	0.033 [-0.86, 0.92]	0.94	1
Hauser et al. 1/1 (2007)	6134	-0.34 [-1, 0.34]	0.31	1
Anderson, Kraus, Galinsky, & Keltner (2012)	6259	-0.0088 [-0.084, 0.066]	0.81	1
Ross, Greene, & House (1977)	5875	-1.9 [-5.6, 1.9]	0.31	1
Ross et al. (1977)	5844	1.8 [-2.8, 6.5]	0.41	1
Giessner & Schubert (2007)	6349	-0.033 [-0.24, 0.18]	0.74	1
Tversky & Kahneman (1981)	5595	0.043 [-0.3, 0.38]	0.79	1
Hauser et al. 2/2 (2007)	6370	0.22 [-0.14, 0.57]	0.22	1
Risen & Gilovich (2008)	6429	-0.093 [-0.56, 0.37]	0.67	1
Savani, Markus, Naidu, Kumar, & Berlia (2010)	6433	-0.29 [-0.56, -0.022]	0.036	1
Norenzayan, Smith, Kim, & Nisbett (2002)	6437	6.5 [0.86, 12]	0.026	0.74
Hsee (1998)	6214	0.061 [-0.23, 0.36]	0.66	1
Gray & Wegner (2009)	6428	0.16 [-0.36, 0.68]	0.52	1
Zhong & Liljenquist (2006)	5575	-0.07 [-0.26, 0.12]	0.43	1
Schwarz, Strack, & Mai (1991)	5866	-0.68 [-1.7, 0.33]	0.17	1
Shafir (1993)	6311	-0.16 [-0.57, 0.24]	0.4	1
Zaval, Keenan, Johnson, & Weber (2014)	2870	0.083 [-0.15, 0.31]	0.41	1
Knobe (2003)	6409	0.73 [0.0022, 1.5]	0.049	1
Tversky & Gati (1978)	2175	0.063 [-0.15, 0.27]	0.45	1

Supplementary Table 49. **Analysis B2**: Mixed effect model, where participants are clustered by their country of origin, where country of origin is based on participants stated hometowns. WEIRDness is operationalizated using CF_{ST}, imputing the CF_{ST} for countries missing cultural distance. A *minimum of 50 participants per country is used as an inclusion criteria*. *p* values are adjusted using Bonferroni correction. The values in brackets represent the 2.5% and 97.5% confidence intervals.

Study	*N*	Effect	*p* value	Adjusted *p* value
Huang, Tse, & Cho (2014)	6009	-25 [-60, 9.7]	0.15	1
Kay, Laurin, Fitzsimons, & Landau (2014)	5893	-0.047 [-0.25, 0.16]	0.64	1
Alter, Oppenheimer, Epley, & Eyre (2007)	6547	-0.046 [-0.18, 0.089]	0.49	1
Graham, Haidt, & Nosek (2009)	6608	-1.1 [-2.4, 0.1]	0.071	1
Rottenstreich & Hsee (2001)	6591	-0.12 [-0.43, 0.19]	0.42	1
Bauer, Wilkie, Kim, & Bodenhausen (2012)	6031	-0.034 [-0.25, 0.19]	0.75	1
Miyamoto & Kitayama (2002)	6575	0.62 [-0.44, 1.7]	0.24	1
Inbar, Pizarro, Knobe, & Bloom (2009)	6570	0.15 [-0.81, 1.1]	0.76	1
Critcher & Gilovich (2008)	6145	1.3 [-2, 4.5]	0.42	1
Van Lange, Otten, De Bruin, & Joireman (1997)	5618	0.045 [-0.69, 0.78]	0.9	1
Hauser et al. 1/1 (2007)	6578	-0.32 [-0.88, 0.24]	0.25	1
Anderson, Kraus, Galinsky, & Keltner (2012)	6606	-0.032 [-0.13, 0.063]	0.5	1
Ross, Greene, & House (1977)	6353	-2.7 [-5.8, 0.41]	0.086	1
Ross et al. (1977)	6739	2 [-2.2, 6.2]	0.33	1
Giessner & Schubert (2007)	7094	4.7e-05 [-0.2, 0.2]	1	1
Tversky & Kahneman (1981)	6450	-0.061 [-0.39, 0.27]	0.71	1
Hauser et al. 2/2 (2007)	7124	0.23 [-0.096, 0.55]	0.16	1
Risen & Gilovich (2008)	7189	-0.028 [-0.44, 0.38]	0.89	1
Savani, Markus, Naidu, Kumar, & Berlia (2010)	7194	-0.32 [-0.75, 0.11]	0.13	1
Norenzayan, Smith, Kim, & Nisbett (2002)	7198	5.5 [-0.61, 12]	0.075	1
Hsee (1998)	6877	0.13 [-0.17, 0.43]	0.38	1
Gray & Wegner (2009)	7186	0.072 [-0.35, 0.49]	0.73	1
Zhong & Liljenquist (2006)	6353	-0.15 [-0.37, 0.062]	0.15	1
Schwarz, Strack, & Mai (1991)	6681	-0.15 [-1, 0.74]	0.73	1
Shafir (1993)	7070	-0.1 [-0.43, 0.22]	0.52	1
Zaval, Keenan, Johnson, & Weber (2014)	3532	0.095 [-0.043, 0.23]	0.16	1
Knobe (2003)	7171	0.58 [-0.052, 1.2]	0.07	1
Tversky & Gati (1978)	2641	0.045 [-0.043, 0.13]	0.28	1

Supplementary Table 50. **Analysis B2**: Mixed effect model, where participants are clustered by their country of origin, where country of origin is based on participants stated hometowns. WEIRDness is operationalizated using CF_{ST}, imputing the CF_{ST} for countries missing cultural distance. A *minimum of 36 participants per country* is used as an inclusion criteria. *p* values are adjusted using Bonferroni correction. The values in brackets represent the 2.5% and 97.5% confidence intervals.

Study	*N*	Effect	*p* value	Adjusted *p* value
Huang, Tse, & Cho (2014)	6091	-24 [-58, 9.8]	0.16	1
Kay, Laurin, Fitzsimons, & Landau (2014)	6020	-0.042 [-0.3, 0.22]	0.74	1
Alter, Oppenheimer, Epley, & Eyre (2007)	6676	-0.055 [-0.26, 0.15]	0.59	1
Graham, Haidt, & Nosek (2009)	6738	-1.3 [-2.5, 0.0095]	0.052	1
Rottenstreich & Hsee (2001)	6720	-0.13 [-0.44, 0.18]	0.39	1
Bauer, Wilkie, Kim, & Bodenhausen (2012)	6159	-0.028 [-0.24, 0.19]	0.8	1
Miyamoto & Kitayama (2002)	6704	0.81 [-0.34, 2]	0.16	1
Inbar, Pizarro, Knobe, & Bloom (2009)	6656	0.078 [-0.94, 1.1]	0.88	1
Critcher & Gilovich (2008)	6319	1.9 [-1.9, 5.8]	0.32	1
Van Lange, Otten, De Bruin, & Joireman (1997)	5790	0.11 [-0.71, 0.93]	0.79	1
Hauser et al. 1/1 (2007)	6707	-0.22 [-0.83, 0.39]	0.47	1
Anderson, Kraus, Galinsky, & Keltner (2012)	6736	-0.038 [-0.15, 0.074]	0.49	1
Ross, Greene, & House (1977)	6527	-3.1 [-7.2, 0.97]	0.13	1
Ross et al. (1977)	6739	2.1 [-2.2, 6.4]	0.33	1
Giessner & Schubert (2007)	7171	0.028 [-0.16, 0.22]	0.76	1
Tversky & Kahneman (1981)	6525	-0.13 [-0.44, 0.18]	0.4	1
Hauser et al. 2/2 (2007)	7200	0.08 [-0.36, 0.51]	0.71	1
Risen & Gilovich (2008)	7265	-0.16 [-0.56, 0.25]	0.43	1
Savani, Markus, Naidu, Kumar, & Berlia (2010)	7272	1.1 [-1.4, 3.7]	0.37	1
Norenzayan, Smith, Kim, & Nisbett (2002)	7277	6 [0.23, 12]	0.042	1
Hsee (1998)	6953	0.16 [-0.11, 0.44]	0.23	1
Gray & Wegner (2009)	7265	0.15 [-0.25, 0.55]	0.45	1
Zhong & Liljenquist (2006)	6353	-0.16 [-0.38, 0.063]	0.15	1
Schwarz, Strack, & Mai (1991)	6801	-0.089 [-0.93, 0.75]	0.83	1
Shafir (1993)	7144	0.029 [-0.36, 0.42]	0.88	1
Zaval, Keenan, Johnson, & Weber (2014)	3706	0.087 [-0.033, 0.21]	0.15	1
Knobe (2003)	7250	0.94 [0.22, 1.7]	0.012	0.34
Tversky & Gati (1978)	3094	0.035 [-0.18, 0.25]	0.74	1

Supplementary Table 51. **Analysis B2**: Mixed effect model, where participants are clustered by their country of origin, where country of origin is based on participants stated hometowns. WEIRDness is operationalizated using CF_{ST}, imputing the CF_{ST} for countries missing cultural distance. A *minimum of 10 participants per country* is used as an inclusion criteria. *p* values are adjusted using Bonferroni correction. The values in brackets represent the 2.5% and 97.5% confidence intervals.

Study	*N*	Effect	*p* value	Adjusted *p* value
Huang, Tse, & Cho (2014)	6283	7.7 [-19, 34]	0.56	1
Kay, Laurin, Fitzsimons, & Landau (2014)	6189	-0.12 [-0.37, 0.13]	0.33	1
Alter, Oppenheimer, Epley, & Eyre (2007)	6843	-0.014 [-0.22, 0.19]	0.89	1
Graham, Haidt, & Nosek (2009)	6907	-0.8 [-1.6, 0.027]	0.058	1
Rottenstreich & Hsee (2001)	6889	0.095 [-0.17, 0.36]	0.47	1
Bauer, Wilkie, Kim, & Bodenhausen (2012)	6328	0.21 [-0.052, 0.46]	0.11	1
Miyamoto & Kitayama (2002)	6872	-0.65 [-2, 0.65]	0.32	1
Inbar, Pizarro, Knobe, & Bloom (2009)	6771	0.033 [-0.71, 0.78]	0.93	1
Critcher & Gilovich (2008)	6461	-2.1 [-5.8, 1.6]	0.25	1
Van Lange, Otten, De Bruin, & Joireman (1997)	5941	0.032 [-0.46, 0.52]	0.9	1
Hauser et al. 1/1 (2007)	6875	3 [0.69, 5.2]	0.012	0.34
Anderson, Kraus, Galinsky, & Keltner (2012)	6905	-0.092 [-0.19, 0.0068]	0.067	1
Ross, Greene, & House (1977)	6681	-2.9 [-6, 0.18]	0.064	1
Ross et al. (1977)	7058	1.1 [-2.8, 5]	0.57	1
Giessner & Schubert (2007)	7468	-0.17 [-0.39, 0.061]	0.15	1
Tversky & Kahneman (1981)	6820	-1 [-3.5, 1.4]	0.4	1
Hauser et al. 2/2 (2007)	7494	0.97 [-0.9, 2.8]	0.3	1
Risen & Gilovich (2008)	7564	-0.15 [-0.61, 0.32]	0.52	1
Savani, Markus, Naidu, Kumar, & Berlia (2010)	7570	1.3 [-2.4, 5.1]	0.48	1
Norenzayan, Smith, Kim, & Nisbett (2002)	7576	4.7 [0.058, 9.3]	0.047	1
Hsee (1998)	7269	0.17 [-0.096, 0.43]	0.21	1
Gray & Wegner (2009)	7563	0.18 [-0.2, 0.56]	0.34	1
Zhong & Liljenquist (2006)	6678	0.023 [-0.24, 0.28]	0.86	1
Schwarz, Strack, & Mai (1991)	7048	-0.11 [-0.63, 0.41]	0.67	1
Shafir (1993)	7466	0.29 [-1.3, 1.9]	0.72	1
Zaval, Keenan, Johnson, & Weber (2014)	3948	0.078 [-0.11, 0.26]	0.4	1
Knobe (2003)	7547	0.62 [0.11, 1.1]	0.018	0.49
Tversky & Gati (1978)	3290	0.078 [-0.062, 0.22]	0.26	1

2.3.3 Analysis C Results

Supplementary Table 52. **Analysis C**: Matrix model, where participants are clustered by source country. WEIRDness is operationalizated using CF_{ST}, where countries missing CF_{ST} are excluded from analysis. A *minimum of 100 participants per country* is used as an inclusion criteria. *p* values are adjusted using Bonferroni correction. The values in brackets represent the 2.5% and 97.5% confidence intervals.

Study	*N*	Effect	*p* value	Adjusted *p* value
Huang, Tse, & Cho (2014)	5920	-1.3	0.97	0.97
Kay, Laurin, Fitzsimons, & Landau (2014)	6014	-0.6	0.049	0.049
Alter, Oppenheimer, Epley, & Eyre (2007)	6696	0.099	0.38	0.38
Graham, Haidt, & Nosek (2009)	6782	-1.4	0.75	0.75
Rottenstreich & Hsee (2001)	6739	0.34	0.25	0.25
Bauer, Wilkie, Kim, & Bodenhausen (2012)	6175	-0.22	0.46	0.46
Miyamoto & Kitayama (2002)	6720	0.3	0.78	0.78
Inbar, Pizarro, Knobe, & Bloom (2009)	6730	-0.78	0.8	0.8
Critcher & Gilovich (2008)	6054	-5.5	0.36	0.36
Van Lange, Otten, De Bruin, & Joireman (1997)	5507	-1.7	0.66	0.66
Hauser et al. 1/1 (2007)	6724	9.1	0.17	0.17
Anderson, Kraus, Galinsky, & Keltner (2012)	6757	0.12	0.18	0.18
Ross, Greene, & House (1977)	6348	3.9	0.48	0.48
Ross et al. (1977)	6454	-7.3	0.13	0.13
Giessner & Schubert (2007)	6902	-0.024	0.91	0.91
Tversky & Kahneman (1981)	6531	-0.14	0.64	0.64
Hauser et al. 2/2 (2007)	7223	0.015	0.98	0.98
Risen & Gilovich (2008)	7197	-0.33	0.31	0.31
Savani, Markus, Naidu, Kumar, & Berlia (2010)	7299	-0.38	0.48	0.48
Norenzayan, Smith, Kim, & Nisbett (2002)	7308	-3.1	0.48	0.48
Hsee (1998)	6993	-0.34	0.11	0.11
Gray & Wegner (2009)	7294	-0.74	0.067	0.067
Zhong & Liljenquist (2006)	6395	-0.14	0.62	0.62
Schwarz, Strack, & Mai (1991)	6438	-1.3	0.51	0.51
Shafir (1993)	7277	0.7	0.029	0.029
Zaval, Keenan, Johnson, & Weber (2014)	3222	0.12	0.78	0.78
Knobe (2003)	7273	-0.92	0.21	0.21
Tversky & Gati (1978)	2600	-0.18	0.77	0.77

Supplementary Table 53. **Analysis C**: Matrix model, where participants are clustered by source country. WEIRDness is operationalizated using CF_{ST}, where countries missing CF_{ST} are excluded from analysis. A *minimum of 50 participants per country* is used as an inclusion criteria. *p* values are adjusted using Bonferroni correction. The values in brackets represent the 2.5% and 97.5% confidence intervals.

Study	*N*	Effect	*p* value	Adjusted *p* value
Huang, Tse, & Cho (2014)	6488	-45	0.24	0.24
Kay, Laurin, Fitzsimons, & Landau (2014)	6411	0.64	0.085	0.085
Alter, Oppenheimer, Epley, & Eyre (2007)	7091	-0.02	0.88	0.88
Graham, Haidt, & Nosek (2009)	7181	-2.7	0.43	0.43
Rottenstreich & Hsee (2001)	7136	0.31	0.3	0.3
Bauer, Wilkie, Kim, & Bodenhausen (2012)	6574	-0.3	0.27	0.27
Miyamoto & Kitayama (2002)	7116	0.13	0.92	0.92
Inbar, Pizarro, Knobe, & Bloom (2009)	7036	-1.5	0.65	0.65
Critcher & Gilovich (2008)	6708	9.4	0.11	0.11
Van Lange, Otten, De Bruin, & Joireman (1997)	6115	-1.9	0.56	0.56
Hauser et al. 1/1 (2007)	7121	-6.1	0.25	0.25
Anderson, Kraus, Galinsky, & Keltner (2012)	7156	0.0088	0.94	0.94
Ross, Greene, & House (1977)	6925	-4.2	0.32	0.32
Ross et al. (1977)	7355	-3.3	0.31	0.31
Giessner & Schubert (2007)	7842	0.034	0.81	0.81
Tversky & Kahneman (1981)	7179	-0.11	0.69	0.69
Hauser et al. 2/2 (2007)	7869	0.0046	0.98	0.98
Risen & Gilovich (2008)	7952	-0.091	0.61	0.61
Savani, Markus, Naidu, Kumar, & Berlia (2010)	7953	0.16	0.89	0.89
Norenzayan, Smith, Kim, & Nisbett (2002)	7968	-2.7	0.37	0.37
Hsee (1998)	7642	-0.19	0.18	0.18
Gray & Wegner (2009)	7953	-0.055	0.88	0.88
Zhong & Liljenquist (2006)	6938	0.0052	0.98	0.98
Schwarz, Strack, & Mai (1991)	7413	2.2	0.045	0.045
Shafir (1993)	7852	-0.15	0.45	0.45
Zaval, Keenan, Johnson, & Weber (2014)	4041	-0.076	0.63	0.63
Knobe (2003)	7933	-0.7	0.12	0.12
Tversky & Gati (1978)	3003	-0.38	0.22	0.22

Supplementary Table 54. **Analysis C**: Matrix model, where participants are clustered by source country. WEIRDness is operationalizated using CF_{ST}, where countries missing CF_{ST} are excluded from analysis. A *minimum of 36 participants per country* is used as an inclusion criteria. *p* values are adjusted using Bonferroni correction. The values in brackets represent the 2.5% and 97.5% confidence intervals.

Study	*N*	Effect	*p* value	Adjusted *p* value
Huang, Tse, & Cho (2014)	6537	-11	0.78	0.78
Kay, Laurin, Fitzsimons, & Landau (2014)	6455	0.21	0.55	0.55
Alter, Oppenheimer, Epley, & Eyre (2007)	7135	0.12	0.35	0.35
Graham, Haidt, & Nosek (2009)	7261	-4.6	0.23	0.23
Rottenstreich & Hsee (2001)	7216	0.27	0.43	0.43
Bauer, Wilkie, Kim, & Bodenhausen (2012)	6654	0.16	0.55	0.55
Miyamoto & Kitayama (2002)	7160	0.09	0.95	0.95
Inbar, Pizarro, Knobe, & Bloom (2009)	7080	-3.3	0.28	0.28
Critcher & Gilovich (2008)	6752	0.2	0.97	0.97
Van Lange, Otten, De Bruin, & Joireman (1997)	6206	1.1	0.7	0.7
Hauser et al. 1/1 (2007)	7201	-3.7	0.58	0.58
Anderson, Kraus, Galinsky, & Keltner (2012)	7236	-0.0018	0.99	0.99
Ross, Greene, & House (1977)	6968	-3.1	0.42	0.42
Ross et al. (1977)	7440	-3	0.25	0.25
Giessner & Schubert (2007)	7889	-0.029	0.79	0.79
Tversky & Kahneman (1981)	7227	-0.31	0.29	0.29
Hauser et al. 2/2 (2007)	7917	-0.1	0.6	0.6
Risen & Gilovich (2008)	7999	0.0038	0.99	0.99
Savani, Markus, Naidu, Kumar, & Berlia (2010)	8001	0.7	0.53	0.53
Norenzayan, Smith, Kim, & Nisbett (2002)	8016	-2.4	0.51	0.51
Hsee (1998)	7690	-0.15	0.58	0.58
Gray & Wegner (2009)	8001	0.22	0.51	0.51
Zhong & Liljenquist (2006)	7022	0.17	0.26	0.26
Schwarz, Strack, & Mai (1991)	7459	0.36	0.74	0.74
Shafir (1993)	7900	0.33	0.11	0.11
Zaval, Keenan, Johnson, & Weber (2014)	4133	0.067	0.67	0.67
Knobe (2003)	7981	-0.24	0.62	0.62
Tversky & Gati (1978)	3381	-0.089	0.68	0.68

Supplementary Table 55. **Analysis C**: Matrix model, where participants are clustered by source country. WEIRDness is operationalizated using CF_{ST}, where countries missing CF_{ST} are excluded from analysis. A *minimum of 10 participants per country* is used as an inclusion criteria. *p* values are adjusted using Bonferroni correction. The values in brackets represent the 2.5% and 97.5% confidence intervals.

Study	*N*	Effect	*p* value	Adjusted *p* value
Huang, Tse, & Cho (2014)	6587	5.2	0.88	0.88
Kay, Laurin, Fitzsimons, & Landau (2014)	6490	0.21	0.54	0.54
Alter, Oppenheimer, Epley, & Eyre (2007)	7170	0.12	0.32	0.32
Graham, Haidt, & Nosek (2009)	7261	-4.6	0.25	0.25
Rottenstreich & Hsee (2001)	7216	0.27	0.4	0.4
Bauer, Wilkie, Kim, & Bodenhausen (2012)	6654	0.16	0.57	0.57
Miyamoto & Kitayama (2002)	7195	0.09	0.96	0.96
Inbar, Pizarro, Knobe, & Bloom (2009)	7115	-3.3	0.28	0.28
Critcher & Gilovich (2008)	6785	0.2	0.97	0.97
Van Lange, Otten, De Bruin, & Joireman (1997)	6239	1.1	0.72	0.72
Hauser et al. 1/1 (2007)	7201	-3.7	0.57	0.57
Anderson, Kraus, Galinsky, & Keltner (2012)	7236	-0.0018	0.99	0.99
Ross, Greene, & House (1977)	7002	-3.1	0.41	0.41
Ross et al. (1977)	7440	-3	0.24	0.24
Giessner & Schubert (2007)	7889	-0.029	0.8	0.8
Tversky & Kahneman (1981)	7227	-0.31	0.28	0.28
Hauser et al. 2/2 (2007)	7917	-0.1	0.6	0.6
Risen & Gilovich (2008)	7999	0.0038	0.98	0.98
Savani, Markus, Naidu, Kumar, & Berlia (2010)	8001	0.7	0.52	0.52
Norenzayan, Smith, Kim, & Nisbett (2002)	8016	-2.4	0.54	0.54
Hsee (1998)	7690	-0.15	0.56	0.56
Gray & Wegner (2009)	8001	0.22	0.49	0.49
Zhong & Liljenquist (2006)	7056	0.032	0.82	0.82
Schwarz, Strack, & Mai (1991)	7459	0.36	0.75	0.75
Shafir (1993)	7900	0.33	0.12	0.12
Zaval, Keenan, Johnson, & Weber (2014)	4202	0.059	0.74	0.74
Knobe (2003)	7981	-0.24	0.6	0.6
Tversky & Gati (1978)	3549	-0.042	0.69	0.69

Supplementary Table 56. **Analysis C**: Matrix model, where participants are clustered by source country. WEIRDness is operationalizated using CF_{ST}, imputing the CF_{ST} for countries missing cultural distance. A *minimum of 100 participants per country* is used as an inclusion criteria. *p* values are adjusted using Bonferroni correction. The values in brackets represent the 2.5% and 97.5% confidence intervals.

Study	*N*	Effect	*p* value	Adjusted *p* value
Huang, Tse, & Cho (2014)	5920	-26	0.51	0.51
Kay, Laurin, Fitzsimons, & Landau (2014)	6014	-0.26	0.5	0.5
Alter, Oppenheimer, Epley, & Eyre (2007)	6696	0.074	0.48	0.48
Graham, Haidt, & Nosek (2009)	6782	5.8	0.096	0.096
Rottenstreich & Hsee (2001)	6739	-0.16	0.56	0.56
Bauer, Wilkie, Kim, & Bodenhausen (2012)	6175	-0.2	0.5	0.5
Miyamoto & Kitayama (2002)	6720	-1.2	0.28	0.28
Inbar, Pizarro, Knobe, & Bloom (2009)	6730	3.7	0.2	0.2
Critcher & Gilovich (2008)	6054	0.76	0.92	0.92
Van Lange, Otten, De Bruin, & Joireman (1997)	5507	2	0.55	0.55
Hauser et al. 1/1 (2007)	6724	-4.2	0.41	0.41
Anderson, Kraus, Galinsky, & Keltner (2012)	6757	-0.058	0.53	0.53
Ross, Greene, & House (1977)	6348	-0.22	0.97	0.97
Ross et al. (1977)	6454	5.6	0.37	0.37
Giessner & Schubert (2007)	6902	-0.31	0.38	0.38
Tversky & Kahneman (1981)	6531	0.099	0.64	0.64
Hauser et al. 2/2 (2007)	7223	-0.024	0.92	0.92
Risen & Gilovich (2008)	7197	0.14	0.56	0.56
Savani, Markus, Naidu, Kumar, & Berlia (2010)	7299	0.45	0.24	0.24
Norenzayan, Smith, Kim, & Nisbett (2002)	7308	10	0.018	0.018
Hsee (1998)	6993	-0.52	0.005	0.005
Gray & Wegner (2009)	7294	-0.015	0.96	0.96
Zhong & Liljenquist (2006)	6395	0.2	0.34	0.34
Schwarz, Strack, & Mai (1991)	6438	2.9	0.22	0.22
Shafir (1993)	7277	0.02	0.93	0.93
Zaval, Keenan, Johnson, & Weber (2014)	3222	0.12	0.78	0.78
Knobe (2003)	7273	0.24	0.69	0.69
Tversky & Gati (1978)	2600	-0.18	0.75	0.75

Supplementary Table 57. **Analysis C**: Matrix model, where participants are clustered by source country. WEIRDness is operationalizated using CF_{ST}, imputing the CF_{ST} for countries missing cultural distance. A *minimum of 50 participants per country* is used as an inclusion criteria. *p* values are adjusted using Bonferroni correction. The values in brackets represent the 2.5% and 97.5% confidence intervals.

Study	*N*	Effect	*p* value	Adjusted *p* value
Huang, Tse, & Cho (2014)	6488	71	0.032	0.032
Kay, Laurin, Fitzsimons, & Landau (2014)	6411	0.55	0.13	0.13
Alter, Oppenheimer, Epley, & Eyre (2007)	7091	-0.017	0.86	0.86
Graham, Haidt, & Nosek (2009)	7181	-0.2	0.93	0.93
Rottenstreich & Hsee (2001)	7136	-0.39	0.13	0.13
Bauer, Wilkie, Kim, & Bodenhausen (2012)	6574	-0.072	0.75	0.75
Miyamoto & Kitayama (2002)	7116	-0.69	0.51	0.51
Inbar, Pizarro, Knobe, & Bloom (2009)	7036	-2.2	0.4	0.4
Critcher & Gilovich (2008)	6708	1.1	0.79	0.79
Van Lange, Otten, De Bruin, & Joireman (1997)	6115	0.85	0.74	0.74
Hauser et al. 1/1 (2007)	7121	2.9	0.58	0.58
Anderson, Kraus, Galinsky, & Keltner (2012)	7156	-0.023	0.77	0.77
Ross, Greene, & House (1977)	6925	-0.56	0.86	0.86
Ross et al. (1977)	7355	6.1	0.11	0.11
Giessner & Schubert (2007)	7842	0.4	0.019	0.019
Tversky & Kahneman (1981)	7179	-0.28	0.24	0.24
Hauser et al. 2/2 (2007)	7869	-0.01	0.95	0.95
Risen & Gilovich (2008)	7952	0.02	0.89	0.89
Savani, Markus, Naidu, Kumar, & Berlia (2010)	7953	0.21	0.84	0.84
Norenzayan, Smith, Kim, & Nisbett (2002)	7968	5.5	0.087	0.087
Hsee (1998)	7642	-0.12	0.39	0.39
Gray & Wegner (2009)	7953	0.3	0.27	0.27
Zhong & Liljenquist (2006)	6938	-0.031	0.85	0.85
Schwarz, Strack, & Mai (1991)	7413	-0.32	0.75	0.75
Shafir (1993)	7852	-0.11	0.51	0.51
Zaval, Keenan, Johnson, & Weber (2014)	4041	-0.031	0.8	0.8
Knobe (2003)	7933	0.27	0.44	0.44
Tversky & Gati (1978)	3003	0.34	0.28	0.28

Supplementary Table 58. **Analysis C**: Matrix model, where participants are clustered by source country. WEIRDness is operationalizated using CF_{ST}, imputing the CF_{ST} for countries missing cultural distance. A *minimum of 36 participants per country* is used as an inclusion criteria. *p* values are adjusted using Bonferroni correction. The values in brackets represent the 2.5% and 97.5% confidence intervals.

Study	*N*	Effect	*p* value	Adjusted *p* value
Huang, Tse, & Cho (2014)	6537	59	0.043	0.043
Kay, Laurin, Fitzsimons, & Landau (2014)	6455	0.51	0.11	0.11
Alter, Oppenheimer, Epley, & Eyre (2007)	7135	-0.044	0.62	0.62
Graham, Haidt, & Nosek (2009)	7261	-1.1	0.69	0.69
Rottenstreich & Hsee (2001)	7216	-0.21	0.41	0.41
Bauer, Wilkie, Kim, & Bodenhausen (2012)	6654	0.1	0.63	0.63
Miyamoto & Kitayama (2002)	7160	-0.74	0.53	0.53
Inbar, Pizarro, Knobe, & Bloom (2009)	7080	-2.6	0.28	0.28
Critcher & Gilovich (2008)	6752	-2.5	0.56	0.56
Van Lange, Otten, De Bruin, & Joireman (1997)	6206	4.1	0.14	0.14
Hauser et al. 1/1 (2007)	7201	0.65	0.87	0.87
Anderson, Kraus, Galinsky, & Keltner (2012)	7236	-0.1	0.14	0.14
Ross, Greene, & House (1977)	6968	-2.7	0.42	0.42
Ross et al. (1977)	7440	-0.41	0.89	0.89
Giessner & Schubert (2007)	7889	0.4	0.014	0.014
Tversky & Kahneman (1981)	7227	-0.14	0.53	0.53
Hauser et al. 2/2 (2007)	7917	0.4	0.03	0.03
Risen & Gilovich (2008)	7999	-0.048	0.77	0.77
Savani, Markus, Naidu, Kumar, & Berlia (2010)	8001	2.6	0.025	0.025
Norenzayan, Smith, Kim, & Nisbett (2002)	8016	0.13	0.96	0.96
Hsee (1998)	7690	-0.11	0.64	0.64
Gray & Wegner (2009)	8001	0.52	0.087	0.087
Zhong & Liljenquist (2006)	7022	0.26	0.058	0.058
Schwarz, Strack, & Mai (1991)	7459	0.95	0.33	0.33
Shafir (1993)	7900	-0.13	0.47	0.47
Zaval, Keenan, Johnson, & Weber (2014)	4133	0.055	0.59	0.59
Knobe (2003)	7981	0.3	0.44	0.44
Tversky & Gati (1978)	3381	0.069	0.66	0.66

Supplementary Table 59. **Analysis C**: Matrix model, where participants are clustered by source country. WEIRDness is operationalizated using CF_{ST}, imputing the CF_{ST} for countries missing cultural distance. A *minimum of 10 participants per country* is used as an inclusion criteria. *p* values are adjusted using Bonferroni correction. The values in brackets represent the 2.5% and 97.5% confidence intervals.

Study	*N*	Effect	*p* value	Adjusted *p* value
Huang, Tse, & Cho (2014)	6587	-3.6	0.88	0.88
Kay, Laurin, Fitzsimons, & Landau (2014)	6490	0.35	0.27	0.27
Alter, Oppenheimer, Epley, & Eyre (2007)	7170	-0.099	0.33	0.33
Graham, Haidt, & Nosek (2009)	7261	-1.1	0.69	0.69
Rottenstreich & Hsee (2001)	7216	-0.21	0.37	0.37
Bauer, Wilkie, Kim, & Bodenhausen (2012)	6654	0.1	0.62	0.62
Miyamoto & Kitayama (2002)	7195	-2.8	0.035	0.035
Inbar, Pizarro, Knobe, & Bloom (2009)	7115	-0.17	0.96	0.96
Critcher & Gilovich (2008)	6785	-8.1	0.1	0.1
Van Lange, Otten, De Bruin, & Joireman (1997)	6239	3.5	0.18	0.18
Hauser et al. 1/1 (2007)	7201	0.65	0.86	0.86
Anderson, Kraus, Galinsky, & Keltner (2012)	7236	-0.1	0.13	0.13
Ross, Greene, & House (1977)	7002	-2.3	0.53	0.53
Ross et al. (1977)	7440	-0.41	0.88	0.88
Giessner & Schubert (2007)	7889	0.4	0.02	0.02
Tversky & Kahneman (1981)	7227	-0.14	0.55	0.55
Hauser et al. 2/2 (2007)	7917	0.4	0.02	0.02
Risen & Gilovich (2008)	7999	-0.048	0.77	0.77
Savani, Markus, Naidu, Kumar, & Berlia (2010)	8001	2.6	0.013	0.013
Norenzayan, Smith, Kim, & Nisbett (2002)	8016	0.13	0.97	0.97
Hsee (1998)	7690	-0.11	0.66	0.66
Gray & Wegner (2009)	8001	0.52	0.1	0.1
Zhong & Liljenquist (2006)	7056	-0.0033	0.97	0.97
Schwarz, Strack, & Mai (1991)	7459	0.95	0.3	0.3
Shafir (1993)	7900	-0.13	0.46	0.46
Zaval, Keenan, Johnson, & Weber (2014)	4202	-0.053	0.67	0.67
Knobe (2003)	7981	0.3	0.46	0.46
Tversky & Gati (1978)	3549	-0.022	0.87	0.87

Supplementary Table 60. **Analysis C**: Matrix model, where participants are clustered by birth country and participants missing birth countries are excluded from analysis. WEIRDness is operationalizated using CF_{ST}, where countries missing CF_{ST} are excluded from analysis. A *minimum of 100 participants per country* is used as an inclusion criteria. *p* values are adjusted using Bonferroni correction. The values in brackets represent the 2.5% and 97.5% confidence intervals.

Study	*N*	Effect	*p* value	Adjusted *p* value
Huang, Tse, & Cho (2014)	5210	-41	0.35	0.35
Kay, Laurin, Fitzsimons, & Landau (2014)	5277	0.39	0.21	0.21
Alter, Oppenheimer, Epley, & Eyre (2007)	5817	-0.1	0.24	0.24
Graham, Haidt, & Nosek (2009)	5870	0.44	0.88	0.88
Rottenstreich & Hsee (2001)	5856	-0.27	0.56	0.56
Bauer, Wilkie, Kim, & Bodenhausen (2012)	5405	-0.21	0.45	0.45
Miyamoto & Kitayama (2002)	5844	1.7	0.32	0.32
Inbar, Pizarro, Knobe, & Bloom (2009)	5839	-0.011	0.99	0.99
Critcher & Gilovich (2008)	5224	3.9	0.16	0.16
Van Lange, Otten, De Bruin, & Joireman (1997)	4792	0.014	0.99	0.99
Hauser et al. 1/1 (2007)	5748	1.7	0.063	0.063
Anderson, Kraus, Galinsky, & Keltner (2012)	5869	0.15	0.12	0.12
Ross, Greene, & House (1977)	5595	-1.9	0.71	0.71
Ross et al. (1977)	5139	-2.5	0.66	0.66
Giessner & Schubert (2007)	5591	0.15	0.54	0.54
Tversky & Kahneman (1981)	4843	-0.094	0.84	0.84
Hauser et al. 2/2 (2007)	5613	-0.088	0.8	0.8
Risen & Gilovich (2008)	5655	0.2	0.69	0.69
Savani, Markus, Naidu, Kumar, & Berlia (2010)	5664	-0.095	0.73	0.73
Norenzayan, Smith, Kim, & Nisbett (2002)	5661	1.8	0.8	0.8
Hsee (1998)	5460	0.01	0.97	0.97
Gray & Wegner (2009)	5653	0.24	0.69	0.69
Zhong & Liljenquist (2006)	5228	-0.34	0.1	0.1
Schwarz, Strack, & Mai (1991)	5337	-0.38	0.78	0.78
Shafir (1993)	5643	0.12	0.69	0.69
Zaval, Keenan, Johnson, & Weber (2014)	2602	0.025	0.95	0.95
Knobe (2003)	5638	0.77	0.4	0.4
Tversky & Gati (1978)	2015	-0.025	0.95	0.95

Supplementary Table 61. **Analysis C**: Matrix model, where participants are clustered by birth country and participants missing birth countries are excluded from analysis. WEIRDness is operationalizated using CF_{ST}, where countries missing CF_{ST} are excluded from analysis. A *minimum of 50 participants per country* is used as an inclusion criteria. *p* values are adjusted using Bonferroni correction. The values in brackets represent the 2.5% and 97.5% confidence intervals.

Study	*N*	Effect	*p* value	Adjusted *p* value
Huang, Tse, & Cho (2014)	5674	-47	0.28	0.28
Kay, Laurin, Fitzsimons, & Landau (2014)	5595	0.33	0.25	0.25
Alter, Oppenheimer, Epley, & Eyre (2007)	6137	-0.15	0.21	0.21
Graham, Haidt, & Nosek (2009)	6191	0.28	0.91	0.91
Rottenstreich & Hsee (2001)	6175	-0.28	0.5	0.5
Bauer, Wilkie, Kim, & Bodenhausen (2012)	5724	-0.11	0.68	0.68
Miyamoto & Kitayama (2002)	6162	1.9	0.19	0.19
Inbar, Pizarro, Knobe, & Bloom (2009)	6158	-0.14	0.9	0.9
Critcher & Gilovich (2008)	5807	5.8	0.044	0.044
Van Lange, Otten, De Bruin, & Joireman (1997)	5382	0.0071	1	1
Hauser et al. 1/1 (2007)	6166	1.6	0.053	0.053
Anderson, Kraus, Galinsky, & Keltner (2012)	6190	0.097	0.42	0.42
Ross, Greene, & House (1977)	6005	0.39	0.94	0.94
Ross et al. (1977)	5966	-0.98	0.81	0.81
Giessner & Schubert (2007)	6259	0.12	0.5	0.5
Tversky & Kahneman (1981)	5620	-0.32	0.41	0.41
Hauser et al. 2/2 (2007)	6289	0.23	0.61	0.61
Risen & Gilovich (2008)	6336	0.1	0.84	0.84
Savani, Markus, Naidu, Kumar, & Berlia (2010)	6345	-0.58	0.19	0.19
Norenzayan, Smith, Kim, & Nisbett (2002)	6344	3.4	0.58	0.58
Hsee (1998)	6137	-0.2	0.57	0.57
Gray & Wegner (2009)	6333	-0.14	0.76	0.76
Zhong & Liljenquist (2006)	5846	-0.13	0.66	0.66
Schwarz, Strack, & Mai (1991)	5961	-0.49	0.7	0.7
Shafir (1993)	6226	-0.16	0.63	0.63
Zaval, Keenan, Johnson, & Weber (2014)	3165	0.026	0.89	0.89
Knobe (2003)	6321	0.7	0.39	0.39
Tversky & Gati (1978)	2384	-0.11	0.57	0.57

Supplementary Table 62. **Analysis C**: Matrix model, where participants are clustered by birth country and participants missing birth countries are excluded from analysis. WEIRDness is operationalizated using CF_{ST}, where countries missing CF_{ST} are excluded from analysis. A *minimum of 36 participants per country* is used as an inclusion criteria. *p* values are adjusted using Bonferroni correction. The values in brackets represent the 2.5% and 97.5% confidence intervals.

Study	*N*	Effect	*p* value	Adjusted *p* value
Huang, Tse, & Cho (2014)	5714	-42	0.31	0.31
Kay, Laurin, Fitzsimons, & Landau (2014)	5728	0.19	0.59	0.59
Alter, Oppenheimer, Epley, & Eyre (2007)	6270	-0.43	0.15	0.15
Graham, Haidt, & Nosek (2009)	6325	-0.28	0.91	0.91
Rottenstreich & Hsee (2001)	6309	-0.36	0.32	0.32
Bauer, Wilkie, Kim, & Bodenhausen (2012)	5858	-0.095	0.72	0.72
Miyamoto & Kitayama (2002)	6296	1.6	0.22	0.22
Inbar, Pizarro, Knobe, & Bloom (2009)	6292	-0.38	0.76	0.76
Critcher & Gilovich (2008)	5938	2.7	0.53	0.53
Van Lange, Otten, De Bruin, & Joireman (1997)	5511	-0.45	0.7	0.7
Hauser et al. 1/1 (2007)	6299	1	0.22	0.22
Anderson, Kraus, Galinsky, & Keltner (2012)	6324	-0.028	0.89	0.89
Ross, Greene, & House (1977)	6137	-5.7	0.48	0.48
Ross et al. (1977)	5966	-0.98	0.8	0.8
Giessner & Schubert (2007)	6295	0.092	0.61	0.61
Tversky & Kahneman (1981)	5657	-0.25	0.55	0.55
Hauser et al. 2/2 (2007)	6325	0.11	0.79	0.79
Risen & Gilovich (2008)	6373	0.089	0.85	0.85
Savani, Markus, Naidu, Kumar, & Berlia (2010)	6382	3.4	0.55	0.55
Norenzayan, Smith, Kim, & Nisbett (2002)	6382	1.4	0.83	0.83
Hsee (1998)	6174	-0.2	0.47	0.47
Gray & Wegner (2009)	6371	-0.23	0.54	0.54
Zhong & Liljenquist (2006)	5846	-0.13	0.67	0.67
Schwarz, Strack, & Mai (1991)	5998	-0.51	0.64	0.64
Shafir (1993)	6262	0.066	0.89	0.89
Zaval, Keenan, Johnson, & Weber (2014)	3302	-0.019	0.92	0.92
Knobe (2003)	6359	0.7	0.34	0.34
Tversky & Gati (1978)	2719	-0.054	0.85	0.85

Supplementary Table 63. **Analysis C**: Matrix model, where participants are clustered by birth country and participants missing birth countries are excluded from analysis. WEIRDness is operationalizated using CF_{ST}, where countries missing CF_{ST} are excluded from analysis. A *minimum of 10 participants per country* is used as an inclusion criteria. *p* values are adjusted using Bonferroni correction. The values in brackets represent the 2.5% and 97.5% confidence intervals.

Study	*N*	Effect	*p* value	Adjusted *p* value
Huang, Tse, & Cho (2014)	5859	53	0.031	0.031
Kay, Laurin, Fitzsimons, & Landau (2014)	5860	0.37	0.38	0.38
Alter, Oppenheimer, Epley, & Eyre (2007)	6402	-0.45	0.2	0.2
Graham, Haidt, & Nosek (2009)	6457	-1.2	0.3	0.3
Rottenstreich & Hsee (2001)	6441	0.49	0.094	0.094
Bauer, Wilkie, Kim, & Bodenhausen (2012)	5990	1.3	0.042	0.042
Miyamoto & Kitayama (2002)	6428	4.9	0.026	0.026
Inbar, Pizarro, Knobe, & Bloom (2009)	6381	-0.31	0.74	0.74
Critcher & Gilovich (2008)	6046	8.2	0.15	0.15
Van Lange, Otten, De Bruin, & Joireman (1997)	5629	-0.44	0.49	0.49
Hauser et al. 1/1 (2007)	6430	9.4	0.07	0.07
Anderson, Kraus, Galinsky, & Keltner (2012)	6456	0.38	0.04	0.04
Ross, Greene, & House (1977)	6256	-5	0.26	0.26
Ross et al. (1977)	6306	2.3	0.66	0.66
Giessner & Schubert (2007)	6641	-0.083	0.87	0.87
Tversky & Kahneman (1981)	6000	1.3	0.82	0.82
Hauser et al. 2/2 (2007)	6668	-4.6	0.23	0.23
Risen & Gilovich (2008)	6722	-0.13	0.8	0.8
Savani, Markus, Naidu, Kumar, & Berlia (2010)	6722	-3	0.71	0.71
Norenzayan, Smith, Kim, & Nisbett (2002)	6732	5.2	0.33	0.33
Hsee (1998)	6537	-0.097	0.72	0.72
Gray & Wegner (2009)	6720	-0.43	0.35	0.35
Zhong & Liljenquist (2006)	6173	-0.43	0.22	0.22
Schwarz, Strack, & Mai (1991)	6303	0.39	0.49	0.49
Shafir (1993)	6630	-2.3	0.4	0.4
Zaval, Keenan, Johnson, & Weber (2014)	3560	-0.097	0.75	0.75
Knobe (2003)	6707	1.1	0.045	0.045
Tversky & Gati (1978)	2911	-0.0037	0.99	0.99

Supplementary Table 64. **Analysis C**: Matrix model, where participants are clustered by birth country and participants missing birth countries are excluded from analysis. WEIRDness is operationalizated using CF_{ST}, imputing the CF_{ST} for countries missing cultural distance. A *minimum of 100 participants per country* is used as an inclusion criteria. *p* values are adjusted using Bonferroni correction. The values in brackets represent the 2.5% and 97.5% confidence intervals.

Study	*N*	Effect	*p* value	Adjusted *p* value
Huang, Tse, & Cho (2014)	5210	-41	0.36	0.36
Kay, Laurin, Fitzsimons, & Landau (2014)	5277	0.39	0.2	0.2
Alter, Oppenheimer, Epley, & Eyre (2007)	5817	-0.1	0.27	0.27
Graham, Haidt, & Nosek (2009)	5870	0.44	0.9	0.9
Rottenstreich & Hsee (2001)	5856	-0.27	0.56	0.56
Bauer, Wilkie, Kim, & Bodenhausen (2012)	5405	-0.21	0.43	0.43
Miyamoto & Kitayama (2002)	5844	1.7	0.32	0.32
Inbar, Pizarro, Knobe, & Bloom (2009)	5839	-0.011	0.99	0.99
Critcher & Gilovich (2008)	5224	3.9	0.16	0.16
Van Lange, Otten, De Bruin, & Joireman (1997)	4792	0.014	0.99	0.99
Hauser et al. 1/1 (2007)	5748	1.7	0.069	0.069
Anderson, Kraus, Galinsky, & Keltner (2012)	5869	0.15	0.15	0.15
Ross, Greene, & House (1977)	5595	-1.9	0.7	0.7
Ross et al. (1977)	5139	-2.5	0.66	0.66
Giessner & Schubert (2007)	5591	0.15	0.53	0.53
Tversky & Kahneman (1981)	4843	-0.094	0.82	0.82
Hauser et al. 2/2 (2007)	5613	-0.088	0.77	0.77
Risen & Gilovich (2008)	5655	0.2	0.7	0.7
Savani, Markus, Naidu, Kumar, & Berlia (2010)	5664	-0.095	0.67	0.67
Norenzayan, Smith, Kim, & Nisbett (2002)	5661	1.8	0.79	0.79
Hsee (1998)	5460	0.01	0.97	0.97
Gray & Wegner (2009)	5653	0.24	0.67	0.67
Zhong & Liljenquist (2006)	5228	-0.34	0.12	0.12
Schwarz, Strack, & Mai (1991)	5337	-0.38	0.76	0.76
Shafir (1993)	5643	0.12	0.69	0.69
Zaval, Keenan, Johnson, & Weber (2014)	2602	0.025	0.95	0.95
Knobe (2003)	5638	0.77	0.39	0.39
Tversky & Gati (1978)	2015	-0.025	0.94	0.94

Supplementary Table 65. **Analysis C**: Matrix model, where participants are clustered by birth country and participants missing birth countries are excluded from analysis. WEIRDness is operationalizated using CF_{ST}, imputing the CF_{ST} for countries missing cultural distance. A *minimum of 50 participants per country* is used as an inclusion criteria. *p* values are adjusted using Bonferroni correction. The values in brackets represent the 2.5% and 97.5% confidence intervals.

Study	*N*	Effect	*p* value	Adjusted *p* value
Huang, Tse, & Cho (2014)	5674	-47	0.29	0.29
Kay, Laurin, Fitzsimons, & Landau (2014)	5595	0.33	0.25	0.25
Alter, Oppenheimer, Epley, & Eyre (2007)	6137	-0.15	0.23	0.23
Graham, Haidt, & Nosek (2009)	6191	0.28	0.91	0.91
Rottenstreich & Hsee (2001)	6175	-0.28	0.49	0.49
Bauer, Wilkie, Kim, & Bodenhausen (2012)	5724	-0.11	0.63	0.63
Miyamoto & Kitayama (2002)	6162	1.9	0.2	0.2
Inbar, Pizarro, Knobe, & Bloom (2009)	6158	-0.14	0.88	0.88
Critcher & Gilovich (2008)	5807	5.8	0.067	0.067
Van Lange, Otten, De Bruin, & Joireman (1997)	5382	0.0071	0.99	0.99
Hauser et al. 1/1 (2007)	6166	1.6	0.054	0.054
Anderson, Kraus, Galinsky, & Keltner (2012)	6190	0.097	0.4	0.4
Ross, Greene, & House (1977)	6005	0.39	0.93	0.93
Ross et al. (1977)	5966	-0.98	0.8	0.8
Giessner & Schubert (2007)	6259	0.12	0.51	0.51
Tversky & Kahneman (1981)	5620	-0.32	0.43	0.43
Hauser et al. 2/2 (2007)	6289	0.23	0.59	0.59
Risen & Gilovich (2008)	6336	0.1	0.85	0.85
Savani, Markus, Naidu, Kumar, & Berlia (2010)	6345	-0.58	0.18	0.18
Norenzayan, Smith, Kim, & Nisbett (2002)	6344	3.4	0.57	0.57
Hsee (1998)	6137	-0.2	0.55	0.55
Gray & Wegner (2009)	6333	-0.14	0.75	0.75
Zhong & Liljenquist (2006)	5846	-0.13	0.68	0.68
Schwarz, Strack, & Mai (1991)	5961	-0.49	0.7	0.7
Shafir (1993)	6226	-0.16	0.63	0.63
Zaval, Keenan, Johnson, & Weber (2014)	3165	0.026	0.89	0.89
Knobe (2003)	6321	0.7	0.4	0.4
Tversky & Gati (1978)	2384	-0.11	0.61	0.61

Supplementary Table 66. **Analysis C**: Matrix model, where participants are clustered by birth country and participants missing birth countries are excluded from analysis. WEIRDness is operationalizated using CF_{ST}, imputing the CF_{ST} for countries missing cultural distance. A *minimum of 36 participants per country* is used as an inclusion criteria. *p* values are adjusted using Bonferroni correction. The values in brackets represent the 2.5% and 97.5% confidence intervals.

Study	*N*	Effect	*p* value	Adjusted *p* value
Huang, Tse, & Cho (2014)	5714	-42	0.34	0.34
Kay, Laurin, Fitzsimons, & Landau (2014)	5728	0.19	0.61	0.61
Alter, Oppenheimer, Epley, & Eyre (2007)	6270	-0.43	0.16	0.16
Graham, Haidt, & Nosek (2009)	6325	-0.28	0.91	0.91
Rottenstreich & Hsee (2001)	6309	-0.36	0.32	0.32
Bauer, Wilkie, Kim, & Bodenhausen (2012)	5858	-0.095	0.77	0.77
Miyamoto & Kitayama (2002)	6296	1.6	0.24	0.24
Inbar, Pizarro, Knobe, & Bloom (2009)	6292	-0.38	0.76	0.76
Critcher & Gilovich (2008)	5938	2.7	0.52	0.52
Van Lange, Otten, De Bruin, & Joireman (1997)	5511	-0.45	0.71	0.71
Hauser et al. 1/1 (2007)	6299	1	0.2	0.2
Anderson, Kraus, Galinsky, & Keltner (2012)	6324	-0.028	0.88	0.88
Ross, Greene, & House (1977)	6137	-5.7	0.46	0.46
Ross et al. (1977)	5966	-0.98	0.78	0.78
Giessner & Schubert (2007)	6295	0.092	0.62	0.62
Tversky & Kahneman (1981)	5657	-0.25	0.57	0.57
Hauser et al. 2/2 (2007)	6325	0.11	0.78	0.78
Risen & Gilovich (2008)	6373	0.089	0.83	0.83
Savani, Markus, Naidu, Kumar, & Berlia (2010)	6382	3.4	0.52	0.52
Norenzayan, Smith, Kim, & Nisbett (2002)	6382	1.4	0.83	0.83
Hsee (1998)	6174	-0.2	0.52	0.52
Gray & Wegner (2009)	6371	-0.23	0.58	0.58
Zhong & Liljenquist (2006)	5846	-0.13	0.66	0.66
Schwarz, Strack, & Mai (1991)	5998	-0.51	0.61	0.61
Shafir (1993)	6262	0.066	0.9	0.9
Zaval, Keenan, Johnson, & Weber (2014)	3302	-0.019	0.9	0.9
Knobe (2003)	6359	0.7	0.37	0.37
Tversky & Gati (1978)	2719	-0.054	0.84	0.84

Supplementary Table 67. **Analysis C**: Matrix model, where participants are clustered by birth country and participants missing birth countries are excluded from analysis. WEIRDness is operationalizated using CF_{ST}, imputing the CF_{ST} for countries missing cultural distance. A *minimum of 10 participants per countr*y is used as an inclusion criteria. *p* values are adjusted using Bonferroni correction. The values in brackets represent the 2.5% and 97.5% confidence intervals.

Study	*N*	Effect	*p* value	Adjusted *p* value
Huang, Tse, & Cho (2014)	5859	53	0.025	0.025
Kay, Laurin, Fitzsimons, & Landau (2014)	5860	0.37	0.41	0.41
Alter, Oppenheimer, Epley, & Eyre (2007)	6402	-0.45	0.21	0.21
Graham, Haidt, & Nosek (2009)	6457	-1.2	0.28	0.28
Rottenstreich & Hsee (2001)	6441	0.49	0.084	0.084
Bauer, Wilkie, Kim, & Bodenhausen (2012)	5990	1.3	0.036	0.036
Miyamoto & Kitayama (2002)	6428	4.9	0.013	0.013
Inbar, Pizarro, Knobe, & Bloom (2009)	6381	-0.31	0.78	0.78
Critcher & Gilovich (2008)	6046	8.2	0.13	0.13
Van Lange, Otten, De Bruin, & Joireman (1997)	5629	-0.44	0.49	0.49
Hauser et al. 1/1 (2007)	6430	9.4	0.079	0.079
Anderson, Kraus, Galinsky, & Keltner (2012)	6456	0.38	0.04	0.04
Ross, Greene, & House (1977)	6256	-5	0.26	0.26
Ross et al. (1977)	6306	2.3	0.66	0.66
Giessner & Schubert (2007)	6641	-0.083	0.87	0.87
Tversky & Kahneman (1981)	6000	1.3	0.81	0.81
Hauser et al. 2/2 (2007)	6668	-4.6	0.22	0.22
Risen & Gilovich (2008)	6722	-0.13	0.81	0.81
Savani, Markus, Naidu, Kumar, & Berlia (2010)	6722	-3	0.7	0.7
Norenzayan, Smith, Kim, & Nisbett (2002)	6732	5.2	0.36	0.36
Hsee (1998)	6537	-0.097	0.73	0.73
Gray & Wegner (2009)	6720	-0.43	0.37	0.37
Zhong & Liljenquist (2006)	6173	-0.43	0.21	0.21
Schwarz, Strack, & Mai (1991)	6303	0.39	0.5	0.5
Shafir (1993)	6630	-2.3	0.39	0.39
Zaval, Keenan, Johnson, & Weber (2014)	3560	-0.097	0.77	0.77
Knobe (2003)	6707	1.1	0.051	0.051
Tversky & Gati (1978)	2911	-0.0037	0.99	0.99

Supplementary Table 68. **Analysis C**: Matrix model, where participants are clustered by birth country and participants missing birth countries are assumed to be native to the sample country. WEIRDness is operationalizated using CF_{ST}, where countries missing CF_{ST} are excluded from analysis. A *minimum of 100 participants per country* is used as an inclusion criteria. *p* values are adjusted using Bonferroni correction. The values in brackets represent the 2.5% and 97.5% confidence intervals.

Study	*N*	Effect	*p* value	Adjusted *p* value
Huang, Tse, & Cho (2014)	5292	-35	0.45	0.45
Kay, Laurin, Fitzsimons, & Landau (2014)	5365	0.47	0.12	0.12
Alter, Oppenheimer, Epley, & Eyre (2007)	5916	-0.11	0.3	0.3
Graham, Haidt, & Nosek (2009)	6097	0.26	0.93	0.93
Rottenstreich & Hsee (2001)	5959	-0.34	0.47	0.47
Bauer, Wilkie, Kim, & Bodenhausen (2012)	5514	-0.25	0.38	0.38
Miyamoto & Kitayama (2002)	6042	1.5	0.42	0.42
Inbar, Pizarro, Knobe, & Bloom (2009)	5936	-0.055	0.97	0.97
Critcher & Gilovich (2008)	5408	2.4	0.29	0.29
Van Lange, Otten, De Bruin, & Joireman (1997)	5065	-0.087	0.94	0.94
Hauser et al. 1/1 (2007)	5947	1.8	0.067	0.067
Anderson, Kraus, Galinsky, & Keltner (2012)	6073	0.15	0.12	0.12
Ross, Greene, & House (1977)	5788	-0.091	0.99	0.99
Ross et al. (1977)	5699	-3	0.38	0.38
Giessner & Schubert (2007)	6195	0.064	0.7	0.7
Tversky & Kahneman (1981)	5427	-0.093	0.77	0.77
Hauser et al. 2/2 (2007)	6209	0.13	0.75	0.75
Risen & Gilovich (2008)	6270	0.053	0.92	0.92
Savani, Markus, Naidu, Kumar, & Berlia (2010)	6270	-0.032	0.9	0.9
Norenzayan, Smith, Kim, & Nisbett (2002)	6279	8.4	0.13	0.13
Hsee (1998)	6056	-0.0016	0.99	0.99
Gray & Wegner (2009)	6270	0.066	0.89	0.89
Zhong & Liljenquist (2006)	5446	-0.35	0.14	0.14
Schwarz, Strack, & Mai (1991)	5712	-0.13	0.89	0.89
Shafir (1993)	6153	0.13	0.64	0.64
Zaval, Keenan, Johnson, & Weber (2014)	2883	0.031	0.92	0.92
Knobe (2003)	6248	1.1	0.18	0.18
Tversky & Gati (1978)	2184	-0.085	0.82	0.82

Supplementary Table 69. **Analysis C**: Matrix model, where participants are clustered by birth country and participants missing birth countries are assumed to be native to the sample country. WEIRDness is operationalizated using CF_{ST}, where countries missing CF_{ST} are excluded from analysis. A *minimum of 50 participants per country* is used as an inclusion criteria. *p* values are adjusted using Bonferroni correction. The values in brackets represent the 2.5% and 97.5% confidence intervals.

Study	*N*	Effect	*p* value	Adjusted *p* value
Huang, Tse, & Cho (2014)	5763	-43	0.34	0.34
Kay, Laurin, Fitzsimons, & Landau (2014)	5738	0.41	0.1	0.1
Alter, Oppenheimer, Epley, & Eyre (2007)	6290	-0.16	0.38	0.38
Graham, Haidt, & Nosek (2009)	6373	-0.093	0.98	0.98
Rottenstreich & Hsee (2001)	6333	-0.38	0.36	0.36
Bauer, Wilkie, Kim, & Bodenhausen (2012)	5888	-0.14	0.65	0.65
Miyamoto & Kitayama (2002)	6315	1.8	0.23	0.23
Inbar, Pizarro, Knobe, & Bloom (2009)	6310	-0.32	0.78	0.78
Critcher & Gilovich (2008)	5899	3.4	0.17	0.17
Van Lange, Otten, De Bruin, & Joireman (1997)	5464	0.017	0.98	0.98
Hauser et al. 1/1 (2007)	6321	1.4	0.077	0.077
Anderson, Kraus, Galinsky, & Keltner (2012)	6349	0.1	0.34	0.34
Ross, Greene, & House (1977)	6104	0.21	0.96	0.96
Ross et al. (1977)	6360	-0.97	0.84	0.84
Giessner & Schubert (2007)	6698	0.0072	0.97	0.97
Tversky & Kahneman (1981)	6042	-0.43	0.25	0.25
Hauser et al. 2/2 (2007)	6721	0.083	0.83	0.83
Risen & Gilovich (2008)	6784	-0.059	0.88	0.88
Savani, Markus, Naidu, Kumar, & Berlia (2010)	6785	-0.39	0.33	0.33
Norenzayan, Smith, Kim, & Nisbett (2002)	6795	3.7	0.56	0.56
Hsee (1998)	6568	-0.15	0.62	0.62
Gray & Wegner (2009)	6783	-0.026	0.97	0.97
Zhong & Liljenquist (2006)	5983	-0.17	0.52	0.52
Schwarz, Strack, & Mai (1991)	6374	-0.1	0.93	0.93
Shafir (1993)	6669	-0.11	0.71	0.71
Zaval, Keenan, Johnson, & Weber (2014)	3363	-0.0068	0.95	0.95
Knobe (2003)	6764	0.82	0.33	0.33
Tversky & Gati (1978)	2563	-0.065	0.76	0.76

Supplementary Table 70. **Analysis C**: Matrix model, where participants are clustered by birth country and participants missing birth countries are assumed to be native to the sample country. WEIRDness is operationalizated using CF_{ST}, where countries missing CF_{ST} are excluded from analysis. A *minimum of 36 participants per country* is used as an inclusion criteria. *p* values are adjusted using Bonferroni correction. The values in brackets represent the 2.5% and 97.5% confidence intervals.

Study	*N*	Effect	*p* value	Adjusted *p* value
Huang, Tse, & Cho (2014)	5803	-38	0.4	0.4
Kay, Laurin, Fitzsimons, & Landau (2014)	5858	0.24	0.38	0.38
Alter, Oppenheimer, Epley, & Eyre (2007)	6411	-0.34	0.16	0.16
Graham, Haidt, & Nosek (2009)	6495	-0.31	0.85	0.85
Rottenstreich & Hsee (2001)	6455	0.046	0.9	0.9
Bauer, Wilkie, Kim, & Bodenhausen (2012)	6009	-0.2	0.44	0.44
Miyamoto & Kitayama (2002)	6437	3.8	0.039	0.039
Inbar, Pizarro, Knobe, & Bloom (2009)	6396	-0.45	0.74	0.74
Critcher & Gilovich (2008)	6032	0.41	0.93	0.93
Van Lange, Otten, De Bruin, & Joireman (1997)	5630	-0.67	0.46	0.46
Hauser et al. 1/1 (2007)	6406	1	0.24	0.24
Anderson, Kraus, Galinsky, & Keltner (2012)	6471	0.18	0.15	0.15
Ross, Greene, & House (1977)	6274	-3.6	0.52	0.52
Ross et al. (1977)	6360	-0.97	0.77	0.77
Giessner & Schubert (2007)	6784	-0.094	0.56	0.56
Tversky & Kahneman (1981)	6124	-0.32	0.3	0.3
Hauser et al. 2/2 (2007)	6805	0.46	0.16	0.16
Risen & Gilovich (2008)	6869	0.92	0.045	0.045
Savani, Markus, Naidu, Kumar, & Berlia (2010)	6872	1.4	0.74	0.74
Norenzayan, Smith, Kim, & Nisbett (2002)	6883	6.1	0.24	0.24
Hsee (1998)	6652	-0.25	0.33	0.33
Gray & Wegner (2009)	6871	0.21	0.57	0.57
Zhong & Liljenquist (2006)	5983	-0.17	0.52	0.52
Schwarz, Strack, & Mai (1991)	6457	0.44	0.63	0.63
Shafir (1993)	6754	-0.18	0.68	0.68
Zaval, Keenan, Johnson, & Weber (2014)	3540	-0.042	0.79	0.79
Knobe (2003)	6853	2.4	0.015	0.015
Tversky & Gati (1978)	2940	-0.2	0.6	0.6

Supplementary Table 71. **Analysis C**: Matrix model, where participants are clustered by birth country and participants missing birth countries are assumed to be native to the sample country. WEIRDness is operationalizated using CF_{ST}, where countries missing CF_{ST} are excluded from analysis. A *minimum of 10 participants per country* is used as an inclusion criteria. *p* values are adjusted using Bonferroni correction. The values in brackets represent the 2.5% and 97.5% confidence intervals.

Study	*N*	Effect	*p* value	Adjusted *p* value
Huang, Tse, & Cho (2014)	5963	55	0.021	0.021
Kay, Laurin, Fitzsimons, & Landau (2014)	5958	0.43	0.32	0.32
Alter, Oppenheimer, Epley, & Eyre (2007)	6510	-0.4	0.22	0.22
Graham, Haidt, & Nosek (2009)	6595	-1.3	0.28	0.28
Rottenstreich & Hsee (2001)	6555	0.42	0.14	0.14
Bauer, Wilkie, Kim, & Bodenhausen (2012)	6109	1.2	0.054	0.054
Miyamoto & Kitayama (2002)	6537	5	0.012	0.012
Inbar, Pizarro, Knobe, & Bloom (2009)	6489	-0.4	0.67	0.67
Critcher & Gilovich (2008)	6143	7.6	0.18	0.18
Van Lange, Otten, De Bruin, & Joireman (1997)	5715	-0.48	0.42	0.42
Hauser et al. 1/1 (2007)	6541	9.3	0.073	0.073
Anderson, Kraus, Galinsky, & Keltner (2012)	6571	0.39	0.039	0.039
Ross, Greene, & House (1977)	6361	-4.1	0.29	0.29
Ross et al. (1977)	6687	3.5	0.53	0.53
Giessner & Schubert (2007)	7087	-0.13	0.74	0.74
Tversky & Kahneman (1981)	6435	1	0.88	0.88
Hauser et al. 2/2 (2007)	7115	-4.7	0.2	0.2
Risen & Gilovich (2008)	7184	0.18	0.81	0.81
Savani, Markus, Naidu, Kumar, & Berlia (2010)	7186	-2.3	0.77	0.77
Norenzayan, Smith, Kim, & Nisbett (2002)	7198	4.2	0.56	0.56
Hsee (1998)	6984	-0.011	0.97	0.97
Gray & Wegner (2009)	7185	-0.084	0.85	0.85
Zhong & Liljenquist (2006)	6321	0.086	0.82	0.82
Schwarz, Strack, & Mai (1991)	6710	0.53	0.37	0.37
Shafir (1993)	7082	-2.1	0.47	0.47
Zaval, Keenan, Johnson, & Weber (2014)	3759	-0.075	0.78	0.78
Knobe (2003)	7166	1.3	0.033	0.033
Tversky & Gati (1978)	3113	-0.06	0.82	0.82

Supplementary Table 72. **Analysis C**: Matrix model, where participants are clustered by birth country and participants missing birth countries are assumed to be native to the sample country. WEIRDness is operationalizated using CF_{ST}, imputing the CF_{ST} for countries missing cultural distance. A *minimum of 100 participants per country* is used as an inclusion criteria. *p* values are adjusted using Bonferroni correction. The values in brackets represent the 2.5% and 97.5% confidence intervals.

Study	*N*	Effect	*p* value	Adjusted *p* value
Huang, Tse, & Cho (2014)	5292	-35	0.45	0.45
Kay, Laurin, Fitzsimons, & Landau (2014)	5365	0.47	0.15	0.15
Alter, Oppenheimer, Epley, & Eyre (2007)	5916	-0.11	0.3	0.3
Graham, Haidt, & Nosek (2009)	6097	0.26	0.92	0.92
Rottenstreich & Hsee (2001)	5959	-0.34	0.44	0.44
Bauer, Wilkie, Kim, & Bodenhausen (2012)	5514	-0.25	0.38	0.38
Miyamoto & Kitayama (2002)	6042	1.5	0.37	0.37
Inbar, Pizarro, Knobe, & Bloom (2009)	5936	-0.055	0.95	0.95
Critcher & Gilovich (2008)	5408	2.4	0.29	0.29
Van Lange, Otten, De Bruin, & Joireman (1997)	5065	-0.087	0.92	0.92
Hauser et al. 1/1 (2007)	5947	1.8	0.069	0.069
Anderson, Kraus, Galinsky, & Keltner (2012)	6073	0.15	0.098	0.098
Ross, Greene, & House (1977)	5788	-0.091	0.98	0.98
Ross et al. (1977)	5699	-3	0.39	0.39
Giessner & Schubert (2007)	6195	0.064	0.73	0.73
Tversky & Kahneman (1981)	5427	-0.093	0.79	0.79
Hauser et al. 2/2 (2007)	6209	0.13	0.7	0.7
Risen & Gilovich (2008)	6270	0.053	0.91	0.91
Savani, Markus, Naidu, Kumar, & Berlia (2010)	6270	-0.032	0.91	0.91
Norenzayan, Smith, Kim, & Nisbett (2002)	6279	8.4	0.13	0.13
Hsee (1998)	6056	-0.0016	0.99	0.99
Gray & Wegner (2009)	6270	0.066	0.88	0.88
Zhong & Liljenquist (2006)	5446	-0.35	0.14	0.14
Schwarz, Strack, & Mai (1991)	5712	-0.13	0.91	0.91
Shafir (1993)	6153	0.13	0.67	0.67
Zaval, Keenan, Johnson, & Weber (2014)	2883	0.031	0.91	0.91
Knobe (2003)	6248	1.1	0.17	0.17
Tversky & Gati (1978)	2184	-0.085	0.82	0.82

Supplementary Table 73. **Analysis C**: Matrix model, where participants are clustered by birth country and participants missing birth countries are assumed to be native to the sample country. WEIRDness is operationalizated using CF_{ST}, imputing the CF_{ST} for countries missing cultural distance. A *minimum of 50 participants per country* is used as an inclusion criteria. *p* values are adjusted using Bonferroni correction. The values in brackets represent the 2.5% and 97.5% confidence intervals.

Study	*N*	Effect	*p* value	Adjusted *p* value
Huang, Tse, & Cho (2014)	5763	-43	0.34	0.34
Kay, Laurin, Fitzsimons, & Landau (2014)	5738	0.41	0.08	0.08
Alter, Oppenheimer, Epley, & Eyre (2007)	6290	-0.16	0.4	0.4
Graham, Haidt, & Nosek (2009)	6373	-0.093	0.97	0.97
Rottenstreich & Hsee (2001)	6333	-0.38	0.31	0.31
Bauer, Wilkie, Kim, & Bodenhausen (2012)	5888	-0.14	0.68	0.68
Miyamoto & Kitayama (2002)	6315	1.8	0.21	0.21
Inbar, Pizarro, Knobe, & Bloom (2009)	6310	-0.32	0.81	0.81
Critcher & Gilovich (2008)	5899	3.4	0.17	0.17
Van Lange, Otten, De Bruin, & Joireman (1997)	5464	0.017	0.99	0.99
Hauser et al. 1/1 (2007)	6321	1.4	0.079	0.079
Anderson, Kraus, Galinsky, & Keltner (2012)	6349	0.1	0.34	0.34
Ross, Greene, & House (1977)	6104	0.21	0.97	0.97
Ross et al. (1977)	6360	-0.97	0.8	0.8
Giessner & Schubert (2007)	6698	0.0072	0.97	0.97
Tversky & Kahneman (1981)	6042	-0.43	0.24	0.24
Hauser et al. 2/2 (2007)	6721	0.083	0.83	0.83
Risen & Gilovich (2008)	6784	-0.059	0.88	0.88
Savani, Markus, Naidu, Kumar, & Berlia (2010)	6785	-0.39	0.36	0.36
Norenzayan, Smith, Kim, & Nisbett (2002)	6795	3.7	0.58	0.58
Hsee (1998)	6568	-0.15	0.64	0.64
Gray & Wegner (2009)	6783	-0.026	0.95	0.95
Zhong & Liljenquist (2006)	5983	-0.17	0.55	0.55
Schwarz, Strack, & Mai (1991)	6374	-0.1	0.93	0.93
Shafir (1993)	6669	-0.11	0.76	0.76
Zaval, Keenan, Johnson, & Weber (2014)	3363	-0.0068	0.97	0.97
Knobe (2003)	6764	0.82	0.38	0.38
Tversky & Gati (1978)	2563	-0.065	0.75	0.75

Supplementary Table 74. **Analysis C**: Matrix model, where participants are clustered by birth country and participants missing birth countries are assumed to be native to the sample country. WEIRDness is operationalizated using CF_{ST}, imputing the CF_{ST} for countries missing cultural distance. A *minimum of 36 participants per country* is used as an inclusion criteria. *p* values are adjusted using Bonferroni correction. The values in brackets represent the 2.5% and 97.5% confidence intervals.

Study	*N*	Effect	*p* value	Adjusted *p* value
Huang, Tse, & Cho (2014)	5803	-38	0.39	0.39
Kay, Laurin, Fitzsimons, & Landau (2014)	5858	0.24	0.38	0.38
Alter, Oppenheimer, Epley, & Eyre (2007)	6411	-0.34	0.18	0.18
Graham, Haidt, & Nosek (2009)	6495	-0.31	0.88	0.88
Rottenstreich & Hsee (2001)	6455	0.046	0.89	0.89
Bauer, Wilkie, Kim, & Bodenhausen (2012)	6009	-0.2	0.45	0.45
Miyamoto & Kitayama (2002)	6437	3.8	0.048	0.048
Inbar, Pizarro, Knobe, & Bloom (2009)	6396	-0.45	0.72	0.72
Critcher & Gilovich (2008)	6032	0.41	0.93	0.93
Van Lange, Otten, De Bruin, & Joireman (1997)	5630	-0.67	0.46	0.46
Hauser et al. 1/1 (2007)	6406	1	0.24	0.24
Anderson, Kraus, Galinsky, & Keltner (2012)	6471	0.18	0.14	0.14
Ross, Greene, & House (1977)	6274	-3.6	0.55	0.55
Ross et al. (1977)	6360	-0.97	0.81	0.81
Giessner & Schubert (2007)	6784	-0.094	0.56	0.56
Tversky & Kahneman (1981)	6124	-0.32	0.3	0.3
Hauser et al. 2/2 (2007)	6805	0.46	0.17	0.17
Risen & Gilovich (2008)	6869	0.92	0.043	0.043
Savani, Markus, Naidu, Kumar, & Berlia (2010)	6872	1.4	0.74	0.74
Norenzayan, Smith, Kim, & Nisbett (2002)	6883	6.1	0.24	0.24
Hsee (1998)	6652	-0.25	0.33	0.33
Gray & Wegner (2009)	6871	0.21	0.57	0.57
Zhong & Liljenquist (2006)	5983	-0.17	0.54	0.54
Schwarz, Strack, & Mai (1991)	6457	0.44	0.6	0.6
Shafir (1993)	6754	-0.18	0.7	0.7
Zaval, Keenan, Johnson, & Weber (2014)	3540	-0.042	0.79	0.79
Knobe (2003)	6853	2.4	0.014	0.014
Tversky & Gati (1978)	2940	-0.2	0.63	0.63

Supplementary Table 75. **Analysis C**: Matrix model, where participants are clustered by birth country and participants missing birth countries are assumed to be native to the sample country. WEIRDness is operationalizated using CF_{ST}, imputing the CF_{ST} for countries missing cultural distance. A *minimum of 10 participants per country* is used as an inclusion criteria. *p* values are adjusted using Bonferroni correction. The values in brackets represent the 2.5% and 97.5% confidence intervals.

Study	*N*	Effect	*p* value	Adjusted *p* value
Huang, Tse, & Cho (2014)	5963	55	0.026	0.026
Kay, Laurin, Fitzsimons, & Landau (2014)	5958	0.43	0.29	0.29
Alter, Oppenheimer, Epley, & Eyre (2007)	6510	-0.4	0.23	0.23
Graham, Haidt, & Nosek (2009)	6595	-1.3	0.26	0.26
Rottenstreich & Hsee (2001)	6555	0.42	0.14	0.14
Bauer, Wilkie, Kim, & Bodenhausen (2012)	6109	1.2	0.052	0.052
Miyamoto & Kitayama (2002)	6537	5	0.015	0.015
Inbar, Pizarro, Knobe, & Bloom (2009)	6489	-0.4	0.66	0.66
Critcher & Gilovich (2008)	6143	7.6	0.21	0.21
Van Lange, Otten, De Bruin, & Joireman (1997)	5715	-0.48	0.4	0.4
Hauser et al. 1/1 (2007)	6541	9.3	0.08	0.08
Anderson, Kraus, Galinsky, & Keltner (2012)	6571	0.39	0.044	0.044
Ross, Greene, & House (1977)	6361	-4.1	0.3	0.3
Ross et al. (1977)	6687	3.5	0.51	0.51
Giessner & Schubert (2007)	7087	-0.13	0.75	0.75
Tversky & Kahneman (1981)	6435	1	0.87	0.87
Hauser et al. 2/2 (2007)	7115	-4.7	0.2	0.2
Risen & Gilovich (2008)	7184	0.18	0.77	0.77
Savani, Markus, Naidu, Kumar, & Berlia (2010)	7186	-2.3	0.75	0.75
Norenzayan, Smith, Kim, & Nisbett (2002)	7198	4.2	0.55	0.55
Hsee (1998)	6984	-0.011	0.97	0.97
Gray & Wegner (2009)	7185	-0.084	0.86	0.86
Zhong & Liljenquist (2006)	6321	0.086	0.83	0.83
Schwarz, Strack, & Mai (1991)	6710	0.53	0.37	0.37
Shafir (1993)	7082	-2.1	0.48	0.48
Zaval, Keenan, Johnson, & Weber (2014)	3759	-0.075	0.76	0.76
Knobe (2003)	7166	1.3	0.037	0.037
Tversky & Gati (1978)	3113	-0.06	0.8	0.8

Supplementary Table 76. **Analysis C**: Matrix model, where participants are clustered by their country of origin, where country of origin is based on participants stated hometowns. WEIRDness is operationalizated using CF_{ST}, where countries missing CF_{ST} are excluded from analysis. A *minimum of 100 participants per country* is used as an inclusion criteria. *p* values are adjusted using Bonferroni correction. The values in brackets represent the 2.5% and 97.5% confidence intervals.

Study	*N*	Effect	*p* value	Adjusted *p* value
Huang, Tse, & Cho (2014)	5279	-37	0.44	0.44
Kay, Laurin, Fitzsimons, & Landau (2014)	5351	0.43	0.15	0.15
Alter, Oppenheimer, Epley, & Eyre (2007)	5899	-0.11	0.27	0.27
Graham, Haidt, & Nosek (2009)	6054	0.41	0.9	0.9
Rottenstreich & Hsee (2001)	5940	-0.29	0.5	0.5
Bauer, Wilkie, Kim, & Bodenhausen (2012)	5484	-0.25	0.35	0.35
Miyamoto & Kitayama (2002)	6026	1.4	0.39	0.39
Inbar, Pizarro, Knobe, & Bloom (2009)	5919	0.014	0.99	0.99
Critcher & Gilovich (2008)	5396	4.1	0.1	0.1
Van Lange, Otten, De Bruin, & Joireman (1997)	5056	0.24	0.81	0.81
Hauser et al. 1/1 (2007)	5931	1.9	0.057	0.057
Anderson, Kraus, Galinsky, & Keltner (2012)	6053	0.12	0.18	0.18
Ross, Greene, & House (1977)	5773	0.31	0.95	0.95
Ross et al. (1977)	5676	-3.2	0.32	0.32
Giessner & Schubert (2007)	6163	0.068	0.74	0.74
Tversky & Kahneman (1981)	5404	-0.11	0.73	0.73
Hauser et al. 2/2 (2007)	6181	0.16	0.65	0.65
Risen & Gilovich (2008)	6236	0.05	0.91	0.91
Savani, Markus, Naidu, Kumar, & Berlia (2010)	6240	-0.027	0.92	0.92
Norenzayan, Smith, Kim, & Nisbett (2002)	6244	8.6	0.11	0.11
Hsee (1998)	6022	0.036	0.9	0.9
Gray & Wegner (2009)	6236	0.079	0.85	0.85
Zhong & Liljenquist (2006)	5426	-0.36	0.13	0.13
Schwarz, Strack, & Mai (1991)	5684	-0.16	0.89	0.89
Shafir (1993)	6118	0.044	0.86	0.86
Zaval, Keenan, Johnson, & Weber (2014)	2870	0.05	0.86	0.86
Knobe (2003)	6217	1.1	0.16	0.16
Tversky & Gati (1978)	2175	-0.12	0.85	0.85

Supplementary Table 77. **Analysis C**: Matrix model, where participants are clustered by their country of origin, where country of origin is based on participants stated hometowns. WEIRDness is operationalizated using CF_{ST}, where countries missing CF_{ST} are excluded from analysis. A *minimum of 50 participants per country* is used as an inclusion criteria. *p* values are adjusted using Bonferroni correction. The values in brackets represent the 2.5% and 97.5% confidence intervals.

Study	*N*	Effect	*p* value	Adjusted *p* value
Huang, Tse, & Cho (2014)	5748	-43	0.36	0.36
Kay, Laurin, Fitzsimons, & Landau (2014)	5723	0.38	0.1	0.1
Alter, Oppenheimer, Epley, & Eyre (2007)	6272	-0.17	0.35	0.35
Graham, Haidt, & Nosek (2009)	6329	0.017	1	1
Rottenstreich & Hsee (2001)	6313	-0.33	0.34	0.34
Bauer, Wilkie, Kim, & Bodenhausen (2012)	5857	-0.14	0.65	0.65
Miyamoto & Kitayama (2002)	6298	1.7	0.21	0.21
Inbar, Pizarro, Knobe, & Bloom (2009)	6292	-0.26	0.82	0.82
Critcher & Gilovich (2008)	5885	5.4	0.068	0.068
Van Lange, Otten, De Bruin, & Joireman (1997)	5454	0.26	0.77	0.77
Hauser et al. 1/1 (2007)	6304	1.5	0.064	0.064
Anderson, Kraus, Galinsky, & Keltner (2012)	6328	0.072	0.52	0.52
Ross, Greene, & House (1977)	6088	0.41	0.94	0.94
Ross et al. (1977)	6336	-1.2	0.74	0.74
Giessner & Schubert (2007)	6664	0.037	0.85	0.85
Tversky & Kahneman (1981)	6016	-0.48	0.19	0.19
Hauser et al. 2/2 (2007)	6691	0.088	0.81	0.81
Risen & Gilovich (2008)	6748	-0.066	0.87	0.87
Savani, Markus, Naidu, Kumar, & Berlia (2010)	6753	-0.42	0.36	0.36
Norenzayan, Smith, Kim, & Nisbett (2002)	6758	4.2	0.52	0.52
Hsee (1998)	6532	-0.084	0.76	0.76
Gray & Wegner (2009)	6747	-0.0083	0.99	0.99
Zhong & Liljenquist (2006)	5962	-0.15	0.6	0.6
Schwarz, Strack, & Mai (1991)	6342	-0.046	0.95	0.95
Shafir (1993)	6632	-0.14	0.65	0.65
Zaval, Keenan, Johnson, & Weber (2014)	3350	-0.0096	0.96	0.96
Knobe (2003)	6731	0.83	0.32	0.32
Tversky & Gati (1978)	2554	-0.019	0.94	0.94

Supplementary Table 78. **Analysis C**: Matrix model, where participants are clustered by their country of origin, where country of origin is based on participants stated hometowns. WEIRDness is operationalizated using CF_{ST}, where countries missing CF_{ST} are excluded from analysis. A *minimum of 36 participants per country* is used as an inclusion criteria. *p* values are adjusted using Bonferroni correction. The values in brackets represent the 2.5% and 97.5% confidence intervals.

Study	*N*	Effect	*p* value	Adjusted *p* value
Huang, Tse, & Cho (2014)	5788	-38	0.38	0.38
Kay, Laurin, Fitzsimons, & Landau (2014)	5807	0.23	0.5	0.5
Alter, Oppenheimer, Epley, & Eyre (2007)	6357	-0.43	0.15	0.15
Graham, Haidt, & Nosek (2009)	6415	-0.24	0.94	0.94
Rottenstreich & Hsee (2001)	6399	-0.39	0.25	0.25
Bauer, Wilkie, Kim, & Bodenhausen (2012)	5942	-0.14	0.63	0.63
Miyamoto & Kitayama (2002)	6384	1.5	0.29	0.29
Inbar, Pizarro, Knobe, & Bloom (2009)	6378	-0.41	0.73	0.73
Critcher & Gilovich (2008)	6018	2.2	0.61	0.61
Van Lange, Otten, De Bruin, & Joireman (1997)	5584	-0.29	0.79	0.79
Hauser et al. 1/1 (2007)	6389	1.1	0.18	0.18
Anderson, Kraus, Galinsky, & Keltner (2012)	6414	-0.00067	1	1
Ross, Greene, & House (1977)	6222	-4.5	0.5	0.5
Ross et al. (1977)	6336	-1.2	0.76	0.76
Giessner & Schubert (2007)	6741	0.12	0.42	0.42
Tversky & Kahneman (1981)	6091	-0.39	0.2	0.2
Hauser et al. 2/2 (2007)	6767	1.6	0.032	0.032
Risen & Gilovich (2008)	6824	0.031	0.94	0.94
Savani, Markus, Naidu, Kumar, & Berlia (2010)	6831	1.3	0.74	0.74
Norenzayan, Smith, Kim, & Nisbett (2002)	6837	7.1	0.17	0.17
Hsee (1998)	6608	-0.11	0.72	0.72
Gray & Wegner (2009)	6826	0.16	0.65	0.65
Zhong & Liljenquist (2006)	5962	-0.15	0.58	0.58
Schwarz, Strack, & Mai (1991)	6417	0.01	0.99	0.99
Shafir (1993)	6706	-0.17	0.7	0.7
Zaval, Keenan, Johnson, & Weber (2014)	3524	-0.053	0.75	0.75
Knobe (2003)	6810	3	0.011	0.011
Tversky & Gati (1978)	2930	-0.16	0.67	0.67

Supplementary Table 79. **Analysis C**: Matrix model, where participants are clustered by their country of origin, where country of origin is based on participants stated hometowns. WEIRDness is operationalizated using CF_{ST}, where countries missing CF_{ST} are excluded from analysis. A *minimum of 10 participants per country* is used as an inclusion criteria. *p* values are adjusted using Bonferroni correction. The values in brackets represent the 2.5% and 97.5% confidence intervals.

Study	*N*	Effect	*p* value	Adjusted *p* value
Huang, Tse, & Cho (2014)	5947	55	0.029	0.029
Kay, Laurin, Fitzsimons, & Landau (2014)	5941	0.44	0.29	0.29
Alter, Oppenheimer, Epley, & Eyre (2007)	6490	-0.42	0.22	0.22
Graham, Haidt, & Nosek (2009)	6549	-1.1	0.4	0.4
Rottenstreich & Hsee (2001)	6533	0.38	0.18	0.18
Bauer, Wilkie, Kim, & Bodenhausen (2012)	6076	1.1	0.041	0.041
Miyamoto & Kitayama (2002)	6518	5	0.02	0.02
Inbar, Pizarro, Knobe, & Bloom (2009)	6458	-0.59	0.57	0.57
Critcher & Gilovich (2008)	6127	8.3	0.095	0.095
Van Lange, Otten, De Bruin, & Joireman (1997)	5703	-0.42	0.51	0.51
Hauser et al. 1/1 (2007)	6522	9.2	0.1	0.1
Anderson, Kraus, Galinsky, & Keltner (2012)	6548	0.36	0.058	0.058
Ross, Greene, & House (1977)	6343	-5.5	0.25	0.25
Ross et al. (1977)	6655	2.2	0.69	0.69
Giessner & Schubert (2007)	7038	-0.086	0.8	0.8
Tversky & Kahneman (1981)	6386	1	0.88	0.88
Hauser et al. 2/2 (2007)	7061	-4	0.32	0.32
Risen & Gilovich (2008)	7123	-0.21	0.75	0.75
Savani, Markus, Naidu, Kumar, & Berlia (2010)	7129	-2.7	0.72	0.72
Norenzayan, Smith, Kim, & Nisbett (2002)	7136	7.1	0.23	0.23
Hsee (1998)	6924	0.031	0.93	0.93
Gray & Wegner (2009)	7124	-0.14	0.78	0.78
Zhong & Liljenquist (2006)	6287	-0.29	0.5	0.5
Schwarz, Strack, & Mai (1991)	6664	0.22	0.66	0.66
Shafir (1993)	7028	-2.1	0.52	0.52
Zaval, Keenan, Johnson, & Weber (2014)	3740	-0.1	0.69	0.69
Knobe (2003)	7107	1.6	0.019	0.019
Tversky & Gati (1978)	3095	-0.11	0.64	0.64

Supplementary Table 80. **Analysis C**: Matrix model, where participants are clustered by their country of origin, where country of origin is based on participants stated hometowns. WEIRDness is operationalizated using CF_{ST}, imputing the CF_{ST} for countries missing cultural distance. A *minimum of 100 participants per country* is used as an inclusion criteria. *p* values are adjusted using Bonferroni correction. The values in brackets represent the 2.5% and 97.5% confidence intervals.

Study	*N*	Effect	*p* value	Adjusted *p* value
Huang, Tse, & Cho (2014)	5279	-37	0.42	0.42
Kay, Laurin, Fitzsimons, & Landau (2014)	5351	0.43	0.16	0.16
Alter, Oppenheimer, Epley, & Eyre (2007)	5899	-0.11	0.31	0.31
Graham, Haidt, & Nosek (2009)	6054	0.41	0.9	0.9
Rottenstreich & Hsee (2001)	5940	-0.29	0.53	0.53
Bauer, Wilkie, Kim, & Bodenhausen (2012)	5484	-0.25	0.36	0.36
Miyamoto & Kitayama (2002)	6026	1.4	0.37	0.37
Inbar, Pizarro, Knobe, & Bloom (2009)	5919	0.014	0.99	0.99
Critcher & Gilovich (2008)	5396	4.1	0.084	0.084
Van Lange, Otten, De Bruin, & Joireman (1997)	5056	0.24	0.82	0.82
Hauser et al. 1/1 (2007)	5931	1.9	0.071	0.071
Anderson, Kraus, Galinsky, & Keltner (2012)	6053	0.12	0.2	0.2
Ross, Greene, & House (1977)	5773	0.31	0.94	0.94
Ross et al. (1977)	5676	-3.2	0.35	0.35
Giessner & Schubert (2007)	6163	0.068	0.72	0.72
Tversky & Kahneman (1981)	5404	-0.11	0.72	0.72
Hauser et al. 2/2 (2007)	6181	0.16	0.64	0.64
Risen & Gilovich (2008)	6236	0.05	0.92	0.92
Savani, Markus, Naidu, Kumar, & Berlia (2010)	6240	-0.027	0.92	0.92
Norenzayan, Smith, Kim, & Nisbett (2002)	6244	8.6	0.13	0.13
Hsee (1998)	6022	0.036	0.9	0.9
Gray & Wegner (2009)	6236	0.079	0.85	0.85
Zhong & Liljenquist (2006)	5426	-0.36	0.1	0.1
Schwarz, Strack, & Mai (1991)	5684	-0.16	0.89	0.89
Shafir (1993)	6118	0.044	0.88	0.88
Zaval, Keenan, Johnson, & Weber (2014)	2870	0.05	0.86	0.86
Knobe (2003)	6217	1.1	0.2	0.2
Tversky & Gati (1978)	2175	-0.12	0.84	0.84

Supplementary Table 81. **Analysis C**: Matrix model, where participants are clustered by their country of origin, where country of origin is based on participants stated hometowns. WEIRDness is operationalizated using CF_{ST}, imputing the CF_{ST} for countries missing cultural distance. A *minimum of 50 participants per country* is used as an inclusion criteria. *p* values are adjusted using Bonferroni correction. The values in brackets represent the 2.5% and 97.5% confidence intervals.

Study	*N*	Effect	*p* value	Adjusted *p* value
Huang, Tse, & Cho (2014)	5748	-43	0.32	0.32
Kay, Laurin, Fitzsimons, & Landau (2014)	5723	0.38	0.13	0.13
Alter, Oppenheimer, Epley, & Eyre (2007)	6272	-0.17	0.35	0.35
Graham, Haidt, & Nosek (2009)	6329	0.017	0.99	0.99
Rottenstreich & Hsee (2001)	6313	-0.33	0.4	0.4
Bauer, Wilkie, Kim, & Bodenhausen (2012)	5857	-0.14	0.66	0.66
Miyamoto & Kitayama (2002)	6298	1.7	0.21	0.21
Inbar, Pizarro, Knobe, & Bloom (2009)	6292	-0.26	0.83	0.83
Critcher & Gilovich (2008)	5885	5.4	0.058	0.058
Van Lange, Otten, De Bruin, & Joireman (1997)	5454	0.26	0.76	0.76
Hauser et al. 1/1 (2007)	6304	1.5	0.052	0.052
Anderson, Kraus, Galinsky, & Keltner (2012)	6328	0.072	0.52	0.52
Ross, Greene, & House (1977)	6088	0.41	0.92	0.92
Ross et al. (1977)	6336	-1.2	0.79	0.79
Giessner & Schubert (2007)	6664	0.037	0.86	0.86
Tversky & Kahneman (1981)	6016	-0.48	0.22	0.22
Hauser et al. 2/2 (2007)	6691	0.088	0.82	0.82
Risen & Gilovich (2008)	6748	-0.066	0.89	0.89
Savani, Markus, Naidu, Kumar, & Berlia (2010)	6753	-0.42	0.34	0.34
Norenzayan, Smith, Kim, & Nisbett (2002)	6758	4.2	0.53	0.53
Hsee (1998)	6532	-0.084	0.79	0.79
Gray & Wegner (2009)	6747	-0.0083	0.99	0.99
Zhong & Liljenquist (2006)	5962	-0.15	0.62	0.62
Schwarz, Strack, & Mai (1991)	6342	-0.046	0.96	0.96
Shafir (1993)	6632	-0.14	0.65	0.65
Zaval, Keenan, Johnson, & Weber (2014)	3350	-0.0096	0.95	0.95
Knobe (2003)	6731	0.83	0.32	0.32
Tversky & Gati (1978)	2554	-0.019	0.94	0.94

Supplementary Table 82. **Analysis C**: Matrix model, where participants are clustered by their country of origin, where country of origin is based on participants stated hometowns. WEIRDness is operationalizated using CF_{ST}, imputing the CF_{ST} for countries missing cultural distance. A *minimum of 36 participants per country* is used as an inclusion criteria. *p* values are adjusted using Bonferroni correction. The values in brackets represent the 2.5% and 97.5% confidence intervals.

Study	*N*	Effect	*p* value	Adjusted *p* value
Huang, Tse, & Cho (2014)	5788	-38	0.4	0.4
Kay, Laurin, Fitzsimons, & Landau (2014)	5807	0.23	0.52	0.52
Alter, Oppenheimer, Epley, & Eyre (2007)	6357	-0.43	0.14	0.14
Graham, Haidt, & Nosek (2009)	6415	-0.24	0.92	0.92
Rottenstreich & Hsee (2001)	6399	-0.39	0.25	0.25
Bauer, Wilkie, Kim, & Bodenhausen (2012)	5942	-0.14	0.65	0.65
Miyamoto & Kitayama (2002)	6384	1.5	0.28	0.28
Inbar, Pizarro, Knobe, & Bloom (2009)	6378	-0.41	0.72	0.72
Critcher & Gilovich (2008)	6018	2.2	0.59	0.59
Van Lange, Otten, De Bruin, & Joireman (1997)	5584	-0.29	0.8	0.8
Hauser et al. 1/1 (2007)	6389	1.1	0.19	0.19
Anderson, Kraus, Galinsky, & Keltner (2012)	6414	-0.00067	1	1
Ross, Greene, & House (1977)	6222	-4.5	0.54	0.54
Ross et al. (1977)	6336	-1.2	0.76	0.76
Giessner & Schubert (2007)	6741	0.12	0.41	0.41
Tversky & Kahneman (1981)	6091	-0.39	0.2	0.2
Hauser et al. 2/2 (2007)	6767	1.6	0.033	0.033
Risen & Gilovich (2008)	6824	0.031	0.94	0.94
Savani, Markus, Naidu, Kumar, & Berlia (2010)	6831	1.3	0.73	0.73
Norenzayan, Smith, Kim, & Nisbett (2002)	6837	7.1	0.15	0.15
Hsee (1998)	6608	-0.11	0.69	0.69
Gray & Wegner (2009)	6826	0.16	0.66	0.66
Zhong & Liljenquist (2006)	5962	-0.15	0.59	0.59
Schwarz, Strack, & Mai (1991)	6417	0.01	0.99	0.99
Shafir (1993)	6706	-0.17	0.71	0.71
Zaval, Keenan, Johnson, & Weber (2014)	3524	-0.053	0.75	0.75
Knobe (2003)	6810	3	0.014	0.014
Tversky & Gati (1978)	2930	-0.16	0.67	0.67

Supplementary Table 83. **Analysis C**: Matrix model, where participants are clustered by their country of origin, where country of origin is based on participants stated hometowns. WEIRDness is operationalizated using CF_{ST}, imputing the CF_{ST} for countries missing cultural distance. A *minimum of 10 participants per country* is used as an inclusion criteria. *p* values are adjusted using Bonferroni correction. The values in brackets represent the 2.5% and 97.5% confidence intervals.

Study	*N*	Effect	*p* value	Adjusted *p* value
Huang, Tse, & Cho (2014)	5947	55	0.025	0.025
Kay, Laurin, Fitzsimons, & Landau (2014)	5941	0.44	0.3	0.3
Alter, Oppenheimer, Epley, & Eyre (2007)	6490	-0.42	0.22	0.22
Graham, Haidt, & Nosek (2009)	6549	-1.1	0.42	0.42
Rottenstreich & Hsee (2001)	6533	0.38	0.17	0.17
Bauer, Wilkie, Kim, & Bodenhausen (2012)	6076	1.1	0.054	0.054
Miyamoto & Kitayama (2002)	6518	5	0.019	0.019
Inbar, Pizarro, Knobe, & Bloom (2009)	6458	-0.59	0.55	0.55
Critcher & Gilovich (2008)	6127	8.3	0.1	0.1
Van Lange, Otten, De Bruin, & Joireman (1997)	5703	-0.42	0.52	0.52
Hauser et al. 1/1 (2007)	6522	9.2	0.068	0.068
Anderson, Kraus, Galinsky, & Keltner (2012)	6548	0.36	0.04	0.04
Ross, Greene, & House (1977)	6343	-5.5	0.28	0.28
Ross et al. (1977)	6655	2.2	0.67	0.67
Giessner & Schubert (2007)	7038	-0.086	0.82	0.82
Tversky & Kahneman (1981)	6386	1	0.91	0.91
Hauser et al. 2/2 (2007)	7061	-4	0.31	0.31
Risen & Gilovich (2008)	7123	-0.21	0.76	0.76
Savani, Markus, Naidu, Kumar, & Berlia (2010)	7129	-2.7	0.73	0.73
Norenzayan, Smith, Kim, & Nisbett (2002)	7136	7.1	0.23	0.23
Hsee (1998)	6924	0.031	0.94	0.94
Gray & Wegner (2009)	7124	-0.14	0.78	0.78
Zhong & Liljenquist (2006)	6287	-0.29	0.51	0.51
Schwarz, Strack, & Mai (1991)	6664	0.22	0.68	0.68
Shafir (1993)	7028	-2.1	0.5	0.5
Zaval, Keenan, Johnson, & Weber (2014)	3740	-0.1	0.7	0.7
Knobe (2003)	7107	1.6	0.027	0.027
Tversky & Gati (1978)	3095	-0.11	0.62	0.62

2.3.4 Analysis D Results

Supplementary Table 84. A summary of the results for Analyses D, including only studies that are predicted to vary culturally, using a filtering criteria of a minimum 36 participants per country. The significance threshold for the migration status analysis uses the alpha = 0.004 (Slate 1) and alpha = 0.003 (Slate 2) as calculated in the original ML2 analysis using Bonferroni correction for multiple comparisons.

Study	Successful Replication in ML-2	Theoretical Evidence for non-random cultural variation	Theoretical Evidence & Empirical Evidence	WEIRD Moderation found in ML2	A: ML2-WEIRD Analysis	A: CFst Analysis A
Huang, Tse, & Cho (2014)	Yes*	No	No	Yes	0.247 *	-3.28 *
Graham, Haidt, & Nosek (2009)	Yes	Maybe	No	No	–	–
Bauer, Wilkie, Kim, & Bodenhausen (2012)	Yes	Maybe	No	No	-0.00584	0.00511
Miyamoto & Kitayama (2002)	Yes*	Yes	Yes	No	-0.0291	0.27
Hauser, Cushman, Young, Jin, & Mikhail (2007)	Yes	Yes	Maybe	No	0.0735	-0.268
Ross, Greene, & House (1977)	Yes	Maybe	No	No	-0.0413	0.456
Ross et al. (1977)	Yes	Maybe	No	No	-0.00127	0.0276
Tversky & Kahneman (1981)	Yes	No	No	No	0.0453	-0.27
Hauser et al. (2007)	Yes	Yes	Yes	No	0.0303	-0.695 *
Risen & Gilovich (2008)	Yes	Yes	No	No	0.0418	-0.061
Norenzayan, Smith, Kim, & Nisbett (2002)	Yes*	Yes	Yes	Yes	0.166 *	-0.811
Hsee (1998)	Yes	No	No	No	0.0823	-0.137
Gray & Wegner (2009)	Yes	Maybe	Maybe	No	0.0978	-0.547
Schwarz, Strack, & Mai (1991)	No	Maybe	No	No	–	–
Knobe (2003)	Yes	Yes	Yes	Yes	0.214 *	-1.1 *

Supplementary Table 84. A summary of the results for Analyses D, including only studies that are predicted to vary culturally, using a filtering criteria of a minimum 36 participants per country. The significance threshold for the migration status analysis uses the alpha = 0.004 (Slate 1) and alpha = 0.003 (Slate 2) as calculated in the original ML2 analysis using Bonferroni correction for multiple comparisons.

Study	B1: CFst Source Country	B1: Weird Scale	B1: Mean Weird Scale	B2: CFst Birth Country (Filter Missing)	B2: CFst Birth Country (Impute Missing)	B2: CFst Hometown Country
Huang, Tse, & Cho (2014)	-38.8 *	2.5	5.6 *	-26.9	-28.2	-28.1
Graham, Haidt, & Nosek (2009)	-1.55	0.154	0.321	-1.51	-1.64	-1.6
Bauer, Wilkie, Kim, & Bodenhausen (2012)	-0.0664	0.00431	0.0114	0.0531	0.0477	0.0433
Miyamoto & Kitayama (2002)	0.49	-0.000844	-0.0398	0.637	-0.189	0.663
Hauser, Cushman, Young, Jin, & Mikhail (2007)	0.712	-0.00385	-0.00745	-0.341	-0.344	-0.346
Ross, Greene, & House (1977)	-1.67	0.069	0.239	-2.38	-2.78	-2.43
Ross et al. (1977)	0.491	-0.014	0.0288	0.262	0.521	0.829
Tversky & Kahneman (1981)	0.0353	-0.00582	-0.0175	-0.141	-0.144	-0.139
Hauser et al. (2007)	0.11	-0.000219	-0.00902	0.363	0.258	0.126
Risen & Gilovich (2008)	-0.0154	0.00802	0.0174	-0.13	-0.268	-0.154
Norenzayan, Smith, Kim, & Nisbett (2002)	3.43	-0.81 *	-1.58 *	5.83	6.79	6.79
Hsee (1998)	0.0104	0.00658	0.0114	0.189	0.168	0.203
Gray & Wegner (2009)	0.318	-0.0396	-0.0761	0.0685	0.132	0.118
Schwarz, Strack, & Mai (1991)	-0.419	0.0378	0.0817	-0.415	-0.0348	-0.123
Knobe (2003)	0.481 *	-0.0925 *	-0.181 *	0.686	0.968	1.02

Supplementary Table 84. A summary of the results for Analyses D, including only studies that are predicted to vary culturally, using a filtering criteria of a minimum 36 participants per country. The significance threshold for the migration status analysis uses the alpha = 0.004 (Slate 1) and alpha = 0.003 (Slate 2) as calculated in the original ML2 analysis using Bonferroni correction for multiple comparisons.

Study	C: Matrix Model CFst Source Country	C: Matrix Model CFst Birth Country (Filter Missing)	C: Matrix Model CFst Birth Country (Impute Missing)	C: Matrix Model CFst Hometown Country
Huang, Tse, & Cho (2014)	-11.3	-42.5	-38.3	-38.3
Graham, Haidt, & Nosek (2009)	-4.55	-0.282	-0.315	-0.239
Bauer, Wilkie, Kim, & Bodenhausen (2012)	0.158	-0.0952	-0.205	-0.144
Miyamoto & Kitayama (2002)	0.0896	1.65	3.85	1.47
Hauser, Cushman, Young, Jin, & Mikhail (2007)	-3.66	1.02	1.02	1.11
Ross, Greene, & House (1977)	-3.11	-5.66	-3.55	-4.45
Ross et al. (1977)	-2.98	-0.983	-0.966	-1.16
Tversky & Kahneman (1981)	-0.308	-0.248	-0.317	-0.391
Hauser et al. (2007)	-0.101	0.115	0.459	1.62
Risen & Gilovich (2008)	0.00375	0.0893	0.917	0.0311
Norenzayan, Smith, Kim, & Nisbett (2002)	-2.37	1.37	6.09	7.11
Hsee (1998)	-0.152	-0.199	-0.249	-0.105
Gray & Wegner (2009)	0.223	-0.228	0.211	0.165
Schwarz, Strack, & Mai (1991)	0.364	-0.51	0.437	0.0103
Knobe (2003)	-0.24	0.696	2.41	3.03

2.3.5 Analysis E Results

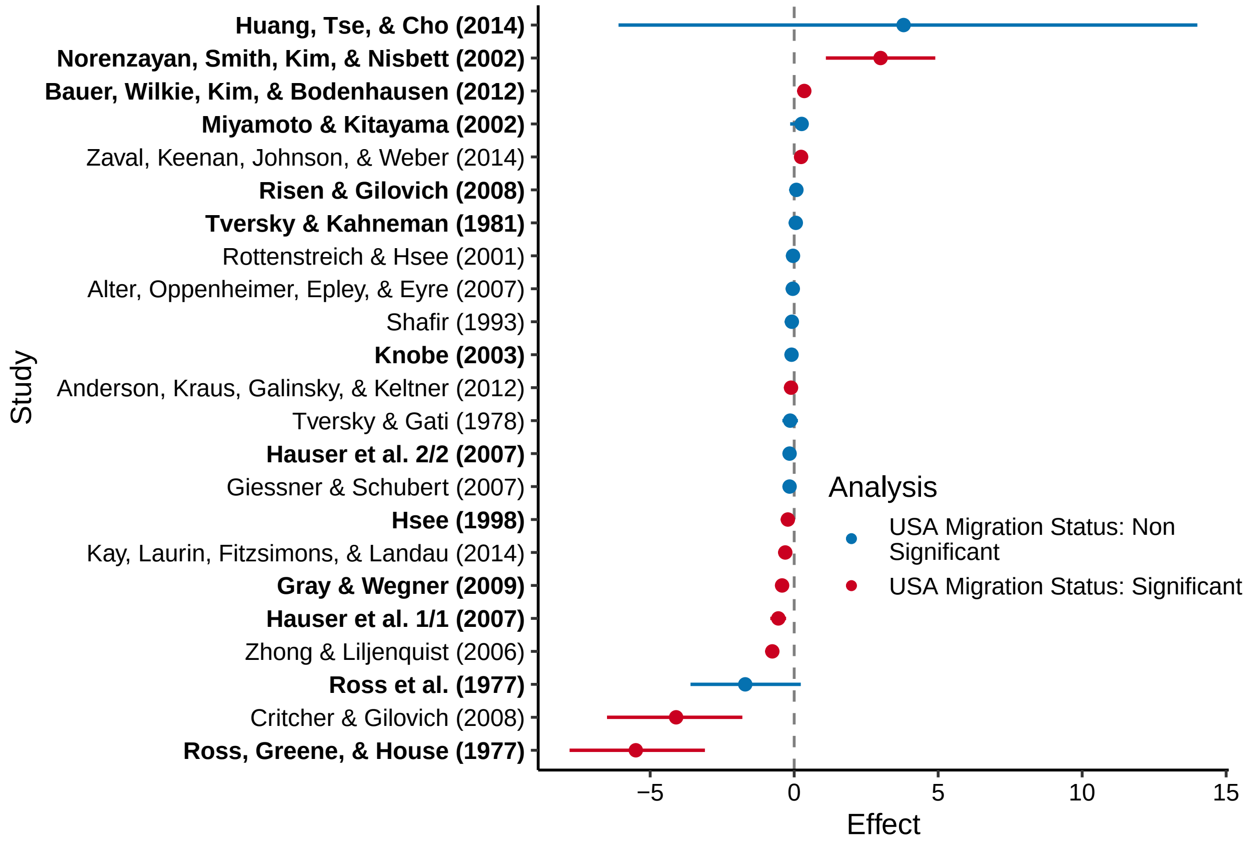

Supplementary Figure 7: Summary results of Analysis E only for sample sites in the USA. Forest plot showing the effect size of migration status, which we added as a control variable to the original ML2 regression models. The significance threshold for the migration status analysis uses the alpha = 0.004 (Slate 1) and alpha = 0.003 (Slate 2) as calculated in the original ML2 analysis using Bonferroni correction for multiple comparisons. Studies in bold replicated in the original ML2 study. The error bars are the 2.5% and 97.5% confidence intervals.

Supplementary Table 85. *Analysis E*: Migrant vs. non migrant analysis of participants in the USA. Participants are clustered by birth country and participants missing birth countries are excluded from analysis. *p* values are adjusted using Bonferroni correction. The values in brackets represent the 2.5% and 97.5% confidence intervals.

Study	*N*	Effect	*p* value	Adjusted *p* value
Huang, Tse, & Cho (2014)	2185	3.8 [-6.1, 14]	4.5e-01	1.0e+00
Kay, Laurin, Fitzsimons, & Landau (2014)	2352	-0.31 [-0.46, -0.16]	4.4e-05	1.0e-03
Alter, Oppenheimer, Epley, & Eyre (2007)	2339	-0.049 [-0.15, 0.053]	3.5e-01	1.0e+00
Rottenstreich & Hsee (2001)	2354	-0.041 [-0.27, 0.19]	7.2e-01	1.0e+00
Bauer, Wilkie, Kim, & Bodenhausen (2012)	2361	0.35 [0.19, 0.52]	1.8e-05	4.1e-04
Miyamoto & Kitayama (2002)	2354	0.26 [-0.14, 0.47]	2.1e-01	1.0e+00
Critcher & Gilovich (2008)	2290	-4.1 [-6.5, -1.8]	6.4e-04	1.5e-02
Hauser, Cushman, Young, Jin, & Mikhail (2007)	2352	-0.55 [-0.83, -0.28]	9.0e-05	2.1e-03
Anderson, Kraus, Galinsky, & Keltner (2012)	2361	-0.11 [-0.18, -0.04]	2.5e-03	5.7e-02
Ross, Greene, & House (1977)	2274	-5.5 [-7.8, -3.1]	4.3e-06	9.9e-05
Ross et al. (1977)	3379	-1.7 [-3.6, 0.23]	8.4e-02	1.0e+00
Giessner & Schubert (2007)	3515	-0.16 [-0.26, -0.048]	4.6e-03	1.1e-01
Tversky & Kahneman (1981)	3543	0.056 [-0.12, 0.23]	5.3e-01	1.0e+00
Hauser et al. (2007)	3527	-0.16 [-0.33, 0.0016]	5.2e-02	1.0e+00
Risen & Gilovich (2008)	3547	0.076 [-0.12, 0.27]	4.4e-01	1.0e+00
Norenzayan, Smith, Kim, & Nisbett (2002)	3555	3 [1.1, 4.9]	2.1e-03	4.7e-02
Hsee (1998)	3547	-0.22 [-0.31, -0.14]	5.0e-07	1.2e-05
Gray & Wegner (2009)	3547	-0.42 [-0.55, -0.28]	0.0e+00	0.0e+00
Zhong & Liljenquist (2006)	3345	-0.76 [-0.88, -0.64]	0.0e+00	0.0e+00
Shafir (1993)	3544	-0.081 [-0.24, 0.08]	3.3e-01	1.0e+00
Zaval, Keenan, Johnson, & Weber (2014)	2244	0.24 [0.15, 0.33]	2.0e-07	3.7e-06
Knobe (2003)	3536	-0.092 [-0.23, 0.05]	2.0e-01	1.0e+00
Tversky & Gati (1978)	1603	-0.14 [-0.41, 0.13]	3.0e-01	1.0e+00

Supplementary Table 86. *Analysis E*: Migrant vs. non migrant analysis of participants in the USA. Participants are clustered by birth country and participants missing birth countries are assumed to be native to the sample country. *p* values are adjusted using Bonferroni correction. The values in brackets represent the 2.5% and 97.5% confidence intervals.

Study	*N*	Effect	*p* value	Adjusted *p* value
Huang, Tse, & Cho (2014)	2192	3.9 [-5.9, 14]	4.4e-01	1.0e+00
Kay, Laurin, Fitzsimons, & Landau (2014)	2361	-0.31 [-0.46, -0.16]	5.3e-05	1.2e-03
Alter, Oppenheimer, Epley, & Eyre (2007)	2348	-0.048 [-0.15, 0.054]	3.6e-01	1.0e+00
Rottenstreich & Hsee (2001)	2364	-0.043 [-0.27, 0.19]	7.1e-01	1.0e+00
Bauer, Wilkie, Kim, & Bodenhausen (2012)	2374	0.36 [0.19, 0.52]	1.6e-05	3.7e-04
Miyamoto & Kitayama (2002)	2362	0.26 [-0.14, 0.49]	2.0e-01	1.0e+00
Critcher & Gilovich (2008)	2298	-4.1 [-6.5, -1.8]	6.3e-04	1.5e-02
Hauser, Cushman, Young, Jin, & Mikhail (2007)	2360	-0.56 [-0.83, -0.28]	8.2e-05	1.9e-03
Anderson, Kraus, Galinsky, & Keltner (2012)	2371	-0.11 [-0.18, -0.039]	2.6e-03	6.0e-02
Ross, Greene, & House (1977)	2282	-5.5 [-7.8, -3.1]	4.3e-06	9.8e-05
Ross et al. (1977)	3404	-1.7 [-3.6, 0.2]	7.9e-02	1.0e+00
Giessner & Schubert (2007)	3544	-0.16 [-0.26, -0.048]	4.6e-03	1.1e-01
Tversky & Kahneman (1981)	3572	0.055 [-0.12, 0.23]	5.4e-01	1.0e+00
Hauser et al. (2007)	3553	-0.16 [-0.33, 0.0048]	5.6e-02	1.0e+00
Risen & Gilovich (2008)	3579	0.072 [-0.12, 0.26]	4.6e-01	1.0e+00
Norenzayan, Smith, Kim, & Nisbett (2002)	3588	3 [1.1, 4.9]	2.1e-03	4.9e-02
Hsee (1998)	3578	-0.22 [-0.31, -0.13]	1.0e-06	2.3e-05
Gray & Wegner (2009)	3579	-0.41 [-0.55, -0.28]	0.0e+00	0.0e+00
Zhong & Liljenquist (2006)	3369	-0.76 [-0.88, -0.65]	0.0e+00	0.0e+00
Shafir (1993)	3575	-0.083 [-0.24, 0.077]	3.1e-01	1.0e+00
Zaval, Keenan, Johnson, & Weber (2014)	2263	0.23 [0.15, 0.32]	2.0e-07	5.3e-06
Knobe (2003)	3566	-0.088 [-0.23, 0.054]	2.2e-01	1.0e+00
Tversky & Gati (1978)	1616	-0.13 [-0.4, 0.13]	3.3e-01	1.0e+00

Supplementary Table 87. *Analysis E*: Migrant vs. non migrant analysis of participants in the USA. Participants are clustered by their country of origin, where country of origin is based on participants stated hometowns. *p* values are adjusted using Bonferroni correction. The values in brackets represent the 2.5% and 97.5% confidence intervals.

Study	*N*	Effect	*p* value	Adjusted *p* value
Huang, Tse, & Cho (2014)	2185	3.7 [-6.2, 14]	4.7e-01	1.0e+00
Kay, Laurin, Fitzsimons, & Landau (2014)	2352	-0.31 [-0.46, -0.16]	4.3e-05	9.9e-04
Alter, Oppenheimer, Epley, & Eyre (2007)	2339	-0.036 [-0.14, 0.067]	4.9e-01	1.0e+00
Rottenstreich & Hsee (2001)	2354	-0.025 [-0.25, 0.2]	8.3e-01	1.0e+00
Bauer, Wilkie, Kim, & Bodenhausen (2012)	2361	0.37 [0.21, 0.53]	8.5e-06	2.0e-04
Miyamoto & Kitayama (2002)	2354	0.22 [-0.18, 0.48]	2.9e-01	1.0e+00
Critcher & Gilovich (2008)	2290	-4 [-6.4, -1.6]	1.0e-03	2.3e-02
Hauser, Cushman, Young, Jin, & Mikhail (2007)	2352	-0.51 [-0.79, -0.23]	3.3e-04	7.6e-03
Anderson, Kraus, Galinsky, & Keltner (2012)	2361	-0.11 [-0.18, -0.034]	4.1e-03	9.4e-02
Ross, Greene, & House (1977)	2274	-5.6 [-7.9, -3.3]	2.9e-06	6.7e-05
Ross et al. (1977)	3380	-1.5 [-3.4, 0.42]	1.3e-01	1.0e+00
Giessner & Schubert (2007)	3516	-0.16 [-0.27, -0.051]	3.9e-03	8.9e-02
Tversky & Kahneman (1981)	3544	0.053 [-0.12, 0.23]	5.5e-01	1.0e+00
Hauser et al. (2007)	3528	-0.16 [-0.32, 0.012]	6.9e-02	1.0e+00
Risen & Gilovich (2008)	3548	0.071 [-0.12, 0.26]	4.7e-01	1.0e+00
Norenzayan, Smith, Kim, & Nisbett (2002)	3556	3.1 [1.2, 5]	1.5e-03	3.4e-02
Hsee (1998)	3548	-0.24 [-0.33, -0.15]	1.0e-07	1.7e-06
Gray & Wegner (2009)	3548	-0.4 [-0.54, -0.27]	0.0e+00	1.0e-07
Zhong & Liljenquist (2006)	3346	-0.77 [-0.89, -0.66]	0.0e+00	0.0e+00
Shafir (1993)	3545	-0.1 [-0.26, 0.062]	2.3e-01	1.0e+00
Zaval, Keenan, Johnson, & Weber (2014)	2244	0.24 [0.15, 0.33]	2.0e-07	4.5e-06
Knobe (2003)	3537	-0.084 [-0.23, 0.058]	2.5e-01	1.0e+00
Tversky & Gati (1978)	1603	-0.14 [-0.41, 0.13]	3.0e-01	1.0e+00

Supplementary References

Arel-Bundock, V., Enevoldsen, N., & Yetman, C. (2018). Countrycode: An R package to convert country names and country codes. *Journal of Open Source Software*, *3*(28), 848. https://doi.org/10.21105/joss.00848

Bates, D., Mächler, M., Bolker, B., & Walker, S. (2015). Fitting Linear Mixed-Effects Models Using **Lme4**. *Journal of Statistical Software*, *67*(1). https://doi.org/10.18637/jss.v067.i01

Borenstein, M., Hedges, L. V., Higgins, J. P. T., & Rothstein, H. R. (2009). *Introduction to Meta-Analysis*. John Wiley & Sons.

Brownrigg, R., Minka, T. P., & Deckmyn, A. (2021). *Maps: Draw Geographical Maps*.

Chan, C., Chan, G. C. H., Leeper, T. J., & Becker, J. (2021). *Rio: A Swiss-army knife for data file I/O*.

Dowle, M., & Srinivasan, A. (2022). *Data.table: Extension of data.frame*.

Gagolewski, M. (2022). **Stringi** : Fast and Portable Character String Processing in *R*. *Journal of Statistical Software*, *103*(2). https://doi.org/10.18637/jss.v103.i02

Goslee, S. C., & Urban, D. L. (2007). The **Ecodist** Package for Dissimilarity-based Analysis of Ecological Data. *Journal of Statistical Software*, *22*(7). https://doi.org/10.18637/jss.v022.i07

Graham, J., Haidt, J., & Nosek, B. A. (2009). Liberals and conservatives rely on different sets of moral foundations. *Journal of Personality and Social Psychology*, *96*(5), 1029–1046. https://doi.org/10.1037/a0015141

Inbar, Y., Pizarro, D. A., Knobe, J., & Bloom, P. (2009). Disgust sensitivity predicts intuitive disapproval of gays. *Emotion*, *9*(3), 435–439. https://doi.org/10.1037/a0015960

Kassambara, A. (2020). *Ggpubr: 'ggplot2' Based Publication Ready Plots*.

Kuznetsova, A., Brockhoff, P. B., & Christensen, R. H. B. (2017). **lmerTest** Package: Tests in Linear Mixed Effects Models. *Journal of Statistical Software*, *82*(13). https://doi.org/10.18637/jss.v082.i13

Muthukrishna, M., Bell, A. V., Henrich, J., Curtin, C. M., Gedranovich, A., McInerney, J., & Thue, B. (2020). Beyond Western, Educated, Industrial, Rich, and Democratic (WEIRD) Psychology: Measuring and Mapping Scales of Cultural and Psychological Distance. *Psychological Science*, *31*(6), 678–701. https://doi.org/ggxkjb

Muthukrishna, M., & Henrich, J. (2019). A problem in theory. *Nature Human Behaviour*, *3*(3), 221–229. https://doi.org/10.1038/s41562-018-0522-1

Pebesma, E. (2018). Simple Features for R: Standardized Support for Spatial Vector Data. *The R Journal*, *10*(1), 439–446. https://doi.org/gf2ztt

Pedersen, T. L. (2022). *Patchwork: The Composer of Plots*.

R Core Team. (2022). *R: A Language and Environment for Statistical Computing*.

Revell, L. J. (2012). Phytools: An R package for phylogenetic comparative biology (and other things): *Phytools: R Package*. *Methods in Ecology and Evolution*, *3*(2), 217–223. https://doi.org/10.1111/j.2041-210X.2011.00169.x

Savani, K., Markus, H. R., Naidu, N. V. R., Kumar, S., & Berlia, N. (2010). What counts as a choice? U.s. Americans are more likely than indians to construe actions as choices. *Psychological Science*, *21*(3), 391–398. https://doi.org/10.1177/0956797609359908

Scheinin, I., Kalimeri, M., Jagerroos, V., Parkkinen, J., Tikkanen, E., Würtz, P., & Kangas, A. (2022). *Ggforestplot: Forestplots of Measures of Effects and Their Confidence Intervals*.

Schwarz, N., Strack, F., & Mai, H.-P. (1991). Assimilation and contrast effects in part-whole question sequences: A conversational logic analysis. *Public Opinion Quarterly*, *55*(1), 3–23. https://doi.org/10.1086/269239

Slowikowski, K. (2021). *Ggrepel: Automatically Position Non-Overlapping Text Labels with 'Ggplot2'*.

South, A. (2017). *Rnaturalearth: World Map Data from Natural Earth*.

Van Lange, P. A. M., De Bruin, E. M. N., Otten, W., & Joireman, J. A. (1997). Development of prosocial, individualistic, and competitive orientations: Theory and preliminary evidence. *Journal of Personality and Social Psychology*, *73*(4), 733–746. https://doi.org/10.1037/0022-3514.73.4.733

Viechtbauer, W. (2010). Conducting Meta-Analyses in *R* with the **Metafor** Package. *Journal of Statistical Software*, *36*(3). https://doi.org/10.18637/jss.v036.i03

Warnes, G. R., Bolker, B., Gorjanc, G., Grothendieck, G., Korosec, A., Lumley, T., MacQueen, D., Magnusson, A., Rogers, J., et al. (2022). *Gdata: Various R Programming Tools for Data Manipulation*.

Wickham, H. (2007). Reshaping Data with the **Reshape** Package. *Journal of Statistical Software*, *21*(12). https://doi.org/10.18637/jss.v021.i12

Wickham, H. (2011a). Testthat: Get Started with Testing. *The R Journal*, *3*(1), 5. https://doi.org/gf9w4r

Wickham, H. (2011b). The Split-Apply-Combine Strategy for Data Analysis. *Journal of Statistical Software*, *40*(1). https://doi.org/10.18637/jss.v040.i01

Wickham, H., Averick, M., Bryan, J., Chang, W., McGowan, L., François, R., Grolemund, G., Hayes, A., Henry, L., Hester, J., Kuhn, M., Pedersen, T., Miller, E., Bache, S., Müller, K., Ooms, J., Robinson, D., Seidel, D., Spinu, V., . . . Yutani, H. (2019). Welcome to the Tidyverse. *Journal of Open Source Software*, *4*(43), 1686.

https://doi.org/10.21105/joss.01686
Wickham, H., Hester, J., & Chang, W. (2022). *Devtools: Tools to Make Developing R Packages Easier*.
